# Population Genomic Analysis in Wild Medaka Populations: Identifying Candidate Loci for Balancing Selection

**DOI:** 10.1002/ece3.74383

**Published:** 2026-10-21

**Authors:** Shingo Fujimoto, Taijun Myosho, Hirozumi Kobayashi, Hiroaki Aoyama, Iki Murase, Bayu K. A. Sumarto, Mitsuharu Yagi, Taiga Kunishima, Masatoshi Matsunami, Ryosuke Kimura

**Affiliations:** ^1^ Integrated Technology Center University of the Ryukyus Okinawa Japan; ^2^ Laboratory of Molecular Reproductive Biology, Institute for Environmental Sciences University of Shizuoka Shizuoka Japan; ^3^ Natural History Museum and Institute Chiba Chiba Japan; ^4^ Ryukyu University Museum Fujukan University of the Ryukyus Okinawa Japan; ^5^ School of Ocean and Earth Science University of Southampton Southampton UK; ^6^ Research Center for Conservation of Marine and Inland Water Resources National Research and Innovation Agency Jakarta Indonesia; ^7^ Graduate School of Fisheries and Environmental Sciences Nagasaki University Nagasaki Japan; ^8^ Faculty of Agriculture Setsunan University Hirakata Osaka Japan; ^9^ Graduate School of Medicine University of the Ryukyus Okinawa Japan

**Keywords:** evolutionary trade‐off, mating behavior, *Oryzias latipes* species complex, population genomics, sexual selection, *Synaptotagmin 4* (*Syt4*)

## Abstract

Sexual selection, defined as individual differences in reproductive success, can contribute to the maintenance of genetic polymorphisms through pleiotropic trade‐offs between life history components. However, the extent to which sexual selection maintains genetic polymorphisms in wild populations remains unclear. Here, we explored genomic signatures of balancing selection and selective sweep in the northern medaka, *Oryzias sakaizumii* in Japan by performing whole‐genome resequencing of wild individuals. In addition, we reevaluated the population genetic structure and admixture of *Oryzias latipes* and *O. sakaizumii* across the Japanese archipelago and identified genomic regions largely affected by introgression between the two species using *F*_*d*_ statistics to distinguish signatures of balancing selection from patterns potentially caused by introgression. Based on multiple population genetic statistics, we identified 18 candidate genomic regions showing signatures of selection. In particular, a 100‐kb region at 6.85–6.95 Mb on chromosome 18 showed high genetic diversity that did not show outlier for the *F*_*d*_ statistics, indicating the extent of introgression in this region did not largely differ from the genome‐wide average. This pattern suggests that balancing selection maintains genetic polymorphisms of the locus in *O. sakaizumii*. The overlap with a QTL for female mating behavior and the known molecular function of a gene within this candidate region for balancing selection suggest that sexual selection through female mate choice may contribute to the maintenance of polymorphism at this locus.

## Graphical abstract

We explored genomic signatures of balancing selection and selective sweep in the northern medaka, *Oryzias sakaizumii* in Japan by performing whole‐genome resequencing of wild individuals. In addition, we reevaluated the population genetic structure and admixture of *Oryzias latipes* and *O. sakaizumii* across the Japanese archipelago and detected genomic regions affected by introgression.

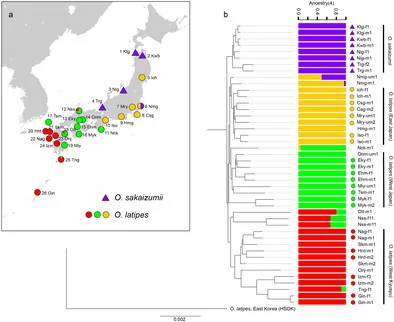

## 1 Introduction

Genetic variation within a population provides information that can be used to assess and detect signatures of natural selection. A selective sweep occurs when one or more favored alleles increase in frequency, often reducing neutral genetic diversity at linked sites (Sabeti et al. 2002; Voight et al. 2006; Kimura et al. 2007; Ferrer‐Admetlla et al. 2014). Conversely, balancing selection maintains genetic diversity through mechanisms such as heterozygote advantage, selective advantages of rare alleles (Hedrick 2007; Fijarczyk and Babik 2015), and antagonistic pleiotropy involving life‐history components (Hedrick 1999; Fijarczyk and Babik 2015; Mérot et al. 2020; Abdul‐Rahman et al. 2021). Genetic polymorphisms maintained by antagonistic pleiotropy have attracted increasing attention in mathematical models, evolutionary experiments, and population genomics, particularly with the development of nucleotide sequencing technologies (Wittmann et al. 2017; Zajitschek and Connallon 2018; Mérot et al. 2020; Abdul‐Rahman et al. 2021). These studies suggest that pleiotropic effects of genotypes on multiple fitness components can maintain genetic polymorphisms across broader regions of the genome than previously recognized (Wittmann et al. 2017; Zajitschek and Connallon 2018; Mérot et al. 2020).

Sexual selection, defined as individual differences in reproductive success, drives the evolution of secondary sexual characters and sexual dimorphism (Andersson and Iwasa 1996). When female mate preferences influence the evolution of male ornamental traits, male and female traits can coevolve through positive frequency‐dependent selection, driving directional changes in their phenotypic values (Mead and Arnold 2004; Andersson and Simmons 2006). However, the direction and magnitude of selection on sexually selected traits may vary across an individual's lifetime (McBride et al. 2015; Simmons et al. 2017; Audzijonyte and Richards 2018; Mérot et al. 2020). Furthermore, the phenotypic optimum often differs between males and females, i.e., sexual conflict (Bonduriansky and Chenoweth 2009; Connallon and Clark 2014; Mank 2017; Zajitschek and Connallon 2018). When the costs and benefits of traits depend on an individual's ontogenetic stage, environmental conditions, and/or sex, the relative fitness of alleles at a locus can change even within a single population (Mank 2017; Wittmann et al. 2017; Mérot et al. 2020). Such context‐dependent fitness effects and antagonistic pleiotropy can promote balancing selection. Indeed, studies of several animal species have provided evidence that genetic variation underlying sexually selected traits can be maintained by balancing selection (Johnston et al. 2013; Horton et al. 2014; Barson et al. 2015; Radwan et al. 2016).

However, whether such patterns of balancing selection are widespread among wild animal populations remains unclear. Because multiple forms of selection can shape phenotypic variation in natural populations, distinguishing between directional selection and balancing selection is often difficult without detailed ecological and fitness data. Genomic signatures of selection can therefore provide complementary evidence about the forms of selection acting in wild populations.

To explore genomic signature of selection in wild populations, demographic events such as population bottlenecks, population substructure, and admixture should be accounted for because demographic events are largely influence the allele frequency spectrum across the genome (Excoffier et al. 2013; Ferrer‐Admetlla et al. 2014; Fijarczyk and Babik 2015; Soni and Jensen 2024), whereas selection is expected to produce more localized genomic signatures. Thus, identifying outliers from empirical genome‐wide distributions of population genetic statistics provides a useful approach for distinguishing candidate regions under selection from genome‐wide demographic effects. However, introgression resulting from historical admixture can locally alter the allele frequency spectrum and potentially produce false positive signatures of selection (e.g., Fijarczyk and Babik 2015; Soni and Jensen 2024). Therefore, historical admixture and introgression should also be considered when interpreting outlier regions identified from empirical genome‐wide distributions of population genetic statistics.

In this study, we focused on two freshwater fish species in Japan, *Oryzias latipes* and *Oryzias sakaizumii*, collectively known as medaka. Medaka females prefer larger male body size and males with longer sexually dimorphic fins (Howard et al. 1998; Fujimoto et al. 2014). Male courtship rate is associated with fertilization success (Weir and Grant 2010) and positively correlates spawning success through mate acquisition (Fujimoto et al. 2015). These observations in mating experiments provide a basis for investigating how sexual selection shapes sexual traits in wild populations.

In addition, geographic variation in secondary sexual traits has been documented, including fin length, male courtship frequency, female mate choice, and egg number (Kawajiri et al. 2015; Shinomiya et al. 2023; Fujimoto et al. 2024, 2026), suggesting that selection pressures on sexual traits may vary among populations and/or species. The genetic architecture of interspecific variation has been evaluated using quantitative trait locus (QTL) analyses in interspecific F_2_ hybrids derived from wild‐caught individuals (Kawajiri et al. 2014, 2015; Fujimoto et al. 2024). Previously identified QTLs underlying interspecific variation in medaka fish span several megabases, with each locus typically containing 100–200 genes (e.g., Kawajiri et al. 2014, 2015; Fujimoto et al. 2024). Therefore, further genetic analyses are required to identify causal genes. In medaka fish and their relatives, molecular basis of mating behavior in both sexes and male‐specific traits has also examined by gene knock‐out strains (e.g., Ansai et al. 2021; Nishiike et al. 2021; Ogino et al. 2023). These studies provide a genetic architecture for identifying genomic regions associated with sexual traits that may have been shaped by sexual selection.

Despite these advances, few studies have investigated signatures of selection in wild medaka populations. A previous selection scan in medaka has been limited to a single collection site of *O. latipes* with a small sample size (Spivakov et al. 2014), and genome‐wide evidence of balancing selection remains lacking. Additionally, although the possibility of adaptation to high‐latitude environments in the northern medaka *O. sakaizumii* has been hypothesized based on their physiology in gonad maturation (Shinomiya et al. 2023) and reproductive ecology in the wild (Fujimoto et al. 2024, 2026), selection scans for this species have not yet been conducted. Therefore, we primarily investigated the signature of selection specifically in wild *O. sakaizumii* population. Although selection scans cannot directly establish genotype–phenotype associations, integrating them with previous genetic studies can help identify candidate genes and polymorphic sites underlying sexually selected traits.

Importantly, wild medaka populations in Japan have experienced secondary contact and admixture (Takehana et al. 2016; Katsumura et al. 2019). Recent studies suggest that admixture between the two *Oryzias* species occurred over a broader geographic range than previously recognized (Fujimoto et al. 2022). Because introgression following secondary contact can produce spurious patterns of signatures of balancing selection, we reevaluated the population genetic structure and the spatial patterns of historical admixture across the Japanese archipelago using whole‐genome resequencing data. Samples collected from multiple sites were used to determine whether historical admixture occurred only locally or whether it predated the diversification of extant populations. These analyses allowed us to identify candidate regions showing signatures of balancing selection, and to assess whether these loci had been substantially affected by historical introgression.

Based on these candidate regions, we discuss the possibility that sexual selection contributes to the maintenance or reduction of genetic variation at previously identified QTLs associated with female mating behavior (chr18:3.71–12.64 M, Fujimoto et al. 2024) and at genes associated with male‐specific morphological traits and courtship behaviors (e.g., Ogino et al. 2023). We further propose a hypothesis for how male–female behavioral interactions may contribute to the maintenance or reduction of genetic polymorphism in this fish.

## 2 Materials and Methods

### 2.1 Fish Populations and Field Collection

Medaka, or the *Oryzias latipes* species complex, comprising *O. latipes* , *O. sinensis* , and *O. sakaizumii*, are small freshwater fish distributed in low‐lying wetlands throughout Japan, Korea, and China (Iwamatsu 2006, Figure 1). Population genetic analyses using nuclear SNVs have shown that the Japanese populations of the *O. latipes* species complex consist of several genetically distinct groups (Katsumura et al. 2019; Fujimoto et al. 2022). *Oryzias latipes* , which is distributed along the coasts of the Pacific and the East China Sea, exhibits a roughly southwest‐to‐northeast genetic cline (Katsumura et al. 2019; Fujimoto et al. 2022). The northern medaka, *O. sakaizumii*, is mainly distributed along the coast of the Sea of Japan (Figure 1a). This genetic group of the *O. latipes* species complex has been used as a model organism in genetics. Reference genome sequences have been constructed for four genetically divergent strains (Ichikawa et al. 2017; Dong et al. 2024).

**FIGURE 1:**
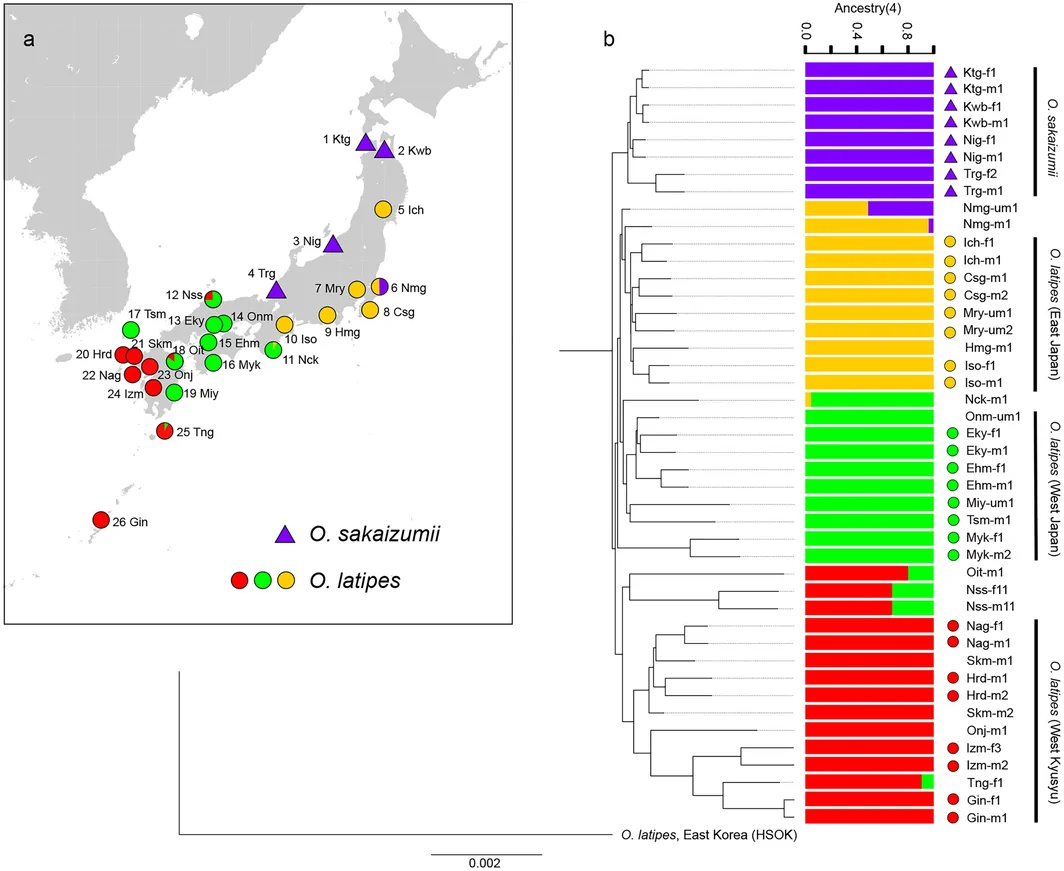
(a) Geographic locations of collection sites. Solid circles and triangles on the map represent *O. latipes* and *O. sakaizumii* , respectively. Colors correspond to the clusters in the ADMIXTURE analysis. (b) Neighbor‐joining tree based on the total whole‐genome sequences and results of ADMIXTURE analysis for *K* = 4. Individuals marked with a circle or triangle were used in the admixture analysis represented in Figure 2. For the abbreviations of the wild populations, see Tables A1 and A2.

To analyze the population structure of wild medaka populations, we collected wild individuals throughout the distribution of the *Oryzias latipes* species complex in Japan (Figure 1a, Table A1). The collection sites covered the major habitats of medaka in Japan. Fish were collected using landing nets measuring approximately 770 cm^2^ with a 2‐mm mesh, and/or four‐armed scoop nets measuring 60 × 60 × 40 cm or 90 × 90 × 55 cm with a 0.5‐mm mesh. At most sites, we obtained whole‐genome sequence data from more than two individuals per site (Tables A1 and A2), including four sites for *O. sakaizumii* and 22 sites for *O. latipes* . Samples collected from multiple sites were used to determine whether historical admixture occurred only locally or whether it predated the diversification of extant populations. Additionally, to examine genetic diversity within *O. sakaizumii*, we obtained sequence data from 8 or 16 individuals per site (*N* = 40). We also used the publicly available sequences of the inbred strain, HSOK (Spivakov et al. 2014, DRA000588), which originated in Korea and was used as an outgroup for the analysis of the Japanese populations.

### 2.2 Library Construction, Sequencing, and Genotyping

For these wild‐collected individuals, live fish were anesthetized with a lethal concentration of MS‐222 (tricaine methanesulfonate, FUJIFILM Wako Pure Chemical Corporation, Osaka, Japan, 500 mg/L) and then dead fish were fixed in 99% ethanol. Genomic DNA was extracted from ethanol‐fixed pectoral fin samples using a DNeasy Kit (Qiagen Inc., Hilden, Germany) according to the manufacturer's instructions. Sample libraries for whole‐genome resequencing were prepared using a TruSeq DNA PCR‐free Library Prep Kit (Illumina Inc., San Diego, CA, USA) according to the manufacturer's instructions. The DNA concentration of each library was measured using a StepOnePlus real‐time PCR system (Thermo Fisher Scientific, Waltham, MA, USA) with a KAPA Library Quantification Kit (Kapa Biosystems Inc., Woburn, MA, USA). Five or six individual libraries were pooled in equal amounts and sequenced using a HiSeq X system (Illumina, PE, 150 bp). Sequencing was performed by Macrogen Japan (Tokyo, Japan). Sequences were deposited in the DDBJ Sequence Read Archive (DRA) under the accession number PRJDB20255.

For each sample, the Illumina adapter sequences were removed, and quality filtering was performed using fastp ver. 0.20.0 with the options “‐‐detect_adapter_for_pe, ‐‐cut_front”. The remaining reads were aligned to the medaka HNI strain reference genome (GCA_002234675.1; ASM223467) using the Burrows‐Wheeler Alignment tool, BWA mem ver. 0.7.17 (Li 2013). HNI was inbred strain, originally collected from the *O. sakaizumii* wild population in Niigata prefecture, and maintained by NBRP Medaka (https://shigen.nig.ac.jp/medaka/). After mapping, the output files were converted to Binary Alignment/Map (BAM) format using SAMtools ver. 1.7 (Danecek et al. 2021). SNVs and InDels were called following the best practice guidelines of the Genome Analysis Tool Kit (GATK ver. 3.8.0) (Auwera and O'Connor 2020). We then filtered out variants based on the following criteria: “QD < 2.0 || FS > 60.0 || MQ < 40.0 || MQRankSum < −12.5 || ReadPosRankSum < −8.0” for SNVs, and “QD < 2.0 || FS > 200.0 || ReadPosRankSum < −20.0” for InDels. Based on the filtered SNVs and InDels, we recalibrated the mapping quality of each read and extracted sequences including variant and nonvariant sites in vcf format using the “GenotypeGVCFs” command in GATK with the “‐‐includeNonVariantSites” option selected.

SNV data were prepared using bcftools (Danecek et al. 2021). For each individual, sequence positions with depths (DP) between 10 and 200, mapping quality (MQ) ≥ 40, and quality by depth (QD) ≥ 2.0 were included in the analysis. Individual vcf files were merged into a multi‐sample vcf file, and sequence positions that were genotyped in all individuals were extracted using the “bcftools view” command with the “NO_MISSING = 0” option. Sites containing InDels were then excluded.

### 2.3 Analysis of Population Structure and Admixture

In total, 271,085,758 bases at nonvariant and variant positions were sequenced from 45 individuals, including eight *O. sakaizumii*, 36 *O. latipes* in Japan, and one inbred strain of *O. latipes* from eastern Korea (HSOK) (Table A2). Phylogenetic relationships were estimated by the neighbor‐joining method using a genetic distance matrix calculated using VCF2Dis ver. 1.47 (Xu et al. 2025). The distance matrix was converted to the nexus format using the “writeDist” function of the “phangorn” package ver. 2.12.1 (Schliep 2011). The phylogenetic tree and network were constructed using SplitsTree ver. 4.15.1 (Hudson and Bryant 2006) and Figtree ver. 1.4.3 (https://github.com/rambaut/figtree/). The branch between the Korean HSOK *O. latipes* from Korea and Japanese populations of *O. sakaizumii* and *O. latipes* was used as the root. To estimate population structure, we used ADMIXTURE ver. 1.3.0 (Alexander et al. 2009). ADMIXTURE was run with the number of clusters (*K*) ranging from 1 to 10, and statistical support for the different numbers of clusters was evaluated based on the lowest cross‐validation error.

To estimate past admixture between populations, *Patterson's D* was calculated for a dataset of 33 individuals, including eight individuals from each population (*O. sakaizumii*: Osak; *O. latipes* : Western Kyushu (WK), Western Japan (WJ), and Eastern Japan (EJ)) and one HSOK individual as an outgroup. We treated HSOK as an outgroup and performed an *ABBA‐BABA* test to calculate *Patterson's D* statistic using “qpDstat” ver. 980 in ADMIXTOOLS (Patterson et al. 2012), which estimates admixture between four populations. For this analysis, SNVs that were polymorphic across the four populations were extracted using the “admixr” package (Petr et al. 2019). In this analysis, individuals with multiple ancestries in the ADMIXTURE results, presumably due to recent migration or admixture, were excluded. For the Osak and EJ populations, which showed signatures of admixture in our initial analysis, we subsequently recalculated *Patterson's D* statistic separately for each collection site to determine whether the inferred admixture signals were restricted to specific collection sites or shared across sites.

To further investigate population admixture, we applied the *F*‐statistics framework (Durand et al. 2011; Patterson et al. 2012; Peter 2016). We reconstructed an admixture graph under an assumed demographic scenario using the “qpGraph” command in ADMIXTOOLS2 (Maier et al. 2023). We selected the best‐fitting model for which the *F*_*4*_ statistic for all the population combinations was less than three. In this analysis, we included five groups (i.e., HSOK, Osak, WK, WJ, EJ(Iso), and EJ(Mry)) in the admixture graph, because the EJ subpopulations appeared to interact differently with other populations, based on the *Patterson's D* statistics.

In addition, introgressed loci were identified using *F*_*d*_ statistics (Martin et al. 2015), a modified version of *Patterson's D* designed for small genomic regions. We calculated *F*_*d*_ statistics using the “Dinvestigate” command in Dsuite ver. 0.5 r46 (Malinsky et al. 2021) in nonoverlapping windows of 50 SNVs. Introgressed regions from the EJ population to the *O. sakaizumii* population were defined as windows in the upper 0.5% tail of the genome‐wide *F*_*d*_ distribution.

### 2.4 Estimation of Population Divergence Time

Divergence between populations was estimated using MSMC2 v.2.0.0 (Schiffels and Wang 2020). To create a mask file specifying the genomic regions to be analyzed in the HNI reference genome sequence, we used Snpable with the option “35mer, *r* = 0.5” (Heng Li, https://lh3lh3.users.sourceforge.net/snpable.shtml). Focusing on sites where two individuals were sequenced from the same site (Figure 1b), we calculated coalescence rates using four individuals (i.e., eight haplotypes) from two populations or from two *O. sakaizumii* collection sites. A VCF file containing four diploid individuals was converted using the script generate_multihetsep.py in msmc‐tools (Stephan Schiffels, https://github.com/stschiff/msmc‐tools/tree/master), and coalescence rates were calculated using the default settings. The relative cross‐population coalescence rate (rCCR) was then calculated, and the time point at which the CCR exceeded 0.5 was used as an indicator of population divergence (Schiffels and Durbin 2014). Coalescence rates were converted to divergence times in generations, assuming a mutation rate per generation of 3.50 × 10^−9^ per generation (Sutra et al. 2019; Malinsky et al. 2018) and a generation time of 1 year.

### 2.5 Genomic Scan to Detect Selection Signatures in *Oryzias sakaizumii*

Focusing on biallelic SNVs in *O. sakaizumii*, *π*, *Tajima's D* (Tajima 1989), and genetic differentiation between males and females (intersexual *FST*) were calculated using pixy (Korunes and Samuk 2021) and VCF‐kit (Cook and Andersen 2017). Each chromosome was divided into nonoverlapping 50,000 bp windows. Based on the empirical distribution of sequenced sites across windows, windows containing fewer than 10,000 sites were excluded from subsequent genomic scan analyses to reduce bias in estimates of population genetic statistics. We identified the top (or bottom) 0.5% tails of the genome‐wide distributions for the statistics (Figure A1). Then, (1) windows with high *π* and high *Tajima's D* as candidates for balancing selection, (2) windows with low *π* and low *Tajima's D* as candidates for SFS‐based signatures of selective sweep, and (3) windows with high *Tajima's D* and high intersexual *FST* as candidates for sex‐specific selection, where different alleles might be more advantageous for survival in each sex, or sex‐association, were extracted (Figure A1a–c).

We also calculated the integrated haplotype score (iHS) in *O. sakaizumii*, which is based on the allele frequency and linkage disequilibrium between SNPs, as candidates for haplotype‐based signature of selective sweep (Figure A1d). Individual haplotypes were estimated from 40 *O. sakaizumii* and 42 *O. latipes* individuals (Table A2) using the “phase_common_static” command in Shapeit4 (Delaneau et al. 2019). Biallelic SNPs with a minor allele frequency greater than 5% in the 40 *O. sakaizumii* individuals were extracted and *Z*‐transformed iHS for each SNP were calculated using Selscan ver. 2.0.0 (Szpiech 2024). For the iHS calculation, we used physical position information instead of fine‐scale recombination map, which is not available for medaka. The top (positive) 0.25% and bottom (negative) 0.25% of *Z*‐transformed iHS values in the genome‐wide distribution were treated as outliers (Figure A1d).

### 2.6 Gene Ontology Analysis and Phenotype Association

To explore the molecular functions of genes located within the regions identified in the genomic scan, we conducted GO enrichment analysis. Gene names and GO annotations were downloaded from Ensembl release 109 (https://feb2023.archive.ensembl.org/) for the *O. latipes* HdrR genome (ASM223467v1, Ichikawa et al. 2017). The HdrR strain was an inbred strain of orange mutant, which was maintained in the National BioResource Project (NBRP Medaka: https://shigen.nig.ac.jp/medaka/). The genetic composition of the nuclear genome in the HdrR strain is similar to that of the Western Japan population of *O. latipes* (WJ) and the Eastern Japan population of *O. latipes* (EJ) (Fujimoto et al. 2022). Since the HdrR genome is better annotated than the HNI genome of *O. sakaizumii* (ASM223471v1, Ichikawa et al. 2017), we used the HdrR genome for this analysis. For example, the HNI reference lacks a comprehensive ortholog database, and gene symbols are undetermined for many Ensembl gene IDs, whereas these annotations are available for the HdrR strain. A list of orthologs between the HNI and HdrR reference sequences was generated using OrthoFinder (Emms and Kelly 2019) and a custom R script. GO enrichment analysis was then performed using HdrR gene annotations and ShinyGO ver. 0.82 (Ge et al. 2020). Enrichment analyses were performed separately for Ensembl gene IDs located in regions with signatures of introgression and selection. Statistical significance was assessed at *FDR* < 0.01.

Detection of selection signatures, such as *Tajima's D* and iHS, depends on haplotype structures in the wild population. The population genetic statistics tended to be inflated in regions with low recombination rates (Stevison and McGaugh 2020), such as the near centromere regions (Peñalba and Wolf 2020). Hence, we inferred the centromere region in HNI reference sequence (ASM223471v1), we used the synteny block information of the chromosomal regions between the HNI and Hd‐rR (Ansai et al. 2022, Table A3). Physical position of synteny block between HNI and Hd‐rR was estimated using ntSynt, v.1.0.0 (Coombe et al. 2024). We examined the extent and overlap of the 1 Mb region near the centromere region (Table A3).

To investigate whether QTLs associated with female mating behavior and male secondary sexual traits (e.g., Kawajiri et al. 2014; Fujimoto et al. 2024) overlapped with signatures of selection, we examined the overlap between these QTLs and candidate genomic windows. For example, QTLs associated with female spawning latency, a measure of mate choosiness, were located on two chromosomes (chr13:10.44–16.24 M, chr18:3.71–12.64 M, Fujimoto et al. 2024). Spawning latency in medaka reflects the time a female takes to accept a male and spawn following male courtship (Okuyama et al. 2014; Fujimoto et al. 2015). The original genetic markers used in the QTL study were mapped to the *O. latipes* HdrR reference genome (Fujimoto et al. 2024). Therefore, genomic positions of the genetic markers were lifted from the HdrR reference (ASM223467v1) and converted to the updated reference genome (ASM223471v1) using the LiftOver software package (Hinrichs et al. 2006). This liftover was performed with “flo” (https://github.com/wurmlab/flo/, Pracana et al. 2017), a pipeline incorporating UCSC tools and BLAT (Kent 2002).

## 3 Results

### 3.1 Analysis of Population Structure and Admixture

Phylogenetic relationships were inferred using the neighbor‐joining method based on whole‐genome sequence data from 45 individuals, including *O. latipes* and *O. sakaizumii* collected from 26 sites across Japan as well as the inbred HSOK strain originating from Korea. The Japanese populations were genetically separated into two species, *O. latipes* and *O. sakaizumii* (Figure 1, Tables A1 and A2). ADMIXTURE analysis supported the presence of four genetic populations (Figure 1b, *K* = 4, CV error = 0.40, Figure A2, Table A4), which correspond to the clusters in the neighbor‐joining tree. Individuals of *O. sakaizumii* collected from four sites were grouped into a single population (hereafter referred to as Osak), while *O. latipes* was separated into three populations: Western Kyushu (WK), Western Japan (WJ), and Eastern Japan (EJ). Some individuals of *O. latipes* collected from Nmg (Namegata), Nck (Nachikatsuura), Nss (Nishihamasata), Oit (Oita), and Tng (Nakatane, Tanegashima) showed multiple genetic ancestries in the ADMIXTURE analysis (Figure 1, Figure A2), likely reflecting recent migration and/or anthropogenic transportation. After removing these admixed individuals, we confirmed that the clusters in the neighbor‐joining tree did not change (Figure 1, Figure A3). In the subsequent admixture analysis, we used only individuals showing a single ancestry, which are marked with circles or triangles in Figure 1b.

We found that *D* (EJ, X; Osak, HSOK), where X is WJ or WK, was significantly positive (|*Z*| = 21.50–30.97), indicating admixture between Osak and EJ (Figure 2b–d, Table A5). Because the four collection sites of Osak (i.e., Tsuruga, Niigata, Aomori, and Kitatsugaru), which are located near the southern and northern edges of the species' distribution, exhibited consistent admixture signals in *D* statistics (Figure 2b–d, Table A5), indicating admixture event between Osak and EJ likely occurred in the ancestral lineage of *O. sakaizumii*. In contrast, when EJ was treated separately by collection site, the *D* values varied. Specifically, *D* (EJ(Iso), EJ2; Osak, HSOK) and *D* (EJ(Iso), EJ2; WJ, HSOK), where EJ2 was EJ(Csg) or EJ(Mry), showed positive and negative values, respectively. These results indicate that EJ(Iso)(and EJ(Ich)) interacted with Osak, whereas EJ(Csg) and EJ(Mry) interacted with WJ. The comparison between WJ and WK, i.e., *D* (WJ, WK, Osak HSOK), also yielded a positive value. Together, these patterns suggest the presence of local admixture between populations, varying by collection site.

**FIGURE 2:**
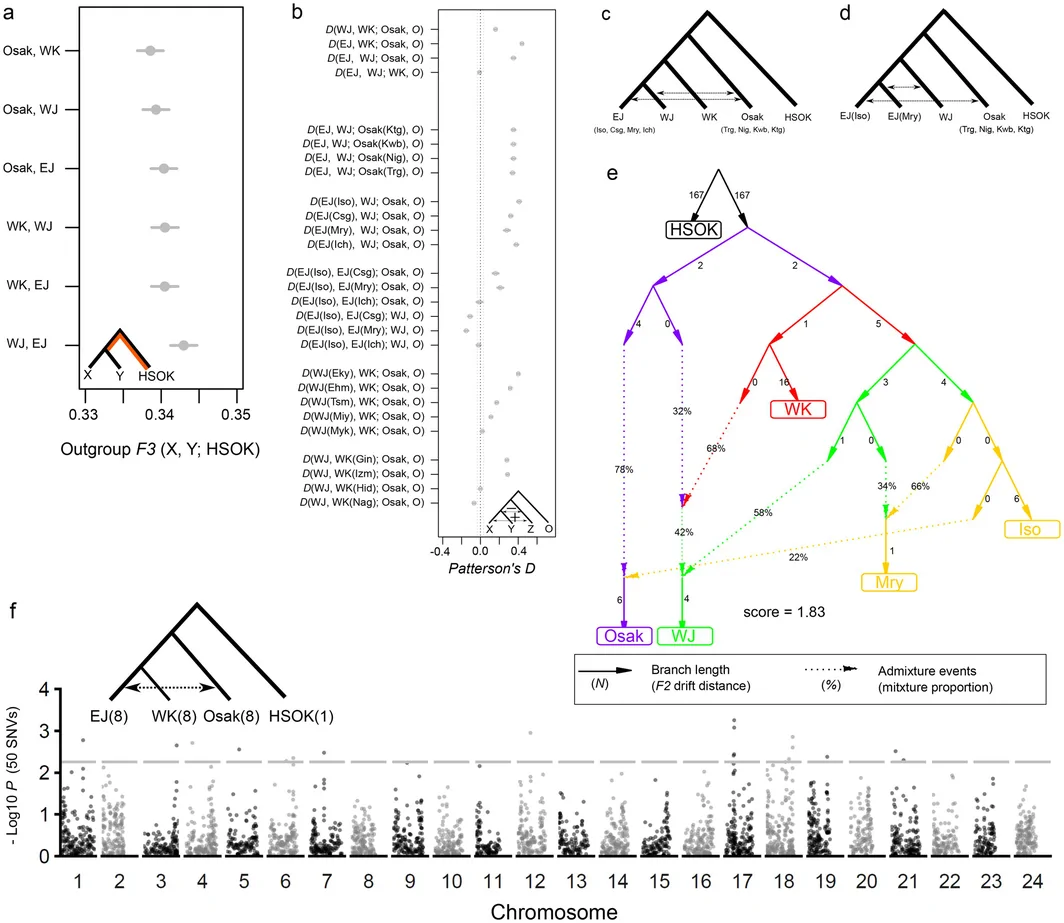
Admixture analyses focusing on *O. sakaizumii* and the Eastern Japan population of *O. latipes* . (a) Outgroup *F*_*3*_ statistics identifying the most closely related populations. (b) Results of *Patterson's D* across all possible combinations among five populations. (c, d) Schematic representations of unadmixed and admixed populations based on *Patterson's D*. (e) Admixture graph best fitting the data. Branch lengths (*F*_*2*_ drift distances) are shown as solid lines, and admixture events are indicated by dotted lines with mixture proportions. (f) Introgressed regions examined by a genomic scan of *F*_*d*_ statistics in 50‐SNV windows (Martin et al. 2015) in the admixture analyses dataset. Y axis represented the −log_10_ transformed *p* value of empirical distribution in *F*_*d*_. Positive values indicated the introgression between EJ and Osak. The numbers in parentheses showed the total sequenced individuals of each population.

We further reconstructed the history of the admixture events of five populations (i.e., HSOK, Osak, WK, WJ, EJ(Iso), and EJ(Mry)) using qpGraph. The resulting graph had a score of 1.83 and the maximum *F* combination was |*Z*| = 1.18 (HSOK, Osak; HSOK, Iso) (Figure 2e, Figure A5). The graph indicated that the interaction between Osak and EJ(Iso) occurred in the direction from EJ(Iso) to Osak, and that EJ(Mry) had received gene flow from the ancestral lineage of WJ. WJ appears to have experienced gene flow from both WK and Osak, although the temporal order of these admixture events remains unresolved (Figure 2e, Figure A5d–f).

Introgressed chromosomal regions from EJ to Osak were assessed using *F*_*d*_ statistics (EJ, WK; Osak, HSOK), calculated within sliding windows of 50 consecutive single‐nucleotide variants (SNVs) along the chromosomes. The *F*_*d*_ values were shifted toward the positive direction across the genome (Figure A6), consistent with the *D* statistic results (Figure 2b). This result indicates that the *F*_*d*_ analysis is robust, even when an inbred individual (HSOK) is used as the outgroup. We interpreted the top 0.5% of windows in the genome‐wide *F*_*d*_ distribution as candidate introgression regions. Outliers of *F*_*d*_ were detected on chromosomes 1, 3, 4, 5, 6, 7, 12, 17, 18, 19, and 21 (Figure 2f, Tables A6 and A7). For example, in the chr6:18.15–18.18 Mb region, we confirmed that eight individuals of *O. sakaizumii* are nested within *O. latipes* , while EJ and *O. sakaizumii* together form a monophyletic clade (Figure A7). The topology showed that alleles in wild *O. sakaizumii* were nearly fixed for sequences closely related to those of the *O. latipes* , EJ, suggesting introgression from *O. latipes* , EJ into *O. sakaizumii*.

To confirm the possible gene functions of 28 annotated genes on the introgressed regions, we identified ortholog groups and performed the GO term enrichment analysis (Table A8), based on reference genome annotations for *O. latipes* (HdrR strain, ASM223467v1), and *O. sakaizumii* (HNI strain, ASM223471v1). The GO analysis indicated an enrichment of genes involved in odor sensing mediated by trace amine‐associated receptors (GO:0001594 trace‐amine receptor activity: *FDR* = 4.1 × 10^−12^, *Fold enrichment* = 280.95, *nGenes* = 7) (Tables A7 and A8); however, these genes are physically clustered on chromosome 21. In addition, an introgressed region on chromosome 6 overlapped with two egg envelope genes, *1‐sf* and *choriogenin H* (GO:0035805 egg coat: *FDR* = 0.0050, *Fold enrichment* = 129.67, *nGenes* = 2), which are homologs of zona pellucida genes in mammals and essential to produce egg envelopes in *O. latipes* (Murata and Kinoshita 2022).

### 3.2 Estimation of Population Divergence Time

Genetic distances between populations were evaluated using outgroup *F*_*3*_ statistics with the eastern Korean population, HSOK, as the outgroup. The closest populations were WJ and EJ, and the most distant were WK and Osak (Figure 2a). Based on MSMC2, the average population divergence times were 2.97 × 10^6^ ± 0.57 × 10^6^ (SD) generations between WK and Osak, 1.01 × 10^6^ ± 0.40 × 10^6^ between WJ and WK, 1.15 × 10^6^ ± 0.50 × 10^6^ between EJ and WK, and 0.48 × 10^6^ ± 0.16 × 10^6^ between EJ and WJ (Figure A4). The average divergence time among the four *O. sakaizumii* collection sites (Tsuruga, Niigata, Aomori, and Kitatsugaru) was estimated to be 3.06 × 10^4^ ± 3.17 × 10^4^ generations. The estimated number of generations within *O. sakaizumii* was two orders of magnitude smaller than those between *O. sakaizumii* and *O. latipes* (Figure A4c,d).

### 3.3 Genomic Scan to Detect Selection Signatures in *Oryzias sakaizumii*

We calculated the integrated haplotype score (iHS) (Figure 3, Table 1, Table A9), and extracted candidate SNPs with haplotype‐based signatures of selective sweeps in *O. sakaizumii* (986 SNPs, Table A9). Of these, 61 SNPs were contained in annotated genes (Table 1). In those SNPs, phosphate‐binding genes tended to be enriched (GO:0032266 phosphatidylinositol‐3‐phosphate binding: *FDR* = 0.0250, *Fold enrichment* = 54.80, *nGenes* = 3, Table A8). SNPs with outlier iHS values were located on some genes whose molecular functions have been well studied in fishes, such as *cd302* (Natural immunity, Brown et al. 2016; Zhang et al. 2022), *BDKRB2* (Cardiovascular system development and locomotor ability, Emanueli et al. 1999), *hoxa13a* (Fin formation and soft fin ray length, Nakamura et al. 2016; Corcoran et al. 2026), and *ar* (androgen receptor beta, Ogino et al. 2023).

**FIGURE 3:**
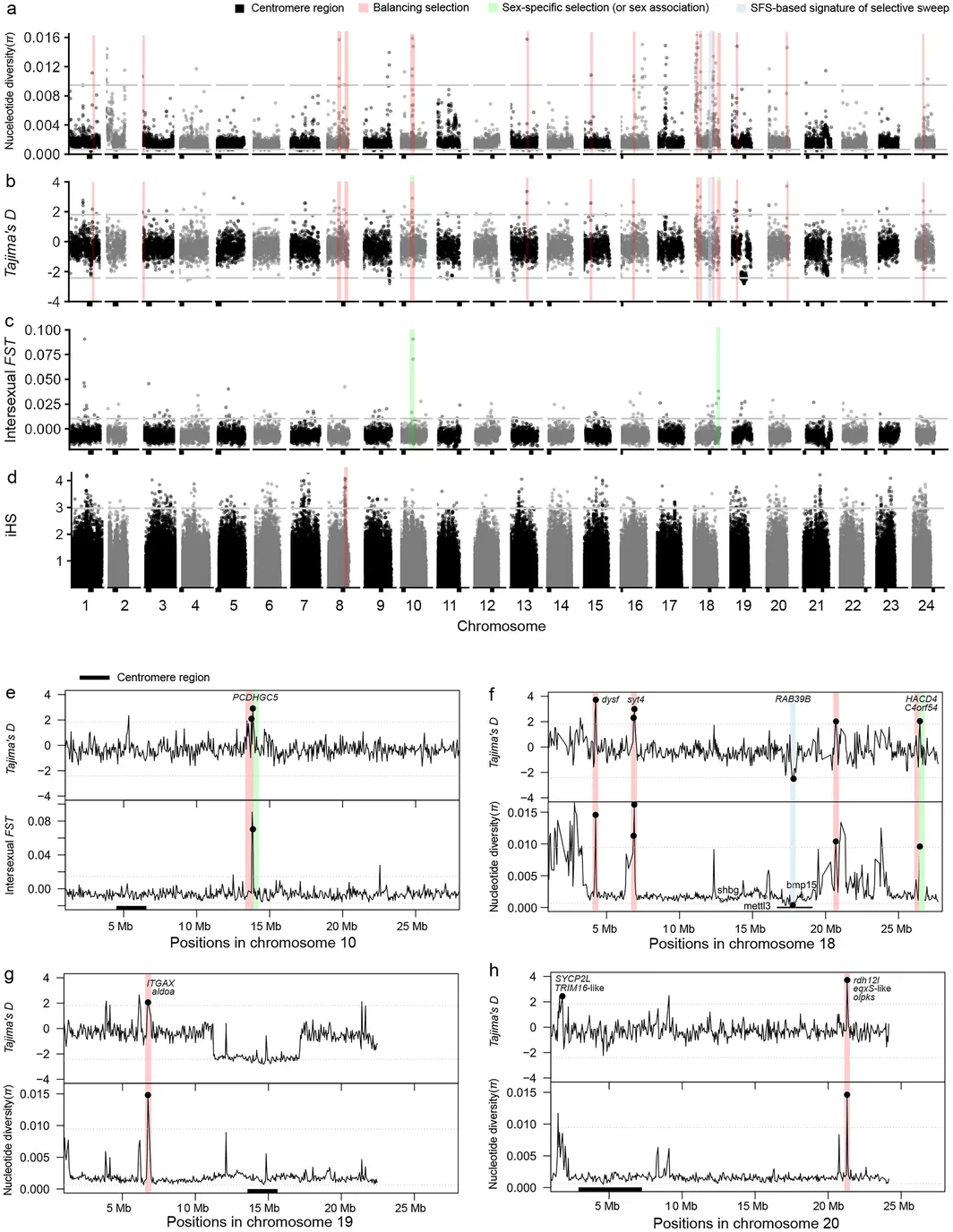
(a–c) Genome‐wide selection scans using nonoverlapping 50,000 bp windows across all 24 chromosomes of wild *O. sakaizumii*, showing nucleotide diversity *π* (a), *Tajima's D* (b), and intersexual *FST* (c). Dotted lines indicate significance thresholds based on genome‐wide distributions. (d) Integrated haplotype scores of 197253 SNPs across all 24 chromosomes. Each dot represents an absolute *Z*‐transformed iHS value. Filled regions indicate genomic intervals with selection signatures in *O. sakaizumii*. (e–h) Selection signatures in chromosome 10, 18, 19, 20, respectively. Gene annotation information was retrieved from Ensembl 109, *Oryzias latipes* (HdrR strain, ASM223467v1). Black bars on X‐axis in each panel represented the 1 Mb region near the inferred centromere region.

**TABLE 1:** Genes containing SNPs with a selective sweep based on integrated haplotype score (iHS) in *O. sakaizumii*.

Chr	Position	*Z*(iHS)	Gene symbol^a^	Chr	Position	*Z*(iHS)	Gene symbol^a^
1	15078135	3.24	*aimp1a*	13	7538965	−2.84	*stard13b*
1	16365897	−3.14	*bnc2*	13	10061825	3.46	*SAMD4B*
1	21528461	3.26	VAV1	14	6420376	3.19	*slc9a6b*
1	29516512	−2.56	*tusc3*	14	6437064	3.32	*snx12*
3	17958199	3.04	*tub*	14	9677044	−2.51	*CHORDC1*
4	7022462	−2.66	*sec22bb*	14	9993944	−2.50	*tpte*
4	7114508	−2.53	*atp5f1d*	14	10012776	−2.65	*vps36*
4	7192745	−2.41	*RNF126*	14	10024107	−2.88	*proser1*
4	14599910	3.17	*ano8b*	14	15103646	3.03	*ar*
4	14658942	2.97	*plvapb*	14	26061922	3.70	*tmem132e*
4	14732376	2.95	*csnk1g2b*	15	21229439	−3.01	*pyroxd2*
5	19850805	−2.78	*cntn2*	16	13258319	3.08	*csmd3a*
7	11801302	3.30	*tmem9*	17	8689397	2.94	*cldn15la*
7	14315027	3.05	*ddx20*	17	8726836	−2.62	*proca*
7	14441227	−2.48	*si:ch211‐117c9.1*	17	20896365	3.28	*ephb1*
7	15002070	2.92	*opn7b*	18	2540664	2.98	*abcg2a*
7	16846152	3.45	*IFT122*	19	1209150	2.95	*cybc1*
8	14853298	2.92	*sec14l1*	19	1209150	2.95	*hexdc*
8	15060746	−3.01	*prpsap1*	19	2660754	2.93	*gb*
8	18737934	3.79	*wbp2nl*	19	8394133	3.15	*fam234a*
8	19631986	3.62	*tom1*	20	15613363	−2.56	*mtfr1*
8	19774597	3.58	*s1pr5a*	21	19548458	3.43	*cd302*
8	19779222	3.78	*spc24*	21	19553123	3.05	*RBMS1*
8	19914577	3.16	*smarca4a*	21	19583315	3.22	*tank*
9	3481874	−2.53	*mrps30*	21	19969093	3.50	*ttc21b*
9	23434909	−2.67	*mxd1*	21	20040713	3.57	*xirp2b*
11	6664530	3.01	*azin1b*	21	20278215	3.14	*bbs5*
11	17437439	−2.90	*hoxa13a*	22	6311060	2.92	*NEMF*
12	6982001	−2.54	*surf6*	22	6475623	2.97	*BDKRB2*
12	6988310	−2.50	*kyat1*	24	8338597	3.00	*smyd2b*
12	7604803	−2.52	*itga1*				

Abbreviation: NA, Not available.

^a^ Gene annotation information was retrieved from Ensembl 109, *Oryzias latipes* , HdrR strain, ASM223467v1 and *O. sakaizumii*, HNI strain, ASM223471v1. These SNPs did not overlapped in centromere regions (Table A3).

We identified 18 candidate genomic regions across 11 chromosomes using multiple population genetic statistics (Table 2, Figure 3, Figure A8). Genomic regions with high *π* and high *Tajima's D* exhibited an enrichment of genes involved in “cell–cell adhesion” (GO:0098609, cell–cell adhesion molecules: *FDR* = 3.0 × 10^−10^, *Fold enrichment* = 18.23, *nGenes* = 12; Tables A7 and A8, significance level: *FDR* < 0.01) and “calcium ion binding” (GO:0005509, calcium ion binding: *FDR* = 0.0022, *Fold enrichment* = 7.811, *nGenes* = 7; Tables A7 and A8). The genes associated with “cell–cell adhesion” included protocadherin alpha family and Integrin alpha‐M‐like (Table A7). The genes associated with “calcium ion binding” included synaptotagmin4 *syt4* (Tables A8 and A10), which functions in synaptic development and dopamine transport (Delignat‐Lavaud et al. 2022; Kim et al. 2024). Of the regions showing high *π* and high *Tajima's D*, two regions on chromosomes 10 and 18 also exhibited high intersexual *FST* (Figure 3, Table 2, Table A7).

**TABLE 2:** Outlier regions of genetic diversity based on nucleotide diversity *π*, *Tajima's D*, intersexual *FST*, and integrated haplotype score (iHS) within *O. sakaizumii*.

High genetic diversity regions by genome scan							Integrated haplotype score (iHS)		
Chr	Start	End (bp)	*π*	*Tajima's D*	Intersexual *FST*	*F*_*d*_	Outlier SNPs	Max |iHS|	Max |iHS| position
1	26000001	26050000^a^	0.011*	2.1709*	0.0000	0.3465	0	1.73	26040924
3	1	50000^a^	0.011*	1.974*	−0.0010	0.0491	NA	NA	NA
8	14350001	14400000^a^	0.016*	2.267*	−0.005	−0.111	0	1.39	14382920
8	19750001	19800000^a^	0.010*	2.139*	−0.0121	0.0324	34	4.14	19779802
10	13750001	13800000^a^	0.012*	2.086*	−0.006	−0.028	0	2.01	13798063
10	13850001	13900000^a^	0.016*	2.908*	0.070*	−0.019	0	2.31	13878057
13	18550001	18600000^a^	0.016*	3.363*	−0.013	−0.152	0	1.80	18596925
15	8050001	8100000	0.011*	2.583*	−0.009	0.029	0	1.90	8097458
16	14400001	14450000	0.011*	2.649*	−0.012	0.017	0	1.66	14445917
18	4250001	4300000	0.015*	3.719*	−0.011	−0.083	0	1.70	4278760
18	6850001	6900000	0.011*	2.302*	−0.011	−0.090	0	2.23	6884357
18	6900001	6950000^a^	0.016*	2.993*	−0.002	0.004	0	2.43	6944225
18	17800001	17850000^b^	**0.0003** ^**†**^	**−2.486** ^**†**^	0.001	−0.020	NA	NA	NA
18	20700001	20750000^a^	0.0103*	1.913*	−0.008	−0.169	0	1.72	21303987
18	26450001	26500000^a^	0.010*	2.049*	0.031*	−0.062	0	1.51	26489032
19	6750001	6800000^a^	0.015*	2.067*	−0.007	0.017	0	1.87	6781462
20	21300001	21350000^a^	0.015*	3.719*	−0.005	0.106	0	1.71	21303987
24	9500001	9550000	0.009*	2.738*	−0.010	−0.066	0	1.01	9511329

*Note:* Bold texts with * and † represented top 0.5% and bottom 0.5%, respectively, of the empirical distribution of the statistics. NA: No SNPs to calculate iHS values in the window.

Abbreviation: NA, not available.

^a^ Genomic regions falling within the upper 2.5% of the empirical genome‐wide distributions of nucleotide diversity (*π*) or *Tajima's D*, calculated using individuals collected at a single site (Tsuruga; *N* = 16).

^b^ Window overlapped in the centromere regions.

Because population subdivision can generate patterns similar to signatures of balancing selection, we further examined these signatures within a single collection site of *O. sakaizumii* (Tsuruga, *N* = 16, Table 2). For example, a candidate region for balancing selection on Chr18:4.25–4.30 M was no longer an outlier in the single‐site analysis (Figure A9, Table 2), indicating that the signal in this region was sensitive to geographic sampling. In contrast, 12 of 18 regions identified as π outliers and 8 of 18 regions identified as *Tajima's D* outliers also remained outliers in the single‐site analysis, suggesting that the high genetic diversity at several candidate regions cannot be explained solely by population subdivision (Table 2).

In the search for signatures of selection, false positives are likely to occur in regions with low recombination rates, such as the near centromere regions (Stevison and McGaugh 2020; Peñalba and Wolf 2020). Based on the centromere region of *O. latipes* , Hd‐rR (Ichikawa et al. 2017; Furuyama et al. 2019; Ansai et al. 2022, Table A3), we estimated the centromere region of *O. sakaizumii* and examined the overlap between the signatures of selection and the 1 Mb region near the centromere (Table 2, Table A9). Of the 984 SNPs with outlier iHS values, 58 SNPs were located within the 1 Mb region near the centromere regions (Table A9). None of the regions with balancing selection or sex‐specific selection detected in our analyses were near the centromere (Table 2). The region with the signature of low genetic diversity (chr18:17.71–18.07 M) overlapped with the centromere region (Figure 3f, Table 2), indicating the signature may have resulted from low recombination and genetic drift, rather than a complete selective sweep.

## 4 Discussion

### 4.1 Population Structure Shaped by Isolation and Introgression

The admixture analyses revealed a complex population history involving isolation and secondary contact within the *O. latipes* species complex (Figure 2e, Figure A5). Our results indicate that admixture occurred between *O. sakaizumii* and part of the EJ population of *O. latipes* (Figure 2). Because the four collection sites of *O. sakaizumii* (i.e., Tsuruga, Niigata, Aomori, and Kitatsugaru), which are located near the southern and northern edges of the species' distribution (Figure 2b–d, Table A5), this admixture event likely occurred in the ancestral lineage of *O. sakaizumii*. Based on estimated divergence times, the ancestral lineage of *O. sakaizumii* dates between 3.06 × 10^4^ and 4.80 × 10^5^ generations ago (Figure A4), *O. sakaizumii* is estimated to have dispersed over a range of 700 km within eastern mainland Japan during the past 30,000 years (Figure 1, Figure A4), suggesting that wild medaka populations possess substantial dispersal ability during initial colonization (Figures 1 and 2, Figure A4, see also, Katsumura et al. 2019). Long‐distance dispersal and/or admixture after secondary contact has also been reported in congeneric species distributed in the Indonesian islands (Sutra et al. 2019; Horoiwa et al. 2021; Montenegro et al. 2022; Flury et al. 2023). The admixture graph also revealed complex secondary contacts among *O. latipes* populations (Figure 2e, Figure A5), supporting the existence of gene flow among geographic populations.

If the ranges of geographic populations in Japanese archipelago are not constrained by dispersal ability, it remains unclear which factors have maintained the distinct geographic population structure observed today (Figures 1 and 2). Based on the genetic variation in juvenile growth rates and female reproductive traits, latitudinal adaptation to climatic environments has been proposed in *O. sakaizumii* and *O. latipes* (Kawajiri et al. 2009; Shinomiya et al. 2023; Fujimoto et al. 2024, 2026). Thus, natural selection and life‐history adaptation to local climate may limit gene flow between geographic populations. Similar patterns of genetic differentiation associated with local adaptation have been reported for other widely distributed fish species distributed across the Japanese archipelago using genome sequence data (e.g., Hirase et al. 2025).

### 4.2 Genes in Candidate Regions Identified by Selection Scans

Previous selection scans in medaka fish in Japan have focused only on a single collection site of *O. latipes* (Spivakov et al. 2014; Fitzgerald et al. 2022). Based on the geographic location of the collection site, the population in previous studies is considered to correspond to the Eastern Japan (EJ) population of *O. latipes* (Figure 1, Katsumura et al. 2019). In this study, we investigated chromosomal regions exhibiting signatures of natural or sexual selection in wild *O. sakaizumii* for the first time. In the scan for selective sweeps by iHS, 61 SNPs located within genes were detected (Table 1).

Some iHS outliers are located on genes associated with the male‐specific morphology and courtship behaviors (Table 1, Table A10). For example, androgen receptor beta is known to regulate the expression of male‐specific traits in *O. latipes* (Ogino et al. 2023). In the zebrafish, *hoxa13a* affects fin formation and soft fin ray length in concert with its paralogs, *hoxa13b* and *hoxd13a* (Corcoran et al. 2026). In addition, iHS outliers on chromosome 7 are sporadically distributed along 11.73–15.60 Mb (Figure 3, Table A9). This region falls within a locus associated with fin morphology (Chr7:9.86–17.10 Mb) in a genome‐wide association study of ornamental medaka strains (Kon et al. 2026). Medaka exhibits sexual dimorphisms in anal and dorsal fin lengths, and males with longer fin lengths are more readily accepted by females in *O. sakaizumii* (Fujimoto et al. 2014, 2021). Sexual selection via female mate choice may lead to selective sweeps at loci associated with fin morphology through increased male reproductive success in wild populations.

The interpretation of selection signatures based on haplotype structure or the site frequency spectrum (SFS) can be confounded by local variation in recombination rates as well as introgression‐associated changes in local genealogies (Soni and Jensen 2024). Although linkage maps based on crosses between *O. latipes* and *O. sakaizumii* are available (e.g., Naruse et al. 2004; Kawajiri et al. 2014; Furuyama et al. 2019), their resolution is insufficient for our selection scans. We therefore examined the overlap of candidate regions with centromeres, where recombination may be broadly suppressed (Tables A3 and A9), and used *F*_*d*_ to evaluate local excess allele sharing produced by introgression (Figure 2f, Table A9). However, these approaches cannot account for fine‐scale variation in recombination rates in wild populations or contribution of historical introgression in regions without *F*_*d*_ outliers. Further evaluation of these effects will require fine‐scale recombination maps and haplotype‐resolved analyses of local genealogical relationships among populations and species (Martin et al. 2015).

### 4.3 Genetic and Phenotypic Features of Chromosome 18

Our selection scan for regions with high *π* and high *Tajima's D* identified chromosomal regions with high genetic diversity in wild *O. sakaizumii* (Figure 3, Table 2, Table A10). Six windows with high genetic diversity were sporadically distributed on chromosome 18. The enrichment of these high diversity windows on chromosome 18 was statistically significant after accounting for differences in the number of analyzed windows among chromosomes (one‐sided hypergeometric test, Bonferroni‐corrected *p* = 0.00068, fold enrichment = 9.18, Gel et al. 2016), indicating that candidate regions with high genetic diversity are disproportionately concentrated on chromosome 18.

Previous genetic analyses using *O. sakaizumii* collected from Aomori (Figure 1) have also identified regions on chromosome 18 associated with sex determination and female mating behavior. Kitano et al. (2023) identified a sex‐differentiated region on chromosome 18 in an experimentally evolved population and suggested that this region has a minor effect on male sex determination (Kitano et al. 2023, Chr18:9.98–25.22 M in HdrR strain reference, corresponding to Chr18:9.00–21.83 M in this study). Furthermore, QTL analysis of F_2_ individuals from interspecific hybrids identified a QTL for spawning latency, an index of female mate choice, on chromosome 18 (chr18:3.71–12.64 M, Fujimoto et al. 2024). In the present study, two high‐diversity regions (Chr18:4.25–4.30 M, Chr18:6.85–6.95 M) overlapped this QTL (Table A10).

### 4.4 A Hypothesis Linking Balancing Selection to Female Mate Choice

Among the candidate regions for balancing selection, chr18:6.85–6.95 M overlaps a previously identified QTL for female spawning latency in medaka (chr18:3.71–12.64 M, Fujimoto et al. 2024, Table A10). Notably, elevated *π* in this region persisted in the single‐site analysis, although *Tajima's D* was not markedly elevated (Figure A9). This region contains *syt4*, which provides a potential link between genetic variation in this region and female mate choice. In mice, *Syt4* protein regulates oxytocin release (Zhang et al. 2011), while in medaka, females alter their sexual receptivity and mating latency in response to familiarity with males (Okuyama et al. 2014), with this familiarity‐dependent response regulated by oxytocin‐Oxtr1 signaling (Yokoi et al. 2020). These findings lead us to hypothesize that genetic variation in *syt4* may affect spawning latency through modulation of oxytocin signaling. However, the relationship between *Syt4* and oxytocin release has not been examined in fishes, and further studies are needed to test this proposed proximate mechanism underlying spawning latency.

If genetic variation in this candidate region affects female spawning latency, balancing selection could maintain such variation when female mate choice involves a trade‐off between its benefits and costs (e.g., Pomiankowski and Iwasa 1998). Females preferentially mate with larger, dominant males, which also mate frequently (Howard et al. 1998; Fujimoto et al. 2015), potentially leading to sperm depletion and reduced fertilization success (Weir and Grant 2010; Kondo et al. 2025). We hypothesize that variation in female spawning latency may reflect a trade‐off between mate assessment and the risk of mating with sperm‐depleted males later in the spawning period. Under this hypothesis, the fitness consequences of spawning latency are predicted to depend on male mating history and/or sperm availability. Testing these predictions will be necessary to determine whether proposed mechanism contributes to the maintenance of genetic variation in wild medaka.

## Author Contributions

**Shingo Fujimoto:** conceptualization (lead), data curation (lead), formal analysis (lead), funding acquisition (lead), investigation (lead), methodology (lead), project administration (lead), resources (lead), software (lead), supervision (supporting), validation (lead), visualization (lead), writing – original draft (lead), writing – review and editing (lead). **Taijun Myosho:** conceptualization (equal), data curation (equal), funding acquisition (equal). **Hirozumi Kobayashi:** data curation (supporting), investigation (supporting). **Hiroaki Aoyama:** data curation (equal), methodology (equal), resources (equal), writing – review and editing (supporting). **Iki Murase:** data curation (equal), investigation (equal), resources (supporting), writing – review and editing (supporting). **Bayu K. A. Sumarto:** data curation (equal), investigation (equal), validation (equal). **Mitsuharu Yagi:** data curation (equal), investigation (equal). **Taiga Kunishima:** data curation (equal), investigation (equal). **Masatoshi Matsunami:** formal analysis (equal), methodology (equal). **Ryosuke Kimura:** formal analysis (equal), funding acquisition (supporting), methodology (equal), project administration (equal), writing – review and editing (equal).

## Funding

This work was supported by Japan Society for the Promotion of Science, 24K09610, 19K16232; Nakatsuji Foresight Foundation; University of the Ryukyus.

## Ethics Statement

All methods related to the handling of live fish were conducted in accordance with the Regulations for Animal Experiments at University of the Ryukyus. All experiments were approved by the Animal Care Ethics Committee of University of the Ryukyus (Approval numbers: R2019035, R2021012, and R2024022). All experimental methods are reported in accordance with ARRIVE guidelines.

## Conflicts of Interest

The authors declare no conflicts of interest.

## Figures and Tables

**FIGURE A1:**
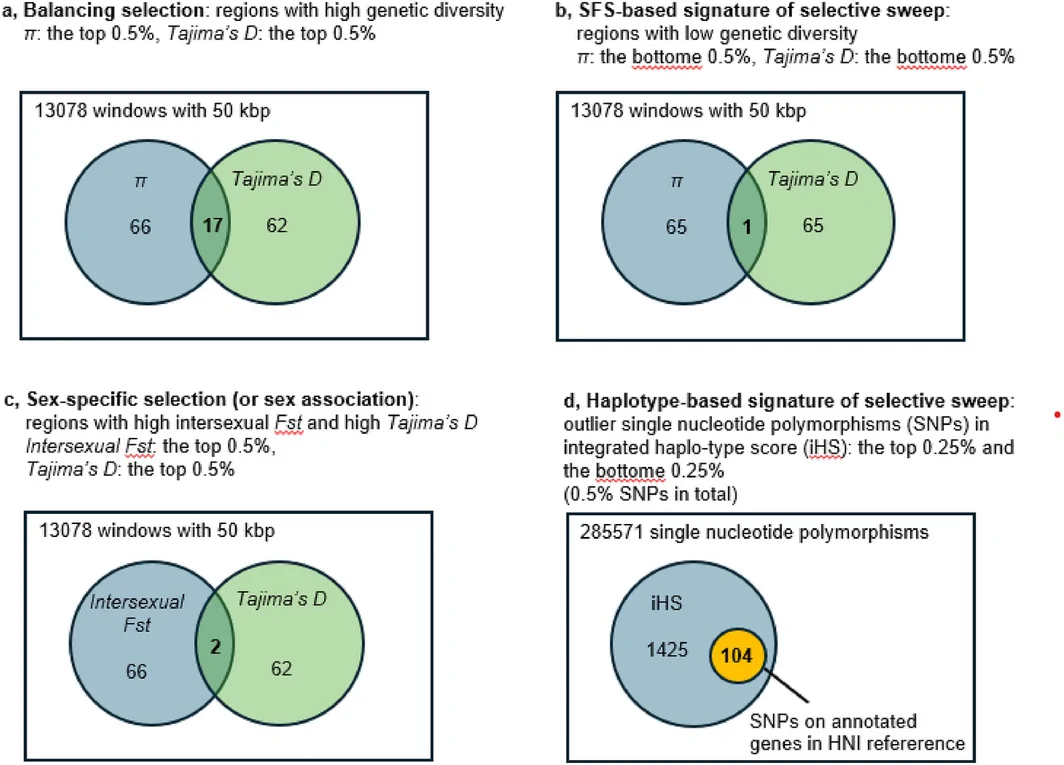
Criteria and the numbers of extracted windows and SNPs in selection signatures.

**FIGURE A2:**
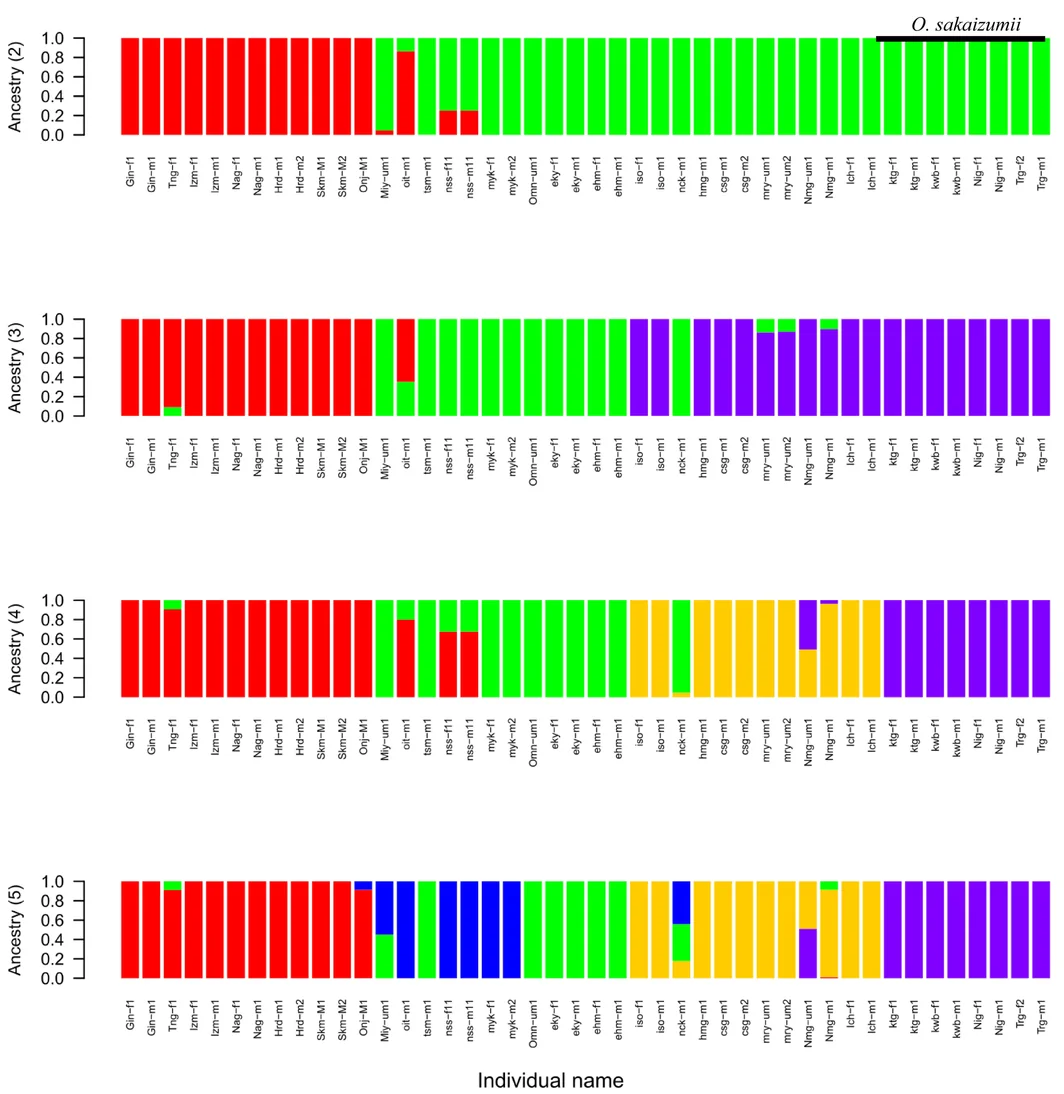
Results of ADMIXTURE analysis for *K* = 2–5. For the abbreviations of wild populations, see Tables A1 and A2.

**FIGURE A3:**
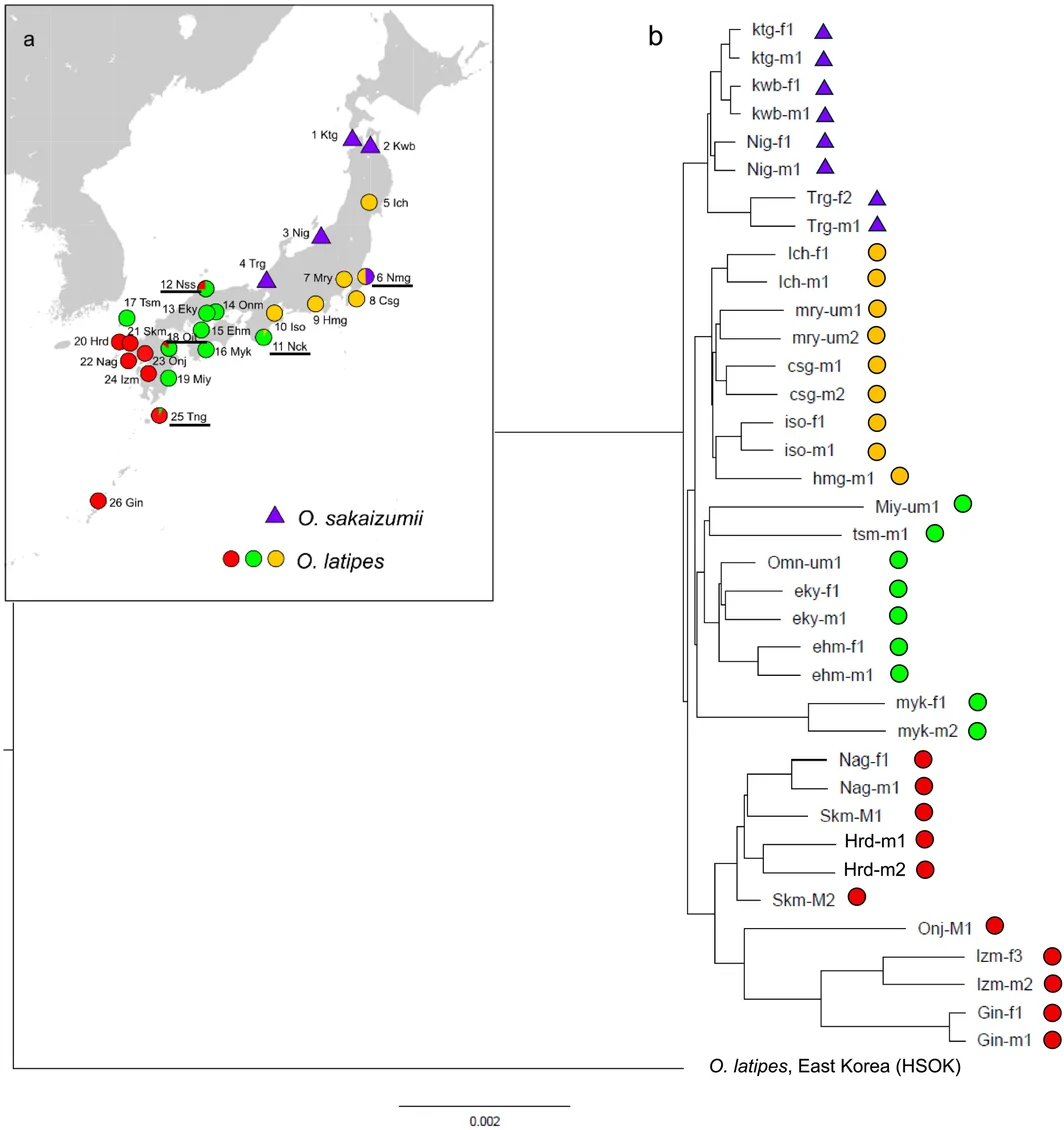
(a) Geographic locations of collection sites. Solid circles and triangles on the map represent *Oryzias sakaizumii* and *Oryzias latipes* , respectively. Colors correspond to the clusters in the ADMIXTURE analysis (*K* = 4). (b) Neighbor‐joining tree based on whole‐genome sequences, excluding individuals with multiple ancestry in the ADMIXTURE analysis (*K* = 4). Excluded sites and individuals (Tng, Nss, Oit, Nck, and Nmg) were represented by site names underlined in the map.

**FIGURE A4:**
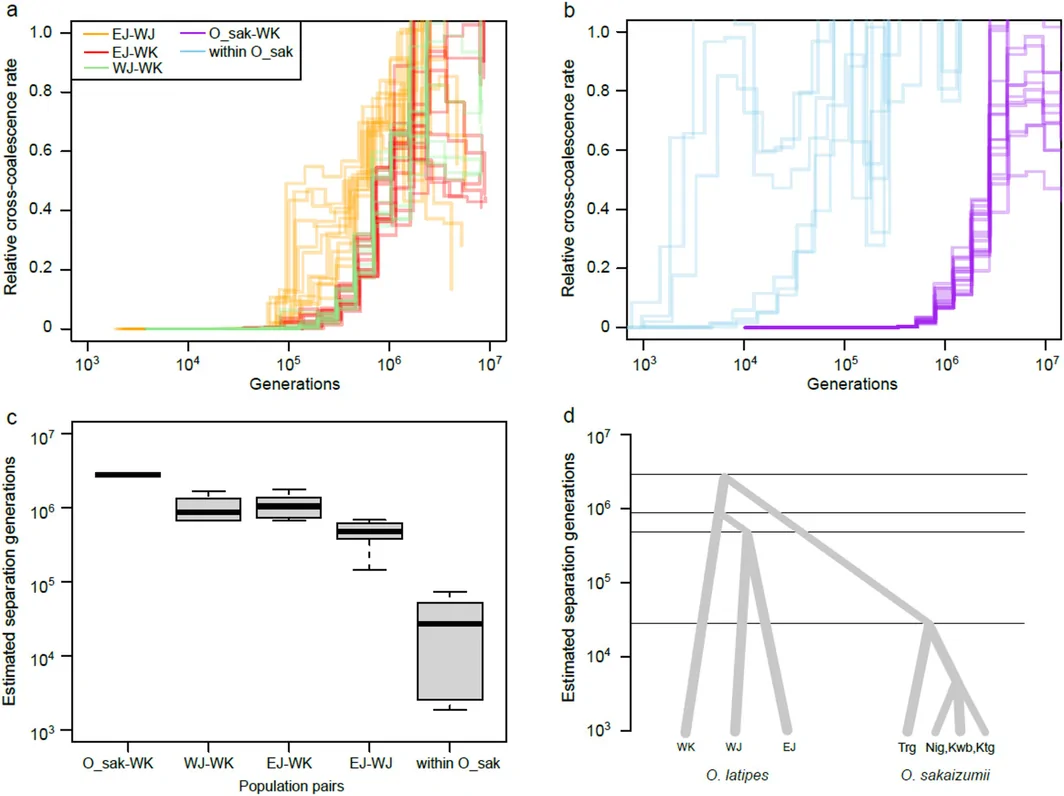
(a, b) Relative cross‐coalescence rates between four populations (a) and between collection sites in *O. sakaizumii* (b), calculated by MSMC2 based on four individuals (eight haplotypes). (c, d) Average divergence times in generations between populations (c) and schematic representation of population divergence (d).

**FIGURE A5:**
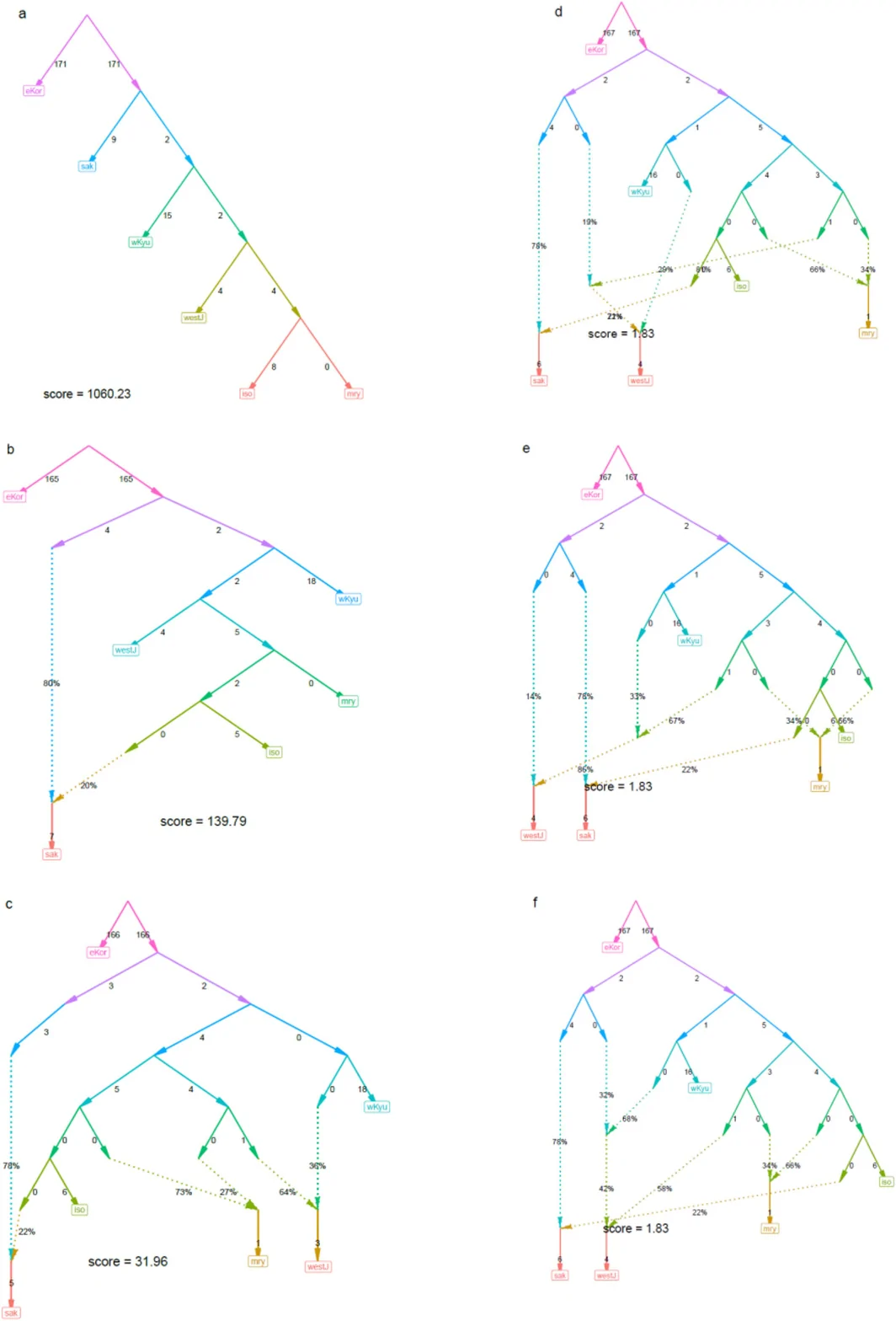
(a–f) Admixture graphs that fit the data. Branch lengths (*F*_*2*_ drift distances) are shown as solid lines, and admixture events are shown as dotted lines with mixture proportions indicated. (e) Introgressive regions identified by a genomic scan of *F*_*d*_ statistics in nonoverlapping 50‐SNV windows.

**FIGURE A6:**
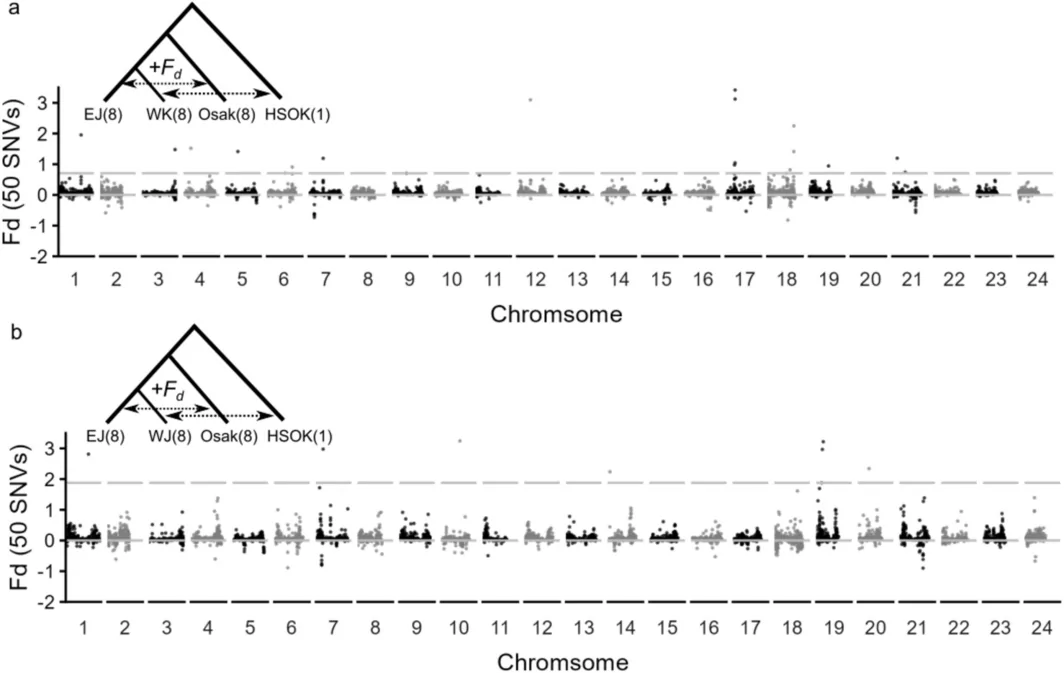
Signature of introgressive regions represented with positive *F*_*d*_ values in the combination of (a) EJ, WK, Osak, HSOK and (b) EJ, WJ, Osak, HSOK. *Oryzias sakaizumii*: Osak; *Oryzias latipes* : Western Kyushu (WK), Western Japan (WJ), and Eastern Japan (EJ).

**FIGURE A7:**
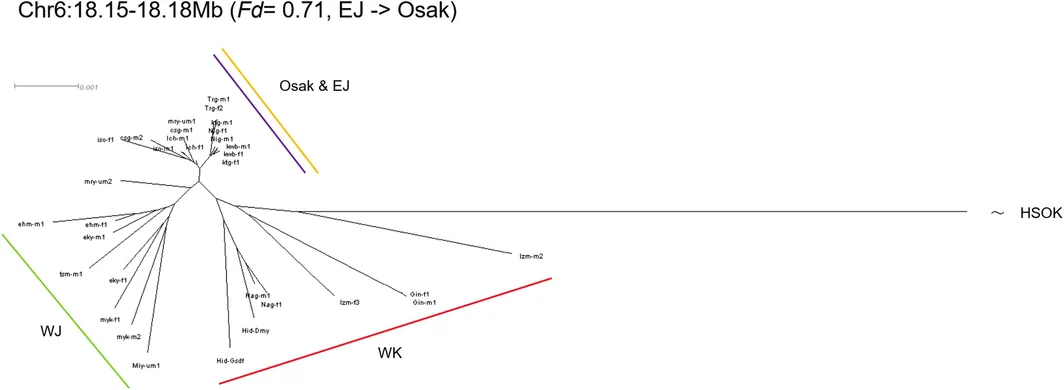
Neighbor joining tree of introgressed locus on chromosome 6. *Oryzias sakaizumii*: Osak; *Oryzias latipes* : Western Kyushu (WK), Western Japan (WJ), and Eastern Japan (EJ).

**FIGURE A8:**
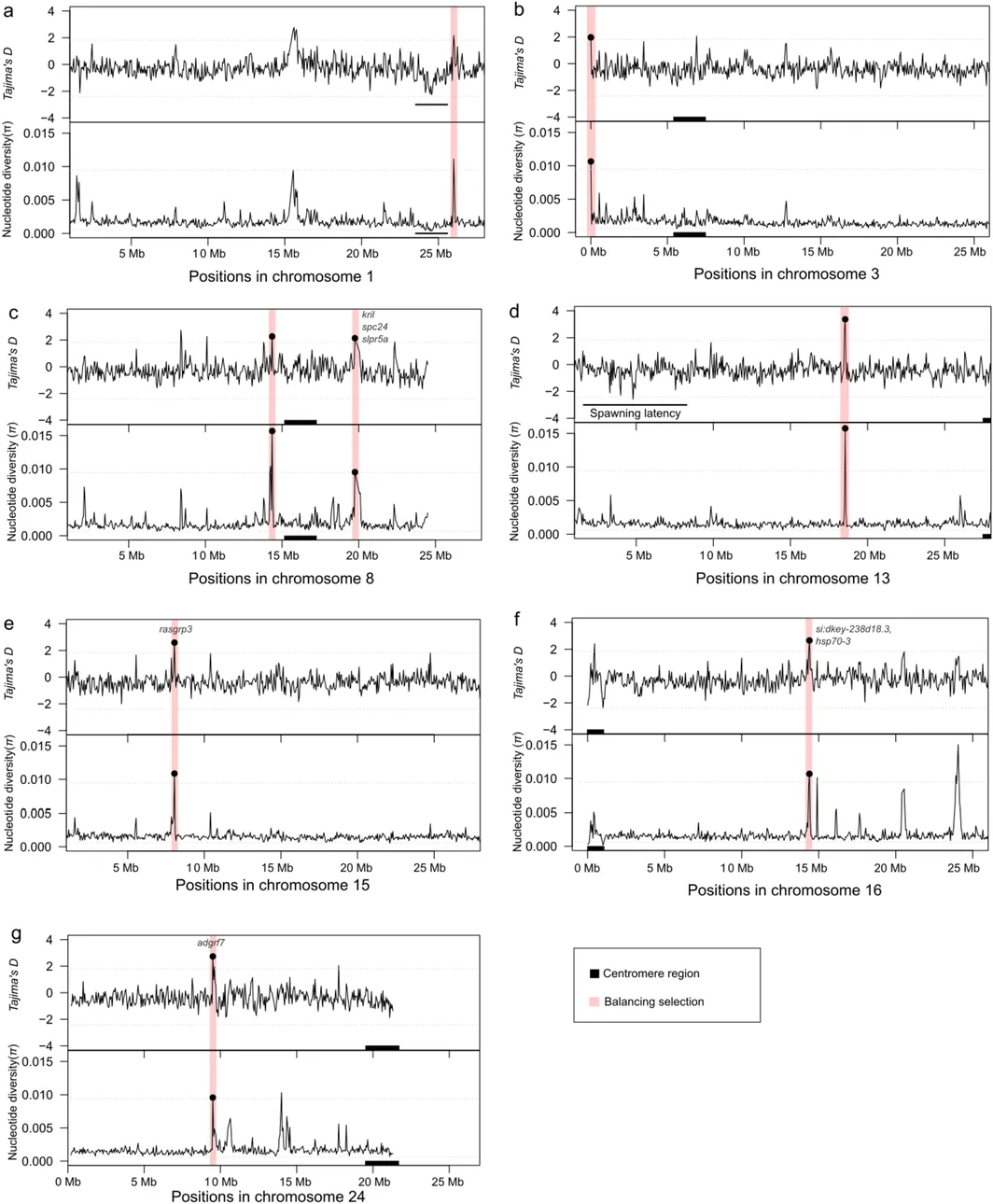
Nucleotide diversity and *Tajima's D* on chromosomes with signature of selection. Centromere regions were inferred by the synteny block positions between *Oryzias latipes* (HdrR strain, ASM223467v1, Ansai et al. 2022, Table A3) and *O. sakaizumii* (HNI strain, ASM223471v1) using ntSynt. Gene positions were based on the reference sequence of *O. sakaizumii*, HNI strain in Ensembl release 109.

**FIGURE A9:**
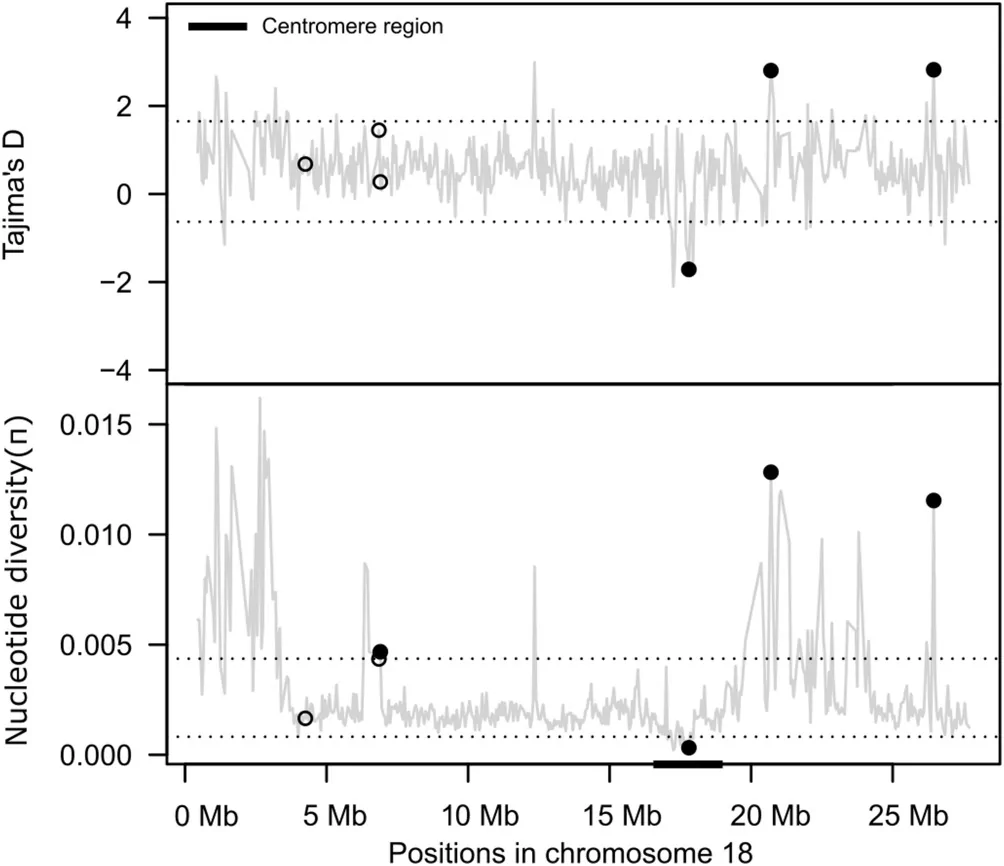
Nucleotide diversity (*π*) and *Tajima's D* on chromosome 18, calculated using individuals from a single collection site (*O. sakaizumii*, Tsuruga: *N* = 16). Dotted lines indicate the upper and lower 2.5% of the empirical genome‐wide distributions. Circles represented outlier windows identified in the dataset of 40 individuals: Filled circles show windows identified as outliers in both datasets (*N* = 16 and *N* = 40), whereas open circles show windows identified as outliers only in the dataset of 40 individuals. Centromere regions were inferred by the synteny block positions between *Oryzias latipes* (HdrR strain, ASM223467v1, Ansai et al. 2022, Table A3) and *O. sakaizumii* (HNI strain, ASM223471v1) using ntSynt.

## Data Availability

Genetic data: Raw sequence reads are deposited in the DDBJ Sequence Read Archive (DRA) database (BioProject: PRJDB20255). Individual genotype data (VCF) are available on Dryad (https://doi.org/10.5061/dryad.xpnvx0kth) Linux scripts for population genetic analysis and custom R scripts are archived in Zenodo (shinFujimoto/popGenomeMedaka: popGenomeMedaka_v1.0.0, DOI: https://doi.org/10.5281/zenodo.21912097).

## Appendix A Additional Figures and Tables

**TABLE A1:** Collection sites of samples of *Oryzias latipes* and *O. sakaizumii*.

Species	Collection sites	Latitude	Longitude	Collection location/strain	Prefecture	Location abbreviation	*N*	Collector	Collection date
*O. sakaizumii*	1	41°0′57″ N	140°24′33″ E	Kitatsugaru	Aomori	Ktg	8	Fujimoto	2019‐08‐29
*O. sakaizumii*	2	40°49′19″ N	140°48′42″ E	Kuwabara	Aomori	Kwb	8	Fujimoto	2019‐08‐28
*O. sakaizumii*	3	37°47′02″ N	138°51′57″ E	Niigata	Niigata	Nig	8	Fujimoto	2019‐08‐25
*O. sakaizumii*	4	35°39′39″ N	136°05′31″ E	Tsuruga	Fukui	Trg	16	Fujimoto	2019‐08‐23
*O. latipes*	5	38°54′35″ N	141°15′39″ E	Ichinoseki	Iwate	Ich	2	Fujimoto	2019‐08‐27
*O. latipes*	6	36°05′15″ N	140°24′58″ E	Namegata	Ibaraki	Nmg	2	Kobayashi	2020‐11‐20
*O. latipes*	7	35°57′14″ N	139°57′15″ E	Moriya	Ibaraki	Mry	2	Murase and Irie	2021‐10‐08
*O. latipes*	8	35°22′43″ N	140°22′22″ E	Choseigun	Chiba	Csg	2	Fujimoto and Murase	2021‐12‐18
*O. latipes*	9	34°48′21″ N	137°40′50″ E	Hamamatsu	Shizuoka	Hmg	1	Kakioka	2021‐10‐10
*O. latipes*	10	34°22′23″ N	136°48′37″ E	Isobe	Mie	Iso	2	Kunishima	2021‐12‐03
*O. latipes*	11	33°35′01″ N	135°54′03″ E	Nachi‐Katsuura	Wakayama	Nck	1	Kunishima	2021‐12‐03
*O. latipes*	12	35°28′00″ N	133°01′17″ E	Nishihamasata	Shimane	Nss	2	Fujimoto and Kobayashi	2020‐08‐01
*O. latipes*	13	34°25′49″ N	133°14′25″ E	Onomichi	Hiroshima	Onm	1	Fujimoto	2019‐06‐23
*O. latipes*	14	34°32′17″ N	133°18′13″ E	Ekiya	Hiroshima	Eky	2	Fujimoto and Kobayashi	2020‐08‐02
*O. latipes*	15	34°01′41″ N	132°57′40″ E	Ehime (Imabari city)	Ehime	Ehm	2	Fujimoto and Kobayashi	2020‐08‐03
*O. latipes*	16	33°24′41″ N	133°17′51″ E	Myoken	Kochi	Myk	2	Fujimoto and Kobayashi	2020‐08‐03
*O. latipes*	17	Not recorded	Not recorded	Tsushima	Nagasaki	Tsm	1	Kobayashi	2022‐04‐16
*O. latipes*	18	Not recorded	Not recorded	Oita	Oita	Oit	1	Kobayashi	2021‐10‐25
*O. latipes*	19	31°58′29″ N	131°28′00″ E	Miyazaki	Miyazaki	Miy	1	Fujimoto	2019‐08‐20
*O. latipes*	20	Not recorded	Not recorded	Hirado	Nagasaki	Hrd	2	Myosho	Not recorded
*O. latipes*	21	Not recorded	Not recorded	Shikamachi	Nagasaki	Skm	2	Myosho	Not recorded
*O. latipes*	22	32°49′40″ N	129°43′21″ E	Nagata	Nagasaki	Nag	8	Fujimoto and Yagi	2019‐06‐03
*O. latipes*	23	32°50′35″ N	130°05′26″ E	Onojima	Nagasaki	Onj	1	Fujimoto and Yagi	2019‐06‐02
*O. latipes*	24	32°05′51″ N	130°17′27″ E	Izumi	Kagoshima	Izm	8	Fujimoto	2019‐08‐19
*O. latipes*	25	30°34′28″ N	131°00′32″ E	Nakatane	Kagoshima	Tng	1	Kobayashi	2019‐05‐31
*O. latipes*	26	26°49′36″ N	128°15′36″ E	Ginama	Okinawa	Gin	8	Fujimoto	2019‐08‐12
*O. latipes*	NA	Not recorded	Not recorded	East Korea, inbred strain HSOK	NA	HSOK	1	Spivakov et al. 2014, DRA000588	NA

**TABLE A2:** Sequence summary of individuals included in this study.

Sample order	Species	Location abbreviation	Collection date	Individual sample ID	Sex phenotype	Sequence group	Total read bases (bp)	Total reads	Coverage^a^	Ancestry	Reference	Phylogeny	Admixture analysis	Genetic diversity within *O. sakaizumii*
1	*O. latipes*	HSOK	NA	HSOK	NA	NA	49,942,933,600	499,429,336	73.702	—	Spivakov et al. 2014, DRA000588	1	1	0
2	*O. latipes*	Gin	2019‐08‐12	Gin‐f1	f	1912JNHX‐0002	25,013,338,750	165,651,250	36.9128	West Kyusyu	This study	1	1	0
3	*O. latipes*	Gin	2019‐08‐12	Gin‐f2	f	1912JNHX‐0002	15,733,678,748	104,196,548	23.2186	West Kyusyu	This study	0	0	0
4	*O. latipes*	Gin	2019‐08‐12	Gin‐f3	f	1912JNHX‐0002	17,278,669,072	114,428,272	25.4985	West Kyusyu	This study	0	0	0
5	*O. latipes*	Gin	2019‐08‐12	Gin‐f4	f	2006JNHX‐0074_5	16,346,744,788	108,256,588	24.1233	West Kyusyu	This study	0	0	0
6	*O. latipes*	Gin	2019‐08‐12	Gin‐m1	m	1912JNHX‐0002	19,087,232,614	126,405,514	28.1675	West Kyusyu	This study	1	1	0
7	*O. latipes*	Gin	2019‐08‐12	Gin‐m2	m	1912JNHX‐0002	22,987,138,606	152,232,706	33.9227	West Kyusyu	This study	0	0	0
8	*O. latipes*	Gin	2019‐08‐12	Gin‐m3	m	1912JNHX‐0002	17,012,519,190	112,665,690	25.1058	West Kyusyu	This study	0	0	0
9	*O. latipes*	Gin	2019‐08‐12	Gin‐m4	m	2006JNHX‐0074_5	18,757,381,570	124,221,070	27.6807	West Kyusyu	This study	0	0	0
10	*O. latipes*	Tng	2019‐05‐31	Tng‐f1	f	2006JNHX‐0074_6	20,291,490,532	134,380,732	29.9446	West Kyusyu and West Japan	This study	1	0	0
11	*O. latipes*	Izm	2019‐08‐19	Izm‐f1	f	1912JNHX‐0002	22,440,017,286	148,609,386	33.1153	West Kyusyu	This study	0	0	0
12	*O. latipes*	Izm	2019‐08‐19	Izm‐f2	f	2006JNHX‐0074_4	12,272,389,100	81,274,100	18.1107	West Kyusyu	This study	0	0	0
13	*O. latipes*	Izm	2019‐08‐19	Izm‐f3	f	2006JNHX‐0074_3	22,087,281,588	146,273,388	32.5947	West Kyusyu	This study	1	1	0
14	*O. latipes*	Izm	2019‐08‐19	Izm‐f4	f	2006JNHX‐0074_4	16,817,955,992	111,377,192	24.8187	West Kyusyu	This study	0	0	0
15	*O. latipes*	Izm	2019‐08‐19	Izm‐m1	m	1912JNHX‐0002	17,906,842,058	118,588,358	26.4256	West Kyusyu	This study	0	0	0
16	*O. latipes*	Izm	2019‐08‐19	Izm‐m2	m	2006JNHX‐0074_4	27,056,310,464	179,180,864	39.9277	West Kyusyu	This study	1	1	0
17	*O. latipes*	Izm	2019‐08‐19	Izm‐m3	m	2006JNHX‐0074_4	22,339,482,090	147,943,590	32.9669	West Kyusyu	This study	0	0	0
18	*O. latipes*	Izm	2019‐08‐19	Izm‐m4	m	2006JNHX‐0074_4	12,631,788,428	83,654,228	18.641	West Kyusyu	This study	0	0	0
19	*O. latipes*	Miy	2019‐08‐20	Miy‐um1	Inmature	2006JNHX‐0074_6	17,315,201,408	114,670,208	25.5525	West Japan	This study	1	1	0
20	*O. latipes*	Onj	2019‐06‐02	Onj‐m1	m	1908JNHX‐0018	31,586,498,912	209,182,112	46.613	West Kyusyu	This study	1	0	0
21	*O. latipes*	Nag	2019‐06‐03	Nag‐f1	f	JN00001693	15,847,637,844	104,951,244	23.3867	West Kyusyu	This study	1	1	0
22	*O. latipes*	Nag	2019‐06‐03	Nag‐f2	f	JN00001693	20,315,442,454	134,539,354	29.98	West Kyusyu	This study	0	0	0
23	*O. latipes*	Nag	2019‐06‐03	Nag‐f3	f	2006JNHX‐0074_3	17,172,447,216	113,724,816	25.3418	West Kyusyu	This study	0	0	0
24	*O. latipes*	Nag	2019‐06‐03	Nag‐f4	f	JN00001693	14,777,716,170	97,865,670	21.8078	West Kyusyu	This study	0	0	0
25	*O. latipes*	Nag	2019‐06‐03	Nag‐m1	m	2006JNHX‐0074_6	16,346,772,270	108,256,770	24.1233	West Kyusyu	This study	1	1	0
26	*O. latipes*	Nag	2019‐06‐03	Nag‐m10	m	JN00001693	28,420,665,830	188,216,330	41.9411	West Kyusyu	This study	0	0	0
27	*O. latipes*	Nag	2019‐06‐03	Nag‐m2	m	JN00001693	12,833,223,636	84,988,236	18.9383	West Kyusyu	This study	0	0	0
28	*O. latipes*	Nag	2019‐06‐03	Nag‐m7	m	JN00001693	12,717,111,280	84,219,280	18.7669	West Kyusyu	This study	0	0	0
29	*O. latipes*	Hrd	Not recorded	Hrd‐m1	m	1908JNHX‐0018	20,312,200,182	134,517,882	29.9752	West Kyusyu	This study	1	1	0
30	*O. latipes*	Hrd	Not recorded	Hrd‐m2	m	1908JNHX‐0018	20,634,304,624	136,651,024	30.4505	West Kyusyu	This study	1	1	0
31	*O. latipes*	Skm	Not recorded	Skm‐m1	m	1908JNHX‐0018	21,004,234,088	139,100,888	30.9965	West Kyusyu	This study	1	0	0
32	*O. latipes*	Skm	Not recorded	Skm‐m2	m	1908JNHX‐0018	23,526,182,332	155,802,532	34.7182	West Kyusyu	This study	1	0	0
33	*O. latipes*	Oit	2021‐10‐25	Oit‐m1	m	JN00006698	22,851,095,156	151,331,756	33.7219	West Japan and West Kyusyu	This study	1	0	0
34	*O. latipes*	Tsm	2022‐04‐16	Tsm‐m1	m	JN00006698	20,889,692,132	138,342,332	30.8274	West Japan	This study	1	1	0
35	*O. latipes*	Ehm	2020‐08‐03	Ehm‐f1	f	JN00001694	27,823,851,618	184,263,918	41.0603	West Japan	This study	1	1	0
36	*O. latipes*	Ehm	2020‐08‐03	Ehm‐m1	m	JN00001694	27,165,213,174	179,902,074	40.0884	West Japan	This study	1	1	0
37	*O. latipes*	Myk	2020‐08‐03	Myk‐f1	f	JN00001694	21,866,881,988	144,813,788	32.2695	West Japan	This study	1	1	0
38	*O. latipes*	Myk	2020‐08‐03	Myk‐m2	m	JN00001694	21,544,127,944	142,676,344	31.7932	West Japan	This study	1	1	0
39	*O. latipes*	Eky	2020‐08‐02	Eky‐f1	f	JN00001694	26,611,809,046	176,237,146	39.2717	West Japan	This study	1	1	0
40	*O. latipes*	Eky	2020‐08‐02	Eky‐m1	m	JN00001694	19,500,820,708	129,144,508	28.7778	West Japan	This study	1	1	0
41	*O. latipes*	Onm	2019‐06‐23	Onm‐um1	Inmature	2006JNHX‐0074_6	14,433,882,828	95,588,628	21.3004	West Japan	This study	1	0	0
42	*O. latipes*	Nss	2020‐08‐01	Nss‐f11	f	JN00001694	31,105,486,600	205,996,600	45.9031	West Japan and Wes Kyusyu	This study	1	0	0
43	*O. latipes*	Nss	2020‐08‐01	Nss‐m11	m	JN00001694	25,320,847,834	167,687,734	37.3666	West Japan and Wes Kyusyu	This study	1	0	0
44	*O. latipes*	Nck	2021‐12‐03	Nck‐m1	m	JN00006698	21,620,551,762	143,182,462	31.906	West Japan and East Japan	This study	1	0	0
45	*O. latipes*	Iso	2021‐12‐03	Iso‐f1	f	JN00006698	23,590,527,962	156,228,662	34.8131	East Japan	This study	1	1	0
46	*O. latipes*	Iso	2021‐12‐03	Iso‐m1	m	JN00006698	25,649,638,556	169,865,156	37.8518	East Japan	This study	1	1	0
47	*O. latipes*	Hmg	2021‐10‐10	Hmg‐m1	m	JN00006698	21,473,467,192	142,208,392	31.6889	East Japan	This study	1	0	0
48	*O. latipes*	Mry	2021‐10‐08	Mry‐um1	Inmature	JN00006698	20,323,539,980	134,592,980	29.9919	East Japan	This study	1	1	0
49	*O. latipes*	Mry	2021‐10‐08	Mry‐um2	Inmature	JN00006698	19,855,304,684	131,492,084	29.301	East Japan	This study	1	1	0
50	*O. latipes*	Nmg	2020‐11‐20	Nmg‐um1	Inmature	JN00001694	25,992,037,096	172,132,696	38.3571	East Japan and Osak	This study	1	0	0
51	*O. latipes*	Nmg	2020‐11‐20	Nmg‐m1	m	JN00001694	31,511,342,286	208,684,386	46.502	East Japan and Osak	This study	1	0	0
52	*O. latipes*	Csg	2021‐12‐18	Csg‐m1	m	JN00006698	17,864,246,770	118,306,270	26.3627	East Japan	This study	1	1	0
53	*O. latipes*	Csg	2021‐12‐18	Csg‐m2	m	JN00006698	22,364,110,492	148,106,692	33.0033	East Japan	This study	1	1	0
54	*O. latipes*	Ich	2019‐08‐27	Ich‐f1	f	2006JNHX‐0074_6	18,638,264,616	123,432,216	27.5049	East Japan	This study	1	1	0
55	*O. latipes*	Ich	2019‐08‐27	Ich‐m1	m	2006JNHX‐0074_6	15,560,677,746	103,050,846	22.9633	East Japan	This study	1	1	0
56	*O. sakaizumii*	Trg	2019‐08‐23	Trg‐f2	f	1912JNHX‐0002	22,049,368,810	146,022,310	32.5388	Osak	This study	1	1	1
57	*O. sakaizumii*	Trg	2019‐08‐23	Trg‐f3	f	2006JNHX‐0074_1	16,901,027,434	111,927,334	24.9413	Osak	This study	0	0	1
58	*O. sakaizumii*	Trg	2019‐08‐23	Trg‐f4	f	2006JNHX‐0074_1	27,352,890,470	181,144,970	40.3653	Osak	This study	0	0	1
59	*O. sakaizumii*	Trg	2019‐08‐23	Trg‐f5	f	2006JNHX‐0074_5	20,466,888,508	135,542,308	30.2035	Osak	This study	0	0	1
60	*O. sakaizumii*	Trg	2019‐08‐23	Trg‐f6	f	JN00006698	28,273,833,128	187,243,928	41.7244	Osak	This study	0	0	1
61	*O. sakaizumii*	Trg	2019‐08‐23	Trg‐f7	f	JN00006698	22,509,353,768	149,068,568	33.2176	Osak	This study	0	0	1
62	*O. sakaizumii*	Trg	2019‐08‐23	Trg‐f8	f	JN00006698	22,030,789,166	145,899,266	32.5114	Osak	This study	0	0	1
63	*O. sakaizumii*	Trg	2019‐08‐23	Trg‐f9	f	JN00006698	21,013,384,990	139,161,490	31.01	Osak	This study	0	0	1
64	*O. sakaizumii*	Trg	2019‐08‐23	Trg‐m1	m	1912JNHX‐0002	18,571,549,494	122,990,394	27.4065	Osak	This study	1	1	1
65	*O. sakaizumii*	Trg	2019‐08‐23	Trg‐m2	m	2006JNHX‐0074_1	21,361,742,594	141,468,494	31.524	Osak	This study	0	0	1
66	*O. sakaizumii*	Trg	2019‐08‐23	Trg‐m3	m	2006JNHX‐0074_1	15,758,982,724	104,364,124	23.2559	Osak	This study	0	0	1
67	*O. sakaizumii*	Trg	2019‐08‐23	Trg‐m4	m	2006JNHX‐0074_1	21,752,118,062	144,053,762	32.1001	Osak	This study	0	0	1
68	*O. sakaizumii*	Trg	2019‐08‐23	Trg‐m5	m	JN00006698	18,079,531,094	119,731,994	26.6804	Osak	This study	0	0	1
69	*O. sakaizumii*	Trg	2019‐08‐23	Trg‐m6	m	JN00006698	17,508,656,266	115,951,366	25.8379	Osak	This study	0	0	1
70	*O. sakaizumii*	Trg	2019‐08‐23	Trg‐m7	m	JN00006698	23,328,190,830	154,491,330	34.426	Osak	This study	0	0	1
71	*O. sakaizumii*	Trg	2019‐08‐23	Trg‐m8	m	JN00006698	25,755,548,446	170,566,546	38.0081	Osak	This study	0	0	1
72	*O. sakaizumii*	Nig	2019‐08‐25	Nig‐f1	f	1912JNHX‐0002	17,507,547,322	115,944,022	25.8363	Osak	This study	1	1	1
73	*O. sakaizumii*	Nig	2019‐08‐25	Nig‐f2	f	2006JNHX‐0074_4	11,661,000,972	77,225,172	17.2084	Osak	This study	0	0	1
74	*O. sakaizumii*	Nig	2019‐08‐25	Nig‐f3	f	2006JNHX‐0074_2	24,200,024,966	160,265,066	35.7126	Osak	This study	0	0	1
75	*O. sakaizumii*	Nig	2019‐08‐25	Nig‐f4	f	2006JNHX‐0074_2	21,343,847,584	141,349,984	31.4976	Osak	This study	0	0	1
76	*O. sakaizumii*	Nig	2019‐08‐25	Nig‐m1	m	1912JNHX‐0002	18,307,498,814	121,241,714	27.0168	Osak	This study	1	1	1
77	*O. sakaizumii*	Nig	2019‐08‐25	Nig‐m2	m	2006JNHX‐0074_2	21,206,865,820	140,442,820	31.2955	Osak	This study	0	0	1
78	*O. sakaizumii*	Nig	2019‐08‐25	Nig‐m6	m	2006JNHX‐0074_2	23,202,744,258	153,660,558	34.2409	Osak	This study	0	0	1
79	*O. sakaizumii*	Nig	2019‐08‐25	Nig‐m7	m	2006JNHX‐0074_2	20,822,676,822	137,898,522	30.7285	Osak	This study	0	0	1
80	*O. sakaizumii*	Kwb	2019‐08‐28	Kwb‐f1	f	2001JNHX‐0113	24,141,348,178	159,876,478	35.626	Osak	This study	1	1	1
81	*O. sakaizumii*	Kwb	2019‐08‐28	Kwb‐f2	f	2001JNHX‐0113	22,644,873,852	149,966,052	33.4176	Osak	This study	0	0	1
82	*O. sakaizumii*	Kwb	2019‐08‐28	Kwb‐f3	f	2006JNHX‐0074_3	20,144,018,496	133,404,096	29.727	Osak	This study	0	0	1
83	*O. sakaizumii*	Kwb	2019‐08‐28	Kwb‐f4	f	2006JNHX‐0074_3	15,778,345,152	104,492,352	23.2845	Osak	This study	0	0	1
84	*O. sakaizumii*	Kwb	2019‐08‐28	Kwb‐m1	m	2001JNHX‐0113	27,618,758,586	182,905,686	40.7577	Osak	This study	1	1	1
85	*O. sakaizumii*	Kwb	2019‐08‐28	Kwb‐m2	m	2001JNHX‐0113	19,922,237,246	131,935,346	29.3997	Osak	This study	0	0	1
86	*O. sakaizumii*	Kwb	2019‐08‐28	Kwb‐m3	m	2006JNHX‐0074_3	12,405,199,036	82,153,636	18.3067	Osak	This study	0	0	1
87	*O. sakaizumii*	Kwb	2019‐08‐28	Kwb‐m4	m	2006JNHX‐0074_3	12,961,542,228	85,838,028	19.1277	Osak	This study	0	0	1
88	*O. sakaizumii*	Ktg	2019‐08‐29	Ktg‐f1	f	2006JNHX‐0074_5	24,151,175,862	159,941,562	35.6405	Osak	This study	1	1	1
89	*O. sakaizumii*	Ktg	2019‐08‐29	Ktg‐f3	f	JN00001693	15,527,773,940	102,832,940	22.9147	Osak	This study	0	0	1
90	*O. sakaizumii*	Ktg	2019‐08‐29	Ktg‐f8	f	JN00001693	19,588,355,408	129,724,208	28.907	Osak	This study	0	0	1
91	*O. sakaizumii*	Ktg	2019‐08‐29	Ktg‐f9	f	JN00001693	23,275,024,938	154,139,238	34.3475	Osak	This study	0	0	1
92	*O. sakaizumii*	Ktg	2019‐08‐29	Ktg‐m1	m	2006JNHX‐0074_5	18,878,899,424	125,025,824	27.86	Osak	This study	1	1	1
93	*O. sakaizumii*	Ktg	2019‐08‐29	Ktg‐m3	m	JN00001693	16,771,226,626	111,067,726	24.7497	Osak	This study	0	0	1
94	*O. sakaizumii*	Ktg	2019‐08‐29	Ktg‐m7	m	JN00001693	14,536,999,218	96,271,518	21.4526	Osak	This study	0	0	1
95	*O. sakaizumii*	Ktg	2019‐08‐29	Ktg‐m8	m	JN00001693	17,320,192,260	114,703,260	25.5598	Osak	This study	0	0	1

^a^ Coverage were calculated based on the reference sequence of HNI strain (ASM223471v1: 677633405 bp).

**TABLE A3:** Inferred position of centromere region in *O. sakaizumii*, HNI reference genome sequence (ASM223471v1).

*Oryzias sakaizumii* (HNI strain, ASM223471v1)		*Oryzias latipes* (HdrR strain, ASM223467v1)	
Chromosome	Centromere repeat detection^a^	Centromere position (Mb)^b^	Inferred position in HNI (Mb)^c^
1	Not detected	25.96–26.02	24.53–24.58
2	Detected	12.03–12.04	9.80–9.82
3	Not detected	7.36–7.40	6.38–6.48
4	Not detected	0–0.01	0–0.04
5	Detected	1.23–1.24	0.3–3.42
6	Detected	0–0.01	—
7	Detected	Not detected	—
8	Not detected	17.02–17.08	16.19–16.21
9	Not detected	21.69–21.71	20.40–20.58
10	Detected	6.46–6.47	5.53–5.55
11	Detected	28.20–28.21	25.13–25.53
12	Detected	21.25–21.26	19.56–19.60
13	Not detected	33.79–33.81	29.85 or more
14	Not detected	0–0.03	0–0.01
15	Not detected	Not detected	—
16	Not detected	0–0.01	0–0.0008
17	Not detected	Not detected	—
18	Not detected	19.72–19.75	17.71–18.07
19	Detected	16.46–16.59	14.58–14.95
20	Not detected	6.05–6.16	3.92–6.23
21	Detected	2.33–2.39	2.26–2.27
21	Not detected	14.52–14.53	13.55–13.56
22	Not detected	28.83–28.98	27.16 or more
23	Not detected	9.87–9.88	9.12–9.21
24	Not detected	22.32–22.33	20.54–20.67

*Note:* Physical positions were inferred based on the synteny block positions of HNI and HdrR reference sequence using ntSynt.

^a^ Centromere repeat detection of HNI strain was performed by Ichikawa et al. 2017, table S12.

^b^ Centromeric position of HdrR strain was retrieved by Ansai et al. 2022, Table 1.

^c^ Poisitions including the centromere of HdrR strain were inferred based on the synteny block positions of HdrR and HNI reference sequence using ntSynt.

**TABLE A4:** Cross‐validation (CV) errors of ADMIXTURE for *K* = 1–10.

*K*	CV error
1	0.47102
2	0.43371
3	0.42222
4	0.40254
5	0.41262
6	0.41168
7	0.43162
8	0.48536
9	0.46398
10	0.49531

**TABLE A5:** *Patterson's D* statistics for five population combinations, *D* (X1, X2; Osak, HSOK), where X1 and X2 are WK, WJ, or EJ.

Population 1	Population 2	Population 3	Population 4	Estimate (*Patterson's D*)	Standard error	*Z*‐value	*p*	nSNPs
EJ	WJ	WK	Osak	−0.37	0.02	−24.11	1.9548E−128	10287244
WJ	WK	Osak	HSOK	0.16	0.01	15.13	1.05E−51	10287244
EJ	WK	Osak	HSOK	0.45	0.01	33.27	9.88E−243	10287244
EJ	WJ	Osak	HSOK	0.36	0.02	23.26	1.18E−119	10287244
EJ	WJ	WK	HSOK	−0.01	0.01	−0.94	0.345173785	10287244
EJ	WK	WJ	HSOK	0.34	0.01	38.91	0.00E+00	10287244
WJ	WK	EJ	HSOK	0.35	0.01	33.84	4.54E−251	10287244
EJ	WJ	Osak(Ktg)	HSOK	0.36	0.02	22.93	2.22E−116	10287244
EJ	WJ	Osak(Kwb)	HSOK	0.36	0.02	23.05	1.36E−117	10287244
EJ	WJ	Osak(Nig)	HSOK	0.36	0.02	23.46	1.06E−121	10287244
EJ	WJ	Osak(Trg)	HSOK	0.35	0.01	23.35	1.45E−120	10287244
EJ(Iso)	WJ	Osak	HSOK	0.42	0.02	27.03	6.62E−161	10287244
EJ(Csg)	WJ	Osak	HSOK	0.33	0.02	16.81	2.07E−63	10287244
EJ(Mry)	WJ	Osak	HSOK	0.29	0.02	11.84	2.52E−32	10287244
EJ(Ich)	WJ	Osak	HSOK	0.39	0.02	18.23	3.19E−74	10287244
EJ(Iso)	EJ(Csg)	Osak	HSOK	0.16	0.03	5.97	2.38E−09	10287244
EJ(Iso)	EJ(Mry)	Osak	HSOK	0.21	0.03	7.84	4.42E−15	10287244
EJ(Iso)	EJ(Ich)	Osak	HSOK	−0.03	0.03	−0.90	0.366503726	10287244
EJ(Iso)	EJ(Csg)	WJ	HSOK	−0.11	0.02	−6.48	8.93E−11	10287244
EJ(Iso)	EJ(Mry)	WJ	HSOK	−0.16	0.02	−6.94	3.86E−12	10287244
EJ(Iso)	EJ(Ich)	WJ	HSOK	−0.02	0.02	−0.72	0.471462747	10287244
EJ	WJ(Ehm)	Osak	HSOK	0.25	0.02	14.53	8.05255E−48	10287244
EJ	WJ(Eky)	Osak	HSOK	0.15	0.02	8.39	4.69471E−17	10287244
EJ	WJ(Miy)	Osak	HSOK	0.44	0.02	27.90	2.4745E−171	10287244
EJ	WJ(Myk)	Osak	HSOK	0.50	0.02	30.67	1.5344E−206	10287244
EJ	WJ(Tsm)	Osak	HSOK	0.39	0.02	24.52	8.8033E−133	10287244
EJ	WK(Hid)	Osak	HSOK	0.33	0.01	22.19	4.3023E−109	10287244
EJ	WK(Nag)	Osak	HSOK	0.27	0.02	17.48	2.20154E−68	10287244
EJ	WK(Izm)	Osak	HSOK	0.55	0.01	44.99	0	10287244
EJ	WK(Gin)	Osak	HSOK	0.54	0.01	42.01	0	10287244
WK	WJ(Eky)	Osak	HSOK	0.36	0.01	31.27	1.26E−214	10287244
WK	WJ(Ehm)	Osak	HSOK	0.27	0.01	22.60	4.64E−113	10287244
WK	WJ(Tsm)	Osak	HSOK	0.13	0.01	11.02	3.04E−28	10287244
WK	WJ(Miy)	Osak	HSOK	0.07	0.01	6.11	1.00E−09	10287244
WK	WJ(Myk)	Osak	HSOK	−0.03	0.02	−1.84	0.065639404	10287244
WK(Gin)	WJ	Osak	HSOK	0.29	0.01	28.00	1.86E−172	10287244
WK(Izm)	WJ	Osak	HSOK	0.30	0.01	31.43	8.42E−217	10287244
WK(Hid)	WJ	Osak	HSOK	0.01	0.01	0.49	0.622708591	10287244
WK(Nag)	WJ	Osak	HSOK	−0.06	0.01	−4.43	9.38E‐06	10287244

**TABLE A6:** Introgressed chromosomal regions in *O. sakaizumii* estimated by outliers *F*_*d*_ (EJ, WK; Osak, HSOK) and haplotype‐based signature of selective sweep shown by integrated haplotype score.

Candidate regions by introgression from *O. latipes*				Integrated haplotype score (iHS)		
Chr	Start	End (bp)	*F*_*d*_	Outlier SNPs	Max |iHS|	Max |iHS| position
1	22493407	22499856	1.953	0	0.52	22498558
3	32991373	33072702	1.478	0	1.37	33005085
4	7285700	7298888	1.519	0	2.92	7290892
5	12632712	12648282	1.414	0	1.75	12638544
6	18152129	18181016	0.711	0	1.56	18168615
6	25136922	25166290	0.909	0	1.90	25152500
7	14473429	14506964	1.191	0	0.42	14481141
12	13372685	13382849	3.096	0	0.60	13376998
17	8984655	8994373	0.979	0	1.74	8989727
17	9507694	9556459	1.045	0	1.12	9551802
17	9556463	9564269	3.418	0	1.28	9557969
17	9564271	9572263	3.124	0	1.37	9571208
18	22685878	22722587	0.820	0	0.82	22697014
18	26420749	26430877	1.415	0	0.73	26425572
18	26430901	26437927	2.247	0	0.38	26436540
19	19488436	19509024	0.945	0	NA	NA
21	4940364	4966096	1.196	0	1.38	4958333
21	12782534	12934471	0.739^a^	0	0.25	12912141

^a^ Centromere region were inferred based on the synteny block positions of HdrR and HNI references using ntSynt (Table A3), and overlap determined within 1 Mb of the centromere regions.

**TABLE A7:** Gene symbols in candidate regions of introgression and/or selection signatures.

*Oryzias sakaizumii* (HNI strain, GCA_002234715.1_ASM223471v1_genomic.fna)						*Oryzias latipes* (HdrR strain, GCA_002234675.1_ASM223467v1_genomic.fna)				
Chromosome	Gene start (bp)	Gene end (bp)	Ensembl gene ID	Population genetic statistics outlier	Near centromere region^a^	Gene start (bp)	Gene end (bp)	Ensembl gene ID	Gene symbol	Description
3	32928463	32996620	ENSORLG00020016716	*Fd* (EJ→sak)		34487987	34509545	ENSORLG00000024842	NA	Adhesion g‐protein coupled receptor g2‐like
4	7281567	7296169	ENSORLG00020003830	*Fd* (EJ→sak)		7193740	7209643	ENSORLG00000030635	fgf22	Fibroblast growth factor 22
5	12634038	12635748	ENSORLG00020015291	*Fd* (EJ→sak)		NA	NA	NA	NA	NA
6	18176782	18185329	ENSORLG00020001574	*Fd* (EJ→sak)	NA	20614467	20625549	ENSORLG00000010223	cpt1b	Carnitine o‐palmitoyltransferase 1
6	18163019	18171617	ENSORLG00020001591	*Fd* (EJ→sak)	NA	20600543	20609294	ENSORLG00000010160	chkb	Choline/ethanolamine kinase
6	18153585	18159565	ENSORLG00020001614	*Fd* (EJ→sak)	NA	20587306	20593157	ENSORLG00000010086	NA	Choriogenin hminor
6	18150654	18153989	ENSORLG00020001643	*Fd* (EJ→sak)	NA	20583312	20587549	ENSORLG00000010134	1‐sf	L‐sf precursor
6	25136411	25169659	ENSORLG00020013211	*Fd* (EJ→sak)	NA	27622633	27657264	ENSORLG00000025818	sntb2	Syntrophin beta 2
7	14506352	14517807	ENSORLG00020001807	*Fd* (EJ→sak)	NA	14693565	14701413	ENSORLG00000009232	samhd1	Sam and hd domain containing deoxynucleoside triphosphate triphosphohydrolase 1
7	14477117	14491175	ENSORLG00020003150	*Fd* (EJ→sak)	NA	14682432	14690342	ENSORLG00000021917	NA	Deoxynucleoside triphosphate triphosphohydrolase samhd1‐like
12	13372047	13387650	ENSORLG00020015932	*Fd* (EJ→sak)		14132954	14147904	ENSORLG00000008971	bmp1b	Bone morphogenetic protein 1
17	8980285	8987739	ENSORLG00020001087	*Fd* (EJ→sak)		10860808	10864794	ENSORLG00000006920	tmub1	Transmembrane and ubiquitin like domain containing 1
17	8990365	8998083	ENSORLG00020018349	*Fd* (EJ→sak)		10868733	10876346	ENSORLG00000028093	ubtfl	Nucleolar transcription factor 1‐a‐like
17	9551216	9557192	ENSORLG00020016541	*Fd* (EJ→sak)		11544668	11553078	ENSORLG00000029713	NA	Myb‐related protein b
17	9535548	9545053	ENSORLG00020016553	*Fd* (EJ→sak)		11479689	11524867	ENSORLG00000025244	NA	NA
17	9519651	9532661	ENSORLG00020016562	*Fd* (EJ→sak)		NA	NA	NA	NA	NA
17	9563131	9579564	ENSORLG00020016505	*Fd* (EJ→sak)		11558449	11575132	ENSORLG00000007142	WDR33	Wd repeat domain 33
18	22696226	22697656	ENSORLG00020015138	*Fd* (EJ→sak)		26159469	26166387	ENSORLG00000010357	si:dkey‐6n21.13	Si:dkey‐6n21.13
18	26410605	26435828	ENSORLG00020011567	*Fd* (EJ→sak)		29454502	29468096	ENSORLG00000012050	slc1a1	Solute carrier family 1 member 1
19	19478319	19513799	ENSORLG00020019337	*Fd* (EJ→sak)		22061938	22088487	ENSORLG00000022172	rnf213b	*Danio rerio* ring finger protein 213b (rnf213b)
21	4945659	4972717	ENSORLG00020015956	*Fd* (EJ→sak)		5283813	5309108	ENSORLG00000010301	fam160a2	Fts and hook‐interacting protein homolog
21	4942252	4949740	ENSORLG00020015979	*Fd* (EJ→sak)		5282992	5287852	ENSORLG00000024354	NA	Uncharacterized loc105356746
21	4926735	4941655	ENSORLG00020015998	*Fd* (EJ→sak)		5254807	5274117	ENSORLG00000010261	ankrd50l	Ankyrin repeat domain 50
21	12923822	12937469	ENSORLG00020006773	*Fd* (EJ→sak)	^a^	13379024	13390165	ENSORLG00000013916	NA	NA
21	12887091	12888199	ENSORLG00020006780	*Fd* (EJ→sak)	^a^	13341848	13344954	ENSORLG00000024077	NA	NA
21	12886645	12924928	ENSORLG00020006787	*Fd* (EJ→sak)	^a^	13360479	13363753	ENSORLG00000023929	NA	NA
21	12854348	12860770	ENSORLG00020006802	*Fd* (EJ→sak)	^a^	13315099	13316213	ENSORLG00000025608	NA	NA
21	12822243	12823349	ENSORLG00020006815	*Fd* (EJ→sak)	^a^	13269989	13271110	ENSORLG00000028977	NA	NA
21	12800926	12805460	ENSORLG00020006837	*Fd* (EJ→sak)	^a^	NA	NA	NA	NA	NA
21	12795612	12796726	ENSORLG00020006851	*Fd* (EJ→sak)	^a^	13192097	13193210	ENSORLG00000025011	NA	NA
21	12776027	12782859	ENSORLG00020006861	*Fd* (EJ→sak)	^a^	13166510	13170930	ENSORLG00000013895	NA	NA
1	15071100	15081508	ENSORLG00020020644	iHSoutlier_CP020779.1_15078135		15996719	16005986	ENSORLG00000006773	aimp1a	Aminoacyl trna synthetase complex interacting multifunctional protein 1
1	16006402	16018086	ENSORLG00020019104	iHSoutlier_CP020779.1_16006860		16919914	16929976	ENSORLG00000026000	NA	NA
1	16199097	16373068	ENSORLG00020018654	iHSoutlier_CP020779.1_16365897		17200196	17283960	ENSORLG00000007162	bnc2	Zinc finger protein basonuclin‐2
1	21496139	21505509	ENSORLG00020011155	iHSoutlier_CP020779.1_21503728		22478152	22490127	ENSORLG00000019573	NA	Perforin‐1‐like
1	21515845	21539872	ENSORLG00020011080	iHSoutlier_CP020779.1_21528461		22500946	22523817	ENSORLG00000009385	VAV1	Vav guanine nucleotide exchange factor 1
1	29512583	29589758	ENSORLG00020021086	iHSoutlier_CP020779.1_29516512		31614181	31706146	ENSORLG00000014026	tusc3	Tumor suppressor candidate 3
3	17936213	17969860	ENSORLG00020011602	iHSoutlier_CP020781.1_17958199		19408148	19463434	ENSORLG00000006838	tub	Tub bipartite transcription factor
3	24925065	24934438	ENSORLG00020001905	iHSoutlier_CP020781.1_24932449		26467138	26476187	ENSORLG00000012326	NA	Tropomyosin alpha‐1 chain
4	7013827	7017250	ENSORLG00020004168	iHSoutlier_CP020782.1_7022462		6967532	6972860	ENSORLG00000004111	sec22bb	Vesicle‐trafficking protein sec22b‐b
4	7114459	7117274	ENSORLG00020004041	iHSoutlier_CP020782.1_7114508		7054359	7061477	ENSORLG00000029483	atp5f1d	Atp synthase f1 subunit delta
4	7185249	7193933	ENSORLG00020003989	iHSoutlier_CP020782.1_7192745		7104152	7126370	ENSORLG00000004305	RNF126	Ring finger protein 126
4	13435935	13461458	ENSORLG00020015450	iHSoutlier_CP020782.1_13450432		13437538	13461683	ENSORLG00000008156	NA	Atp‐binding cassette sub‐family a member 1
4	14593030	14634361	ENSORLG00020013576	iHSoutlier_CP020782.1_14599910		14580268	14597843	ENSORLG00000008850	ano8b	Anoctamin 8
4	14654569	14659057	ENSORLG00020013411	iHSoutlier_CP020782.1_14658942		14638834	14643343	ENSORLG00000008874	plvapb	Plasmalemma vesicle associated protein
4	14729514	14768172	ENSORLG00020013176	iHSoutlier_CP020782.1_14732376		14715960	14751288	ENSORLG00000008960	csnk1g2b	Casein kinase i
5	19840756	19856793	ENSORLG00020004654	iHSoutlier_CP020783.1_19850805		20102147	20118727	ENSORLG00000010556	cntn2	Contactin 2
7	11798250	11803228	ENSORLG00020008217	iHSoutlier_CP020785.1_11801302	NA	11955783	11960974	ENSORLG00000029544	tmem9	Transmembrane protein 9
7	14314285	14323126	ENSORLG00020003852	iHSoutlier_CP020785.1_14315027	NA	14533811	14538305	ENSORLG00000008949	ddx20	Dead‐box helicase 20
7	14325577	14364791	ENSORLG00020003816	iHSoutlier_CP020785.1_14349066	NA	14544091	14580371	ENSORLG00000009019	NA	Cyclin‐dependent kinase 17
7	14366882	14381762	ENSORLG00020003684	iHSoutlier_CP020785.1_14374385	NA	14583102	14596980	ENSORLG00000009038	NA	Transmembrane prolyl 4‐hydroxylase
7	14431030	14442794	ENSORLG00020003401	iHSoutlier_CP020785.1_14441227	NA	14643361	14654636	ENSORLG00000023101	si:ch211‐117c9.1	Nuak family snf1‐like kinase 1
7	14607104	14610403	ENSORLG00020001640	iHSoutlier_CP020785.1_14607767	NA	NA	NA	NA	NA	NA
7	14998679	15097904	ENSORLG00020001510	iHSoutlier_CP020785.1_15002070	NA	15180520	15288330	ENSORLG00000009521	opn7b	Opsin‐5‐like
7	16825043	16853486	ENSORLG00020020210	iHSoutlier_CP020785.1_16846152	NA	17170369	17192643	ENSORLG00000011039	IFT122	Intraflagellar transport 122
7	19445861	19579291	ENSORLG00020015678	iHSoutlier_CP020785.1_19575673	NA	19884153	20030092	ENSORLG00000013266	NA	Metabotropic glutamate receptor 4
8	10124753	10131404	ENSORLG00020020372	iHSoutlier_CP020786.1_10125176		NA	NA	NA	NA	NA
8	14840699	14881765	ENSORLG00020011786	iHSoutlier_CP020786.1_14853298		15580449	15600989	ENSORLG00000012410	sec14l1	Sec14 like lipid binding 1
8	15060429	15072192	ENSORLG00020011649	iHSoutlier_CP020786.1_15060746		15783170	15794942	ENSORLG00000012497	prpsap1	Phosphoribosyl pyrophosphate synthase‐associated protein 1
8	18737846	18750722	ENSORLG00020006044	iHSoutlier_CP020786.1_18737934		20075542	20088332	ENSORLG00000025948	wbp2nl	Wbp2 n‐terminal like
8	19626639	19639187	ENSORLG00020005017	iHSoutlier_CP020786.1_19631986		21010174	21022789	ENSORLG00000015311	tom1	Target of myb protein 1
8	19774328	19775455	ENSORLG00020004763	iHSoutlier_CP020786.1_19774597		21174934	21176061	ENSORLG00000015375	s1pr5a	Sphingosine 1‐phosphate receptor 1
8	19778186	19781591	ENSORLG00020004759	iHSoutlier_CP020786.1_19779222		21178813	21181954	ENSORLG00000015391	spc24	Spc24 component of ndc80 kinetochore complex
8	19907894	19921916	ENSORLG00020004611	iHSoutlier_CP020786.1_19914577		21374270	21388737	ENSORLG00000015452	smarca4a	Swi/snf related
9	3382632	3482559	ENSORLG00020009592	iHSoutlier_CP020787.1_3481874		3955455	3960041	ENSORLG00000001227	mrps30	Mitochondrial ribosomal protein s30
9	23434708	23450227	ENSORLG00020021419	iHSoutlier_CP020787.1_23434909		24933381	24953491	ENSORLG00000026050	mxd1	Max dimerization protein 1
10	5392858	5395802	ENSORLG00020006311	iHSoutlier_CP020788.1_5393892	^a^	6352109	6353330	ENSORLG00000000389	NA	NA
11	6662622	6670467	ENSORLG00020005711	iHSoutlier_CP020789.1_6664530		22942455	22950288	ENSORLG00000012474	azin1b	Antizyme inhibitor 1
11	17434794	17439165	ENSORLG00020004309	iHSoutlier_CP020789.1_17437439		11379292	11383673	ENSORLG00000005043	hoxa13a	Homeobox protein hox‐a13a
12	6977516	6982039	ENSORLG00020009339	iHSoutlier_CP020790.1_6982001		7764360	7768880	ENSORLG00000003980	surf6	Surfeit 6
12	6982620	6988843	ENSORLG00020009327	iHSoutlier_CP020790.1_6988310		7769462	7776109	ENSORLG00000004002	kyat1	Kynurenine aminotransferase 1
12	7546256	7609145	ENSORLG00020008279	iHSoutlier_CP020790.1_7604803		8319991	8381323	ENSORLG00000004609	itga1	Integrin subunit alpha 1
13	7536606	7615244	ENSORLG00020012816	iHSoutlier_CP020791.1_7538965		8535475	8570199	ENSORLG00000003808	stard13b	Star related lipid transfer domain containing 13
13	10061632	10073475	ENSORLG00020008694	iHSoutlier_CP020791.1_10061825		11080477	11087343	ENSORLG00000005039	SAMD4B	Sterile alpha motif domain containing 4b
14	6410304	6424822	ENSORLG00020019469	iHSoutlier_CP020792.1_6420376		7778437	7791652	ENSORLG00000001788	slc9a6b	Sodium/hydrogen exchanger 6
14	6434626	6442736	ENSORLG00020019369	iHSoutlier_CP020792.1_6437064		7801925	7806853	ENSORLG00000001812	snx12	Sorting nexin 12
14	9674778	9687997	ENSORLG00020011296	iHSoutlier_CP020792.1_9677044		11149399	11158295	ENSORLG00000028524	CHORDC1	Cysteine and histidine rich domain containing 1
14	9987297	9994426	ENSORLG00020011078	iHSoutlier_CP020792.1_9993944		11445383	11453041	ENSORLG00000004658	tpte	Phosphatidylinositol 3
14	10005020	10013509	ENSORLG00020010939	iHSoutlier_CP020792.1_10012776		11460622	11470958	ENSORLG00000004700	vps36	Vacuolar protein sorting 36 homolog
14	10009268	10024855	ENSORLG00020010886	iHSoutlier_CP020792.1_10024107		11471705	11485211	ENSORLG00000004718	proser1	Proline and serine rich 1
14	15103047	15137934	ENSORLG00020021977	iHSoutlier_CP020792.1_15103646		16710270	16740838	ENSORLG00000009520	ar	Androgen receptor beta
14	26060314	26226486	ENSORLG00020008278	iHSoutlier_CP020792.1_26061922		27716667	27870713	ENSORLG00000015249	tmem132e	Transmembrane protein 132e
15	14422710	14485967	ENSORLG00020012841	iHSoutlier_CP020793.1_14423742		15911667	15964863	ENSORLG00000004404	NA	Inactive phospholipase d5
15	21228897	21238362	ENSORLG00020003415	iHSoutlier_CP020793.1_21229439		22775417	22781979	ENSORLG00000007699	pyroxd2	Pyridine nucleotide‐disulfide oxidoreductase domain 2
15	21595691	21615919	ENSORLG00020002803	iHSoutlier_CP020793.1_21613944		23136539	23158652	ENSORLG00000008249	NA	Canalicular multispecific organic anion transporter 1‐like
16	13203848	13263241	ENSORLG00020020675	iHSoutlier_CP020794.1_13258319		13641886	13891708	ENSORLG00000005457	csmd3a	Cub and sushi domain‐containing protein 3
17	5202355	5211583	ENSORLG00020021876	iHSoutlier_CP020795.1_5210445		6569163	6578312	ENSORLG00000005579	NA	Calponin 2
17	8688863	8694107	ENSORLG00020019098	iHSoutlier_CP020795.1_8689397		10298365	10303518	ENSORLG00000006634	cldn15la	Claudin‐15
17	8716983	8729696	ENSORLG00020018945	iHSoutlier_CP020795.1_8726836		10325971	10339092	ENSORLG00000006735	proca	Protein c; inactivator of coagulation factors va and viiia
17	20870758	21028391	ENSORLG00020014582	iHSoutlier_CP020795.1_20896365		23748832	23905477	ENSORLG00000015972	ephb1	Ephrin type‐b receptor 1‐b
18	2540232	2560608	ENSORLG00020020880	iHSoutlier_CP020796.1_2540664		3043219	3060178	ENSORLG00000002735	abcg2a	Atp‐binding cassette sub‐family g member 2‐like
18	16325664	16329137	ENSORLG00020007275	iHSoutlier_CP020796.1_16328790		18100789	18102949	ENSORLG00000024017	NA	NA
19	1209045	1210564	ENSORLG00020008605	iHSoutlier_CP020797.1_1209150		1503997	1510504	ENSORLG00000023838	cybc1	Chromosome 19 c17orf62 homolog
19	1202350	1211420	ENSORLG00020008639	iHSoutlier_CP020797.1_1209150		1498332	1508592	ENSORLG00000019153	hexdc	Hexosaminidase d
19	2659507	2662248	ENSORLG00020006194	iHSoutlier_CP020797.1_2660754		3025017	3027747	ENSORLG00000026120	gb	Hemoglobin subunit beta‐1‐like
19	8388641	8395288	ENSORLG00020009890	iHSoutlier_CP020797.1_8394133		9494182	9503805	ENSORLG00000008132	fam234a	Family with sequence similarity 234 member a
19	9149872	9151821	ENSORLG00020008495	iHSoutlier_CP020797.1_9151754		10255011	10257783	ENSORLG00000027749	NA	Dual specificity phosphatase 13
20	15611235	15619691	ENSORLG00020014726	iHSoutlier_CP020798.1_15613363		17144362	17152357	ENSORLG00000008598	mtfr1	Mitochondrial fission regulator 1
20	18675082	18678208	ENSORLG00020009561	iHSoutlier_CP020798.1_18675919		20251336	20254254	ENSORLG00000011032	NA	Clusterin like 1
21	3765041	3791141	ENSORLG00020018929	iHSoutlier_CP020799.1_3767021		4078511	4096201	ENSORLG00000024138	NA	Interleukin‐1 receptor type 1
21	19542593	19548636	ENSORLG00020022334	iHSoutlier_CP020799.1_19548458		20966694	20972810	ENSORLG00000030380	cd302	Cd302 molecule
21	19550707	19565526	ENSORLG00020022335	iHSoutlier_CP020799.1_19553123		20973608	20989280	ENSORLG00000016818	RBMS1	Rna‐binding motif; single‐stranded‐interacting protein 1
21	19575268	19584523	ENSORLG00020022337	iHSoutlier_CP020799.1_19583315		20998674	21007989	ENSORLG00000029472	tank	Traf family member associated nfkb activator
21	19966461	19979421	ENSORLG00020022517	iHSoutlier_CP020799.1_19969093		21395868	21410177	ENSORLG00000016992	ttc21b	Tetratricopeptide repeat domain 21b
21	20032908	20053089	ENSORLG00020022537	iHSoutlier_CP020799.1_20040713		21470301	21481768	ENSORLG00000017017	xirp2b	Xin Actin binding repeat containing 2
21	20273719	20278746	ENSORLG00020022561	iHSoutlier_CP020799.1_20278215		21700813	21705545	ENSORLG00000017147	bbs5	Bardet‐biedl syndrome 5
22	6298949	6312095	ENSORLG00020000562	iHSoutlier_CP020800.1_6311060		6698376	6707496	ENSORLG00000012797	NEMF	Nuclear export mediator factor
22	6468994	6476185	ENSORLG00020018299	iHSoutlier_CP020800.1_6475623		6884655	6893299	ENSORLG00000028182	BDKRB2	B2 bradykinin receptor
24	8337447	8344700	ENSORLG00020009609	iHSoutlier_CP020802.1_8338597		9209325	9216739	ENSORLG00000014927	smyd2b	Set and mynd domain containing 2
18	17810427	17837539	ENSORLG00020005262	Low *π* & Low *Tajima's D*	^a^	19844477	19872310	ENSORLG00000008345	NA	Rab39b; member ras oncogene family
18	17795684	17802075	ENSORLG00020005273	Low *π* & Low *Tajima's D*	^a^	NA	NA	NA	NA	NA
1	25993858	26065990	ENSORLG00020006440	High *π* & High *Tajima's D*		28065896	28078361	ENSORLG00000023460	NA	NA
3	22000	27056	ENSORLG00020008066	High *π* & High *Tajima's D*		NA	NA	NA	NA	NA
8	14351259	14415918	ENSORLG00020012633	High *π* & High *Tajima's D*		14730469	14771610	ENSORLG00000024471	NA	NA
8	14350593	14351687	ENSORLG00020012660	High *π* & High *Tajima's D*		14646955	14652168	ENSORLG00000024303	NA	NA
8	19794604	19867373	ENSORLG00020004716	High *π* & High *Tajima's D*		21319440	21343047	ENSORLG00000022846	NA	NA
8	19786436	19788695	ENSORLG00020004732	High *π* & High *Tajima's D*		21186862	21189118	ENSORLG00000025827	NA	NA
8	19783417	19783514	ENSORLG00020004734	High *π* & High *Tajima's D*		NA	NA	NA	NA	NA
8	19782584	19785706	ENSORLG00020004750	High *π* & High *Tajima's D*		21182995	21186142	ENSORLG00000015403	kri1	Kri1 homolog
8	19778186	19781591	ENSORLG00020004759	High *π* & High *Tajima's D*		21178813	21181954	ENSORLG00000015391	spc24	Spc24 component of ndc80 kinetochore complex
8	19774328	19775455	ENSORLG00020004763	High *π* & High *Tajima's D*		21174934	21176061	ENSORLG00000015375	s1pr5a	Sphingosine 1‐phosphate receptor 1
10	13798733	13798916	ENSORLG00020014623	High *π* & High *Tajima's D*		NA	NA	NA	NA	NA
10	13796249	13799431	ENSORLG00020014632	High *π* & High *Tajima's D*		15180755	15185605	ENSORLG00000026864	NA	NA
10	13782255	13785081	ENSORLG00020014645	High *π* & High *Tajima's D*		15171740	15179813	ENSORLG00000029341	NA	NA
10	13772014	13778071	ENSORLG00020014667	High *π* & High *Tajima's D*		15167581	15171200	ENSORLG00000024772	NA	Protocadherin alpha‐3‐like
10	13763757	13766796	ENSORLG00020014682	High *π* & High *Tajima's D*		15153493	15161478	ENSORLG00000023815	NA	NA
10	13756467	13762680	ENSORLG00020014691	High *π* & High *Tajima's D*		15149718	15155925	ENSORLG00000029952	NA	NA
10	13751857	13755406	ENSORLG00020014707	High *π* & High *Tajima's D*		15145116	15147689	ENSORLG00000024089	NA	NA
10	13744111	13751389	ENSORLG00020014715	High *π* & High *Tajima's D*		NA	NA	NA	NA	NA
13	18550367	18580090	ENSORLG00020017043	High *π* & High *Tajima's D*		20011319	20036265	ENSORLG00000009995	NA	Alpha‐2‐macroglobulin‐like
15	8083986	8086506	ENSORLG00020019488	High *π* & High *Tajima's D*		33111565	33113634	ENSORLG00000028549	NA	NA
15	8078318	8098989	ENSORLG00020019523	High *π* & High *Tajima's D*		9424544	9444274	ENSORLG00000023586	rasgrp3	Ras guanyl releasing protein 3
15	8052474	8078729	ENSORLG00020019552	High *π* & High *Tajima's D*		9395788	9413313	ENSORLG00000029459	NA	Cadherin‐1‐like
16	14448063	14450527	ENSORLG00020018130	High *π* & High *Tajima's D*		15172499	15174941	ENSORLG00000006426	si:dkey‐238d18.3	Chymotrypsin‐like protease ctrl‐1
16	14443079	14447576	ENSORLG00020018160	High *π* & High *Tajima's D*		15169313	15172486	ENSORLG00000006386	hsp70‐3	Heat shock protein 70
16	14438752	14442673	ENSORLG00020018190	High *π* & High *Tajima's D*		NA	NA	NA	NA	NA
16	14429796	14430853	ENSORLG00020018201	High *π* & High *Tajima's D*		15151780	15159157	ENSORLG00000030376	NA	NA
16	14416481	14422452	ENSORLG00020018235	High *π* & High *Tajima's D*		15145450	15151647	ENSORLG00000025278	si:dkey‐238d18.5	Sialic acid‐binding ig‐like lectin 13
16	14394584	14400153	ENSORLG00020018248	High *π* & High *Tajima's D*		15127765	15137039	ENSORLG00000022612	NA	
18	4296761	4343165	ENSORLG00020018505	High *π* & High *Tajima's D*		5020396	5090035	ENSORLG00000003427	dysf	Dysferlin; limb girdle muscular dystrophy 2b (autosomal recessive)
18	6888243	6905164	ENSORLG00020016345	High *π* & High *Tajima's D*		7876025	7900570	ENSORLG00000004084	si:dkey‐183c6.7	Urea transporter 2
18	6868637	6882946	ENSORLG00020016399	High *π* & High *Tajima's D*		7857005	7870711	ENSORLG00000004069	si:dkey‐183c6.8	Protein o‐glcnacase
18	6921520	6929974	ENSORLG00020016310	High *π* & High *Tajima's D*		7927418	7932247	ENSORLG00000004098	syt4	Synaptotagmin 4
19	6789493	6810180	ENSORLG00020015491	High *π* & High *Tajima's D*		7825934	7844573	ENSORLG00000022047	NA	Integrin alpha‐m‐like
19	6761435	6781383	ENSORLG00020015534	High *π* & High *Tajima's D*		7804876	7942152	ENSORLG00000006425	NA	Integrin alpha‐m‐like
19	6745228	6753772	ENSORLG00020015572	High *π* & High *Tajima's D*		7789311	7797482	ENSORLG00000006367	aldoa	Fructose‐bisphosphate aldolase a
20	21349246	21357253	ENSORLG00020004209	High *π* & High *Tajima's D*		22977903	22986925	ENSORLG00000012793	olpks	Probable polyketide synthase 16
20	21304002	21345229	ENSORLG00020004243	High *π* & High *Tajima's D*		22957453	22969599	ENSORLG00000012785	si:dkey‐61p9.11	Equisetin synthetase eqxs
20	21296375	21303981	ENSORLG00020004278	High *π* & High *Tajima's D*		22949358	22957432	ENSORLG00000012778	rdh12l	Retinol dehydrogenase 12
24	9533858	9535153	ENSORLG00020007779	High *π* & High *Tajima's D*		NA	NA	NA	NA	NA
24	9515400	9516773	ENSORLG00020007787	High *π* & High *Tajima's D*		10708690	10716776	ENSORLG00000015595	NA	Adhesion g protein‐coupled receptor f7
10	13888077	13912307	ENSORLG00020014556	High *π* & High *Tajima's D* & High Intersexual‐*Fst*		15279728	15299751	ENSORLG00000023399	NA	NA
10	13879152	13885492	ENSORLG00020014569	High *π* & High *Tajima's D* & High Intersexual‐*Fst*		15272198	15278728	ENSORLG00000021994	pcdh2aa1	Protocadherin 2 alpha a 1
10	13868395	13871700	ENSORLG00020014579	High *π* & High *Tajima's D* & High Intersexual‐*Fst*		15252344	15258980	ENSORLG00000028208	si:ch73‐379j16.2	Si:ch73‐379j16.2
10	13857738	13868099	ENSORLG00020014584	High *π* & High *Tajima's D* & High Intersexual‐*Fst*		15246909	15255840	ENSORLG00000030495	NA	NA
18	26444393	26453551	ENSORLG00020000289	High *π* & High *Tajima's D* & High Intersexual‐*Fst*		29482757	29487920	ENSORLG00000012069	trmt10a	Trna methyltransferase 10a
18	26485117	26493277	ENSORLG00020011493	High *π* & High *Tajima's D* & High Intersexual‐*Fst*		29521943	29523823	ENSORLG00000012074	HACD4	3‐hydroxyacyl‐coa dehydratase 4
18	26469619	26472645	ENSORLG00020011502	High *π* & High *Tajima's D* & High Intersexual‐*Fst*		29504417	29508460	ENSORLG00000023851	C4orf54	Uncharacterized loc101158890
1	16044632	16051660	ENSORLG00020000827	High intersexual *FST* & Sex chromosome		16957480	16964480	ENSORLG00000007131	sh3gl2a	Endophilin‐a1
1	16095698	16185756	ENSORLG00020018825	High intersexual *FST* & Sex chromosome		17008064	17098337	ENSORLG00000007144	cntln	Centlein
1	16489531	16490704	ENSORLG00020018579	High intersexual *FST* & Sex chromosome		17418780	17421833	ENSORLG00000026982	NA	Snrna‐activating protein complex subunit 3
1	16588905	16615221	ENSORLG00020001088	High intersexual *FST* & Sex chromosome		17630042	17654826	ENSORLG00000007283	herc3	Hect and rld domain containing e3 ubiquitin protein ligase 3
1	16525893	16565918	ENSORLG00020018565	High intersexual *FST* & Sex chromosome		17479219	17485210	ENSORLG00000029140	NA	Pc4 and sfrs1‐interacting protein‐like
1	16615698	16669177	ENSORLG00020018486	High intersexual *FST* & Sex chromosome		17660922	17712088	ENSORLG00000007307	fam13a	Family with sequence similarity 13 member a
1	18745345	18759207	ENSORLG00020015076	High intersexual *FST* & Sex chromosome		19766500	19780329	ENSORLG00000007966	klhl2	Kelch like family member 2
1	18739649	18742749	ENSORLG00020015100	High intersexual *FST* & Sex chromosome		19760602	19763765	ENSORLG00000007946	med28	Mediator complex subunit 28
1	18730994	18738655	ENSORLG00020015111	High intersexual *FST* & Sex chromosome		19751385	19760592	ENSORLG00000007934	lap3	Leucine aminopeptidase 3
1	18726869	18730933	ENSORLG00020015120	High intersexual *FST* & Sex chromosome		19746989	19751364	ENSORLG00000007911	clrn2	Clarin 2
1	18713646	18723662	ENSORLG00020015154	High intersexual *FST* & Sex chromosome		19733177	19743539	ENSORLG00000007907	tapt1b	Transmembrane anterior posterior transformation protein 1 homolog
1	18676422	18712929	ENSORLG00020015282	High intersexual *FST* & Sex chromosome		19701007	19732465	ENSORLG00000007886	prom1b	Prominin‐1‐a
1	18947066	18961038	ENSORLG00020014355	High intersexual *FST* & Sex chromosome		19970331	19981517	ENSORLG00000008106	frg1	Fshd region gene 1
2	1815638	1896706	ENSORLG00020012898	High intersexual *FST*		1990112	2038696	ENSORLG00000000040	NA	Inactive dipeptidyl peptidase 10
2	13342593	13346123	ENSORLG00020021191	High intersexual *FST*		15338292	15341900	ENSORLG00000003049	fam117ba	Family with sequence similarity 117 member b
2	13327186	13362782	ENSORLG00020021247	High intersexual *FST*		15347566	15358175	ENSORLG00000003074	ical1	Islet cell autoantigen 1 like
2	13304700	13343510	ENSORLG00020021294	High intersexual *FST*		15310965	15334523	ENSORLG00000003034	bmpr2a	Bone morphogenetic protein receptor type 2
2	13298800	13300913	ENSORLG00020021312	High intersexual *FST*		NA	NA	NA	NA	NA
3	6291985	6294177	ENSORLG00020001627	High intersexual *FST*	^a^	NA	NA	NA	NA	NA
3	18298559	18317505	ENSORLG00020011438	High intersexual *FST*		19778881	19796865	ENSORLG00000006978	scube2	Signal peptide; cub domain and egf like domain containing 2
3	18292744	18297696	ENSORLG00020011452	High intersexual *FST*		19772871	19778378	ENSORLG00000006942	tmem9b	Tmem9 domain family member b
3	18237778	18288847	ENSORLG00020011501	High intersexual *FST*		19723107	19771274	ENSORLG00000006916	dennd2b	Denn domain containing 2b
3	24291880	24475255	ENSORLG00020002219	High intersexual *FST*		25881874	26007566	ENSORLG00000011860	mdga1	Mam domain containing glycosylphosphatidylinositol anchor 1
3	26648245	26650260	ENSORLG00020000963	High intersexual *FST*		28252114	28257573	ENSORLG00000025039	NA	NA
3	26639066	26646644	ENSORLG00020021696	High intersexual *FST*		28228605	28234786	ENSORLG00000013118	chrna3	Neuronal acetylcholine receptor subunit alpha‐3
3	26628176	26639045	ENSORLG00020021709	High intersexual *FST*		28216247	28228584	ENSORLG00000013114	chrnb4	Neuronal acetylcholine receptor subunit beta‐4‐like
3	26594294	26605478	ENSORLG00020021737	High intersexual *FST*		28180745	28192791	ENSORLG00000013097	ube2q1	Ubiquitin‐conjugating enzyme e2 q2
4	20126970	20131803	ENSORLG00020004074	High intersexual *FST*		1493311	1496392	ENSORLG00000023122	NA	NA
4	20787654	20981028	ENSORLG00020003543	High intersexual *FST*		20873275	21051957	ENSORLG00000012583	gmds	Gdp‐mannose 4;6‐dehydratase
4	20747157	20782766	ENSORLG00020003627	High intersexual *FST*		20836464	20868039	ENSORLG00000012568	mylk4b	Myosin light chain kinase family member 4
5	10401835	10480066	ENSORLG00020017613	High intersexual *FST*		10552102	10625956	ENSORLG00000005301	KIF5A	Kinesin heavy chain
5	13089824	13215434	ENSORLG00020014489	High intersexual *FST*		13322255	13447828	ENSORLG00000021803	TAFA3	Protein fam19a2
6	11817874	11903017	ENSORLG00020013475	High intersexual *FST*	NA	14105716	14191498	ENSORLG00000004498	PDE3A	Cgmp‐inhibited 3′
6	17913240	18029353	ENSORLG00020001699	High intersexual *FST*	NA	20291195	20419508	ENSORLG00000009936	SHANK3	Sh3 and multiple ankyrin repeat domains 3
6	24885780	24891423	ENSORLG00020013956	High intersexual *FST*	NA	27374042	27381757	ENSORLG00000014959	b3gnt9	Udp‐glcnac:betagal beta‐1;3‐n‐acetylglucosaminyltransferase 9
6	24875941	24883885	ENSORLG00020013997	High intersexual *FST*	NA	27365194	27370928	ENSORLG00000014949	bbs2	Bardet‐biedl syndrome 2
6	24865343	24875309	ENSORLG00020014012	High intersexual *FST*	NA	27347053	27357411	ENSORLG00000014911	setd6	Set domain containing 6; protein lysine methyltransferase
6	24859680	24868607	ENSORLG00020014130	High intersexual *FST*	NA	27339455	27349477	ENSORLG00000014893	utp4	Utp4 small subunit processome component
6	24857883	24859786	ENSORLG00020014161	High intersexual *FST*	NA	27337281	27339560	ENSORLG00000023926	chtf8	Chromosome transmission fidelity factor 8
6	24842555	24854574	ENSORLG00020014184	High intersexual *FST*	NA	27323615	27335940	ENSORLG00000025653	has3	Hyaluronan synthase 3
7	19254841	19260090	ENSORLG00020000417	High intersexual *FST*	NA	19682581	19685008	ENSORLG00000013132	taf11	Tata‐box binding protein associated factor 11
7	19293186	19330176	ENSORLG00020015747	High intersexual *FST*	NA	19724867	19756895	ENSORLG00000013207	pacsin1a	Protein kinase c and casein kinase substrate in neurons 1
7	19283759	19293413	ENSORLG00020015772	High intersexual *FST*	NA	19713269	19722662	ENSORLG00000013188	ILRUN	Uncharacterized protein c6orf106 homolog
7	19257914	19279969	ENSORLG00020015796	High intersexual *FST*	NA	19687661	19704803	ENSORLG00000013171	uhrf1bp1	Uhrf1 binding protein 1
7	19197329	19257267	ENSORLG00020016010	High intersexual *FST*	NA	19628143	19682115	ENSORLG00000013119	ANKS1A	Ankyrin repeat and sterile alpha motif domain containing 1a
7	25828098	25834561	ENSORLG00020000120	High intersexual *FST*	NA	NA	NA	NA	NA	NA
7	25834657	25842235	ENSORLG00020005759	High intersexual *FST*	NA	26873508	26875660	ENSORLG00000016726	NA	NA
8	19685745	19687176	ENSORLG00020004779	High intersexual *FST*		21074054	21076769	ENSORLG00000015364	si:dkeyp‐69e1.8	Si:dkeyp‐69e1.8
8	19677319	19682779	ENSORLG00020004813	High intersexual *FST*		21064350	21070044	ENSORLG00000015359	hmgxb4a	Hmg‐box containing 4
8	19668309	19677235	ENSORLG00020004834	High intersexual *FST*		21054548	21062742	ENSORLG00000015347	ankrd54	Ankyrin repeat domain 54
8	19660528	19669265	ENSORLG00020004851	High intersexual *FST*		21046127	21054527	ENSORLG00000015336	foxred2	Fad dependent oxidoreductase domain containing 2
8	19657236	19659888	ENSORLG00020004867	High intersexual *FST*		21043365	21047342	ENSORLG00000010457	hmox1a	Heme oxygenase
8	19649879	19659888	ENSORLG00020004893	High intersexual *FST*		21035623	21046048	ENSORLG00000010517	mcm5	Minichromosome maintenance complex component 5
9	741969	744826	ENSORLG00020000385	High intersexual *FST*		1173088	1176083	ENSORLG00000024387	dmrt3a	Doublesex and mab‐3 related transcription factor 3
9	744086	777818	ENSORLG00020013974	High intersexual *FST*		1178416	1213195	ENSORLG00000020817	dmy	*Oryzias latipes* doublesex‐ and mab‐3‐related transcription factor 1 (dmy)
9	728230	736344	ENSORLG00020014026	High intersexual *FST*		1154532	1163506	ENSORLG00000022893	dmrt2a	Doublesex‐ and mab‐3‐related transcription factor 2
9	782359	791810	ENSORLG00020013869	High intersexual *FST*		1219912	1235054	ENSORLG00000020812	kank1a	Kn motif and ankyrin repeat domains 1
9	12245205	12246254	ENSORLG00020015479	High intersexual *FST*		12863091	12864140	ENSORLG00000006867	cmklr1	Chemerin chemokine‐like receptor 1
9	12220457	12241016	ENSORLG00020015511	High intersexual *FST*		12837628	12856103	ENSORLG00000006858	wscd2	Wsc domain containing 2
9	17024841	17172124	ENSORLG00020005296	High intersexual *FST*		17867468	17955693	ENSORLG00000011900	TTC28	Tetratricopeptide repeat domain 28
9	25181055	25386489	ENSORLG00020019429	High intersexual *FST*		26690745	26888256	ENSORLG00000016270	unc5db	Netrin receptor unc5d
10	12544435	12553546	ENSORLG00020017288	High intersexual *FST*		13882739	13890196	ENSORLG00000003300	NA	NA
10	12537461	12543166	ENSORLG00020017314	High intersexual *FST*		13878925	13885142	ENSORLG00000022305	NA	Zinc finger cchc domain‐containing protein 7
10	12526040	12537434	ENSORLG00020017417	High intersexual *FST*		13865558	13878904	ENSORLG00000003296	NA	E3 ubiquitin‐protein ligase rnf38
10	12498962	12510257	ENSORLG00020017460	High intersexual *FST*		13839263	13854156	ENSORLG00000003262	chrnb5b	Neuronal acetylcholine receptor subunit beta‐2‐like
10	13828993	13834393	ENSORLG00020014597	High intersexual *FST*		NA	NA	NA	NA	NA
10	13817466	13825173	ENSORLG00020014603	High intersexual *FST*		15207915	15210299	ENSORLG00000004181	pcdh2aa15	Protocadherin 2 alpha a 15
10	13811314	13816439	ENSORLG00020014615	High intersexual *FST*		15198651	15206827	ENSORLG00000027606	NA	NA
12	18946735	18950787	ENSORLG00020008961	High intersexual *FST*		NA	NA	NA	NA	NA
12	18802173	19105420	ENSORLG00020008990	High intersexual *FST*		20129547	20441286	ENSORLG00000029567	cntfr	Ciliary neurotrophic factor receptor
14	7438920	7443530	ENSORLG00020016403	High intersexual *FST*		8815295	8819652	ENSORLG00000003011	nf2b	Merlin
14	7431336	7437944	ENSORLG00020016439	High intersexual *FST*		8809648	8814397	ENSORLG00000002959	mettl27	Methyltransferase like 27
14	7425426	7428024	ENSORLG00020016449	High intersexual *FST*		8802872	8807120	ENSORLG00000002938	Zgc:136892	Zgc:136892
14	7419741	7420377	ENSORLG00020016493	High intersexual *FST*		NA	NA	NA	NA	NA
14	7416655	7417294	ENSORLG00020016508	High intersexual *FST*		NA	NA	NA	NA	NA
14	7414252	7414885	ENSORLG00020016569	High intersexual *FST*		NA	NA	NA	NA	NA
14	7412866	7413729	ENSORLG00020016595	High intersexual *FST*		8785449	8790708	ENSORLG00000002920	Zgc:112437	Zgc:112437
14	7410314	7412370	ENSORLG00020016600	High intersexual *FST*		8787803	8789690	ENSORLG00000002915	NA	NA
14	7408836	7411740	ENSORLG00020016642	High intersexual *FST*		8785802	8786806	ENSORLG00000030640	cldnb	Claudin b
14	7397590	7401586	ENSORLG00020016661	High intersexual *FST*		8774574	8778569	ENSORLG00000002912	cldnh	Claudin h
14	17672898	17835337	ENSORLG00020020426	High intersexual *FST*		19292539	19470054	ENSORLG00000010408	gabra3	Gamma‐aminobutyric acid receptor subunit alpha‐3
15	12706778	13011257	ENSORLG00020015018	High intersexual *FST*		19834800	20171735	ENSORLG00000013099	astn1	Astrotactin 1
16	10030070	10095207	ENSORLG00020022638	High intersexual *FST*		10652932	10725587	ENSORLG00000004892	NA	Capping protein regulator and myosin 1 linker 1
16	18833424	18854905	ENSORLG00020007116	High intersexual *FST*		19714295	19733726	ENSORLG00000011481	prkcg	Protein kinase c alpha type
16	18783978	18815899	ENSORLG00020007146	High intersexual *FST*		19662047	19700707	ENSORLG00000011448	grin2db	Glutamate receptor ionotropic; nmda 2d
16	20925903	20927400	ENSORLG00020000015	High intersexual *FST*		21775705	21780637	ENSORLG00000028920	mrpl17	Mitochondrial ribosomal protein l17
16	20938965	20953752	ENSORLG00020001700	High intersexual *FST*		21791958	21807977	ENSORLG00000013615	cblc	E3 ubiquitin‐protein ligase cbl
16	20934329	20936089	ENSORLG00020001719	High intersexual *FST*		21788067	21789754	ENSORLG00000013603	tryp	Trypsinogen
16	20927750	20929899	ENSORLG00020001739	High intersexual *FST*		21780464	21782800	ENSORLG00000022909	NA	Trypsin‐2
16	20868591	20923547	ENSORLG00020001768	High intersexual *FST*		21723115	21777233	ENSORLG00000013550	tmem145	Transmembrane protein 145
17	2541503	2541572	ENSORLG00020006355	High intersexual *FST*		NA	NA	NA	NA	NA
17	2541503	2541571	ENSORLG00020006363	High intersexual *FST*		NA	NA	NA	NA	NA
17	2541103	2541171	ENSORLG00020006370	High intersexual *FST*		NA	NA	NA	NA	NA
17	2541103	2541170	ENSORLG00020006375	High intersexual *FST*		NA	NA	NA	NA	NA
17	2540768	2540877	ENSORLG00020006380	High intersexual *FST*		NA	NA	NA	NA	NA
17	2540635	2540925	ENSORLG00020006390	High intersexual *FST*		NA	NA	NA	NA	NA
17	2539421	2543505	ENSORLG00020006406	High intersexual *FST*		3740934	3743752	ENSORLG00000003239	rps8a	Ribosomal protein s8
17	2531459	2539209	ENSORLG00020006479	High intersexual *FST*		3732668	3740937	ENSORLG00000003223	hectd3	Hect domain e3 ubiquitin protein ligase 3
17	2520540	2528971	ENSORLG00020006504	High intersexual *FST*		3718460	3718690	ENSORLG00000029279	NA	NA
17	2508785	2515810	ENSORLG00020006525	High intersexual *FST*		3706471	3713201	ENSORLG00000026471	NA	Uncharacterized loc105356108
17	2479624	2500906	ENSORLG00020006551	High intersexual *FST*		NA	NA	NA	NA	NA
18	19717391	19763828	ENSORLG00020017890	High intersexual *FST*		22473420	22531351	ENSORLG00000026635	NA	NA
18	19702454	19707736	ENSORLG00020017900	High intersexual *FST*		22462420	22467817	ENSORLG00000010106	NA	NA
18	19701294	19706879	ENSORLG00020017911	High intersexual *FST*		NA	NA	NA	NA	NA
18	19692672	19701425	ENSORLG00020017936	High intersexual *FST*		22455784	22463821	ENSORLG00000010098	si:dkey‐46i9.6	Si:dkey‐46i9.6
18	19797475	19797533	ENSORLG00020017844	High intersexual *FST*		NA	NA	NA	NA	NA
18	19787540	19827853	ENSORLG00020017859	High intersexual *FST*		22555625	22560732	ENSORLG00000026388	NA	Complement c1q subcomponent subunit c
18	24062797	24083674	ENSORLG00020013519	High intersexual *FST*		27104496	27121459	ENSORLG00000010894	mtmr1a	Myotubularin‐related protein 2
18	24054026	24060424	ENSORLG00020013544	High intersexual *FST*		27085807	27102302	ENSORLG00000024407	NA	Cd99 antigen‐like protein 2
18	24048036	24054685	ENSORLG00020013560	High intersexual *FST*		27082336	27088975	ENSORLG00000025391	NA	NA
18	26444393	26453551	ENSORLG00020000289	High intersexual *FST*		29482757	29487920	ENSORLG00000012069	trmt10a	Trna methyltransferase 10a
18	26440322	26445915	ENSORLG00020011532	High intersexual *FST*		NA	NA	NA	NA	NA
18	26410605	26435828	ENSORLG00020011567	High intersexual *FST*		29454502	29468096	ENSORLG00000012050	slc1a1	Solute carrier family 1 member 1
19	11173358	11180003	ENSORLG00020000759	High intersexual *FST*		12320732	12325432	ENSORLG00000029575	chac2	Chac cation transport regulator homolog 2
19	11166261	11177827	ENSORLG00020004822	High intersexual *FST*		12315099	12320970	ENSORLG00000023995	NA	NA
19	11348427	11353857	ENSORLG00020004338	High intersexual *FST*		12510698	12516230	ENSORLG00000030175	kcng3	Potassium voltage‐gated channel subfamily g member 3
19	11336097	11344535	ENSORLG00020004351	High intersexual *FST*		12493918	12502944	ENSORLG00000027806	cox7a2l	Cytochrome c oxidase subunit 7a‐related protein; mitochondrial
19	11324453	11331016	ENSORLG00020004368	High intersexual *FST*		12484090	12490269	ENSORLG00000026182	pigf	Phosphatidylinositol glycan anchor biosynthesis class f
19	11320550	11324374	ENSORLG00020004374	High intersexual *FST*		12478229	12484502	ENSORLG00000027213	cript	Cxxc repeat containing interactor of pdz3 domain
19	11312510	11320080	ENSORLG00020004411	High intersexual *FST*		12475228	12478157	ENSORLG00000018755	gch2	Gtp cyclohydrolase 1
19	11301301	11310604	ENSORLG00020004470	High intersexual *FST*		12450714	12461672	ENSORLG00000010268	msh2	Muts homolog 2
19	11288431	11300863	ENSORLG00020004555	High intersexual *FST*		12437842	12450268	ENSORLG00000010222	NA	F‐box only protein 11
19	14934770	14954329	ENSORLG00020001295	High intersexual *FST*	^a^	16851739	16871548	ENSORLG00000018336	si:ch211‐214e3.5	Zinc finger protein 518a
19	14817498	14920654	ENSORLG00020001334	High intersexual *FST*	^a^	16686446	16691314	ENSORLG00000022086	NA	NA
20	2890552	2892419	ENSORLG00020011085	High intersexual *FST*		NA	NA	NA	NA	NA
20	2888805	2890302	ENSORLG00020011095	High intersexual *FST*		NA	NA	NA	NA	NA
20	2813061	2850187	ENSORLG00020011418	High intersexual *FST*		3439071	3476121	ENSORLG00000003235	unm_hu7910	Aspartyl/asparaginyl beta‐hydroxylase
21	9434173	9455895	ENSORLG00020010687	High intersexual *FST*		10141652	10162456	ENSORLG00000012399	slc9a2	Sodium/hydrogen exchanger 2
21	9410532	9431589	ENSORLG00020010755	High intersexual *FST*		10117493	10133137	ENSORLG00000012384	inpp4aa	Inositol polyphosphate‐4‐phosphatase type i a
21	9395851	9401743	ENSORLG00020010781	High intersexual *FST*		10091716	10098084	ENSORLG00000027912	si:dkey‐4e7.3	Inosine‐uridine preferring nucleoside hydrolase
22	1158136	1234253	ENSORLG00020002902	High intersexual *FST*		1597531	1671344	ENSORLG00000009443	slc8a3	Solute carrier family 8 member a3
22	1241386	1252081	ENSORLG00020002890	High intersexual *FST*		1678344	1688797	ENSORLG00000009460	map3k9	Mitogen‐activated protein kinase kinase kinase 9
22	25284471	25306192	ENSORLG00020008481	High intersexual *FST*		25899890	25919950	ENSORLG00000018203	wdr35	Wd repeat domain 35
22	25277254	25279266	ENSORLG00020008515	High intersexual *FST*		25889150	25896418	ENSORLG00000027908	NA	NA
22	25258663	25265527	ENSORLG00020008541	High intersexual *FST*		25844380	25851146	ENSORLG00000018198	NA	Cell division cycle 25b
23	1539376	1572547	ENSORLG00020005491	High intersexual *FST*		1943366	1958181	ENSORLG00000009131	SLC16A7	Solute carrier family 16 member 7

*Note:* Annotation information was based on the Ensembl release 109, *Oryzias latipes* HdrR strain (ASM223467v1) and *O. sakaizumii* HNI strain (ASM223471v1).

Abbreviation: NA, Not available.

^a^ Centromere region were inferred based on the synteny block positions of HdrR and HNI references using ntSynt (Table A3), and overlap determined within 1 Mb of the centromere regions.

**TABLE A8:** Top 20 gene ontology (GO) terms from enrichment analysis using ShinyGO ver. 0.82, sorted by ascending enrichment false discovery rate (FDR) (Significance threshold, *: *FDR* < 0.01; a: 0.01 < *FDR* < 0.05; N.S.: 0.05 < *FDR*).

Population statistics	Enrichment.FDR	nGenes	Pathway.genes	Fold.enrichment	Significance threshold	Pathway	Genes
iHS outlier	0.025056	3	17	54.79876161	a	GO:0032266 phosphatidylinositol‐3‐phosphate binding	snx12 bbs5 vps36
Fd (EJ→sak)	4.11E−12	7	35	168.5714286	*	GO:0001594 trace‐amine receptor activity	ENSORLG00000013895 ENSORLG00000013916 ENSORLG00000023929 ENSORLG00000024077 ENSORLG00000025011 ENSORLG00000025608 ENSORLG00000028977
Fd (EJ→sak)	9.36E−10	7	80	73.75	*	GO:0008227 G protein‐coupled amine receptor activity	ENSORLG00000013895 ENSORLG00000013916 ENSORLG00000023929 ENSORLG00000024077 ENSORLG00000025011 ENSORLG00000025608 ENSORLG00000028977
Fd (EJ→sak)	8.41E−06	9	686	11.05789254	*	GO:0004930 G protein‐coupled receptor activity	ENSORLG00000010357 ENSORLG00000013895 ENSORLG00000013916 ENSORLG00000023929 ENSORLG00000024077 ENSORLG00000024842 ENSORLG00000025011 ENSORLG00000025608 ENSORLG00000028977
Fd (EJ→sak)	5.93E−05	9	894	8.485139022	*	GO:0007186 G protein‐coupled receptor signaling pathway	ENSORLG00000024842 ENSORLG00000010357 ENSORLG00000013895 ENSORLG00000013916 ENSORLG00000023929 ENSORLG00000024077 ENSORLG00000025011 ENSORLG00000025608 ENSORLG00000028977
Fd (EJ→sak)	0.000189	9	1056	7.183441558	*	GO:0004888 transmembrane signaling receptor activity	ENSORLG00000010357 ENSORLG00000013895 ENSORLG00000013916 ENSORLG00000023929 ENSORLG00000024077 ENSORLG00000024842 ENSORLG00000025011 ENSORLG00000025608 ENSORLG00000028977
Fd (EJ→sak)	0.000398	9	1205	6.295198577	*	GO:0038023 signaling receptor activity	ENSORLG00000010357 ENSORLG00000013895 ENSORLG00000013916 ENSORLG00000023929 ENSORLG00000024077 ENSORLG00000024842 ENSORLG00000025011 ENSORLG00000025608 ENSORLG00000028977
Fd (EJ→sak)	0.000398	9	1205	6.295198577	*	GO:0060089 molecular transducer activity	ENSORLG00000010357 ENSORLG00000013895 ENSORLG00000013916 ENSORLG00000023929 ENSORLG00000024077 ENSORLG00000024842 ENSORLG00000025011 ENSORLG00000025608 ENSORLG00000028977
Fd (EJ→sak)	0.004971	2	13	129.6703297	*	GO:0035805 egg coat	ENSORLG00000010086 ENSORLG00000010134
Fd (EJ→sak)	0.004971	2	13	129.6703297	*	GO:0035804 structural constituent of egg coat	ENSORLG00000010086 ENSORLG00000010134
Fd (EJ→sak)	0.006726	2	18	93.65079365	*	GO:0007339 binding of sperm to zona pellucida	ENSORLG00000010086 ENSORLG00000010134
Fd (EJ→sak)	0.006726	2	18	93.65079365	*	GO:0009988 cell–cell recognition	ENSORLG00000010086 ENSORLG00000010134
Fd (EJ→sak)	0.006726	2	18	93.65079365	*	GO:0035036 sperm‐egg recognition	ENSORLG00000010086 ENSORLG00000010134
Fd (EJ→sak)	0.006726	2	17	99.15966387	*	GO:0032190 acrosin binding	ENSORLG00000010086 ENSORLG00000010134
Fd (EJ→sak)	0.019179	13	4339	2.525269154	a	GO:0051716 cellular response to stimulus	ENSORLG00000006920 ENSORLG00000022172 ENSORLG00000024842 ENSORLG00000009232 ENSORLG00000010357 ENSORLG00000013895 ENSORLG00000013916 ENSORLG00000023929 ENSORLG00000024077 ENSORLG00000025011 ENSORLG00000025608 ENSORLG00000028977 ENSORLG00000030635
Fd (EJ→sak)	0.021125	2	34	49.57983193	a	GO:2000241 regulation of reproductive process	ENSORLG00000010086 ENSORLG00000010134
Fd (EJ→sak)	0.02208	2	37	45.55984556	a	GO:0007338 single fertilization	ENSORLG00000010086 ENSORLG00000010134
Fd (EJ→sak)	0.02208	2	37	45.55984556	a	GO:0009566 fertilization	ENSORLG00000010086 ENSORLG00000010134
Fd (EJ→sak)	0.05719	11	3740	2.478991597	N.S.	GO:0007165 signal transduction	ENSORLG00000022172 ENSORLG00000024842 ENSORLG00000010357 ENSORLG00000013895 ENSORLG00000013916 ENSORLG00000023929 ENSORLG00000024077 ENSORLG00000025011 ENSORLG00000025608 ENSORLG00000028977 ENSORLG00000030635
Fd (EJ→sak)	0.067274	2	71	23.74245473	N.S.	Path:ola03250 Viral life cycle‐HIV‐1	ENSORLG00000009232 ENSORLG00000021917
Fd (EJ→sak)	0.067274	11	4033	2.29889129	N.S.	GO:0007154 cell communication	ENSORLG00000022172 ENSORLG00000024842 ENSORLG00000010357 ENSORLG00000013895 ENSORLG00000013916 ENSORLG00000023929 ENSORLG00000024077 ENSORLG00000025011 ENSORLG00000025608 ENSORLG00000028977 ENSORLG00000030635
High *π* and High *Tajima's D*	3.04E−10	10	198	29.0711998	*	GO:0007156 homophilic cell adhesion via plasma membrane adhesion molecules	ENSORLG00000021994 ENSORLG00000023399 ENSORLG00000023815 ENSORLG00000024089 ENSORLG00000024772 ENSORLG00000026864 ENSORLG00000028208 ENSORLG00000029341 ENSORLG00000029459 ENSORLG00000029952
High *π* and High *Tajima's D*	3.04E−10	12	379	18.22511101	*	GO:0098609 cell–cell adhesion	ENSORLG00000006425 ENSORLG00000022047 ENSORLG00000021994 ENSORLG00000023399 ENSORLG00000023815 ENSORLG00000024089 ENSORLG00000024772 ENSORLG00000026864 ENSORLG00000028208 ENSORLG00000029341 ENSORLG00000029459 ENSORLG00000029952
High *π* and High *Tajima's D*	4.41E−10	14	661	12.19143205	*	GO:0007155 cell adhesion	ENSORLG00000006425 ENSORLG00000021994 ENSORLG00000022047 ENSORLG00000022612 ENSORLG00000023399 ENSORLG00000023815 ENSORLG00000024089 ENSORLG00000024772 ENSORLG00000025278 ENSORLG00000026864 ENSORLG00000028208 ENSORLG00000029341 ENSORLG00000029459 ENSORLG00000029952
High *π* and High *Tajima's D*	5.43E−10	10	226	25.46945823	*	GO:0098742 cell–cell adhesion via plasma‐membrane adhesion molecules	ENSORLG00000021994 ENSORLG00000023399 ENSORLG00000023815 ENSORLG00000024089 ENSORLG00000024772 ENSORLG00000026864 ENSORLG00000028208 ENSORLG00000029341 ENSORLG00000029459 ENSORLG00000029952
High *π* and High *Tajima's D*	1.04E−08	13	705	10.61408061	*	GO:0005509 calcium ion binding	ENSORLG00000003427 ENSORLG00000004098 ENSORLG00000021994 ENSORLG00000023399 ENSORLG00000023586 ENSORLG00000023815 ENSORLG00000024089 ENSORLG00000024772 ENSORLG00000026864 ENSORLG00000028208 ENSORLG00000029341 ENSORLG00000029459 ENSORLG00000029952
High *π* and High *Tajima's D*	1.02E−06	21	3250	3.719324578	*	GO:0005886 plasma membrane	ENSORLG00000003427 ENSORLG00000004098 ENSORLG00000006386 ENSORLG00000006425 ENSORLG00000015375 ENSORLG00000021994 ENSORLG00000022047 ENSORLG00000022612 ENSORLG00000023399 ENSORLG00000023586 ENSORLG00000023815 ENSORLG00000024089 ENSORLG00000024303 ENSORLG00000024772 ENSORLG00000025278 ENSORLG00000026864 ENSORLG00000028208 ENSORLG00000029341 ENSORLG00000004084 ENSORLG00000029459 ENSORLG00000029952
High *π* and High *Tajima's D*	4.92E−06	21	3580	3.37648181	*	GO:0071944 cell periphery	ENSORLG00000003427 ENSORLG00000004098 ENSORLG00000006386 ENSORLG00000006425 ENSORLG00000015375 ENSORLG00000021994 ENSORLG00000022047 ENSORLG00000022612 ENSORLG00000023399 ENSORLG00000023586 ENSORLG00000023815 ENSORLG00000024089 ENSORLG00000024303 ENSORLG00000024772 ENSORLG00000025278 ENSORLG00000026864 ENSORLG00000028208 ENSORLG00000029341 ENSORLG00000004084 ENSORLG00000029459 ENSORLG00000029952
High *π* and High *Tajima's D*	4.70E−05	3	11	156.9844789	*	GO:0000447 endonucleolytic cleavage in ITS1 to separate SSU‐rRNA from 5.8S rRNA and LSU‐rRNA from tricistronic rRNA transcript (SSU‐rRNA 5.8S rRNA LSU‐rRNA)	ENSORLG00000015403 ENSORLG00000022846 ENSORLG00000025827
High *π* and High *Tajima's D*	0.000103	3	15	115.1219512	*	GO:0000478 endonucleolytic cleavage involved in rRNA processing	ENSORLG00000015403 ENSORLG00000022846 ENSORLG00000025827
High *π* and High *Tajima's D*	0.000103	3	15	115.1219512	*	GO:0000479 endonucleolytic cleavage of tricistronic rRNA transcript (SSU‐rRNA 5.8S rRNA LSU‐rRNA)	ENSORLG00000015403 ENSORLG00000022846 ENSORLG00000025827
High *π* and High *Tajima's D*	0.000272	3	21	82.22996516	*	GO:0000466 maturation of 5.8S rRNA from tricistronic rRNA transcript (SSU‐rRNA 5.8S rRNA LSU‐rRNA)	ENSORLG00000015403 ENSORLG00000022846 ENSORLG00000025827
High *π* and High *Tajima's D*	0.000289	3	22	78.49223947	*	GO:0000469 cleavage involved in rRNA processing	ENSORLG00000015403 ENSORLG00000022846 ENSORLG00000025827
High *π* and High *Tajima's D*	0.000323	10	1045	5.508227331	*	GO:0005887 integral component of plasma membrane	ENSORLG00000006425 ENSORLG00000021994 ENSORLG00000022047 ENSORLG00000023399 ENSORLG00000023815 ENSORLG00000024089 ENSORLG00000024772 ENSORLG00000026864 ENSORLG00000028208 ENSORLG00000029341
High *π* and High *Tajima's D*	0.000372	10	1071	5.374507527	*	GO:0031226 intrinsic component of plasma membrane	ENSORLG00000006425 ENSORLG00000021994 ENSORLG00000022047 ENSORLG00000023399 ENSORLG00000023815 ENSORLG00000024089 ENSORLG00000024772 ENSORLG00000026864 ENSORLG00000028208 ENSORLG00000029341
High *π* and High *Tajima's D*	0.00043	3	27	63.95663957	*	GO:0030686 90S preribosome	ENSORLG00000015403 ENSORLG00000022846 ENSORLG00000025827
High *π* and High *Tajima's D*	0.00043	3	28	61.67247387	*	GO:0000460 maturation of 5.8S rRNA	ENSORLG00000015403 ENSORLG00000022846 ENSORLG00000025827
High *π* and High *Tajima's D*	0.00043	3	28	61.67247387	*	GO:0000462 maturation of SSU‐rRNA from tricistronic rRNA transcript (SSU‐rRNA 5.8S rRNA LSU‐rRNA)	ENSORLG00000015403 ENSORLG00000022846 ENSORLG00000025827
High *π* and High *Tajima's D*	0.000463	2	4	287.804878	*	GO:0004315 3‐oxoacyl‐[acyl‐carrier‐protein] synthase activity	ENSORLG00000012785 ENSORLG00000012793
High *π* and High *Tajima's D*	0.000831	3	36	47.96747967	*	GO:0030490 maturation of SSU‐rRNA	ENSORLG00000015403 ENSORLG00000022846 ENSORLG00000025827
High *π* and High *Tajima's D*	0.001885	3	48	35.97560976	*	GO:0042274 ribosomal small subunit biogenesis	ENSORLG00000015403 ENSORLG00000022846 ENSORLG00000025827
High *π* and High *Tajima's D* and High intersexual *FST*	0.000943	3	198	51.08225108	*	GO:0007156 homophilic cell adhesion via plasma membrane adhesion molecules	ENSORLG00000021994 ENSORLG00000023399 ENSORLG00000028208
High *π* and High *Tajima's D* and High intersexual *FST*	0.000943	3	226	44.75347661	*	GO:0098742 cell–cell adhesion via plasma‐membrane adhesion molecules	ENSORLG00000021994 ENSORLG00000023399 ENSORLG00000028208
High *π* and High *Tajima's D* and High intersexual *FST*	0.002925	3	379	26.68676969	*	GO:0098609 cell–cell adhesion	ENSORLG00000021994 ENSORLG00000023399 ENSORLG00000028208
High *π* and High *Tajima's D* and High intersexual *FST*	0.009487	3	661	15.30149125	*	GO:0007155 cell adhesion	ENSORLG00000021994 ENSORLG00000023399 ENSORLG00000028208
High *π* and High *Tajima's D* and High intersexual *FST*	0.009487	3	705	14.34650456	*	GO:0005509 calcium ion binding	ENSORLG00000021994 ENSORLG00000023399 ENSORLG00000028208
High *π* and High *Tajima's D* and High intersexual *FST*	0.009487	1	4	842.8571429	*	GO:0018812 3‐hydroxyacyl‐CoA dehydratase activity	ENSORLG00000012074
High *π* and High *Tajima's D* and High intersexual *FST*	0.009487	1	5	674.2857143	*	GO:0102158 very‐long‐chain 3‐hydroxyacyl‐CoA dehydratase activity	ENSORLG00000012074
High *π* and High *Tajima's D* and High intersexual *FST*	0.009487	1	5	674.2857143	*	GO:0102343 3‐hydroxy‐arachidoyl‐CoA dehydratase activity	ENSORLG00000012074
High *π* and High *Tajima's D* and High intersexual *FST*	0.009487	1	5	674.2857143	*	GO:0102344 3‐hydroxy‐behenoyl‐CoA dehydratase activity	ENSORLG00000012074
High *π* and High *Tajima's D* and High intersexual *FST*	0.009487	1	5	674.2857143	*	GO:0102345 3‐hydroxy‐lignoceroyl‐CoA dehydratase activity	ENSORLG00000012074
High *π* and High *Tajima's D* and High intersexual *FST*	0.015162	3	1045	9.67874231	a	GO:0005887 integral component of plasma membrane	ENSORLG00000021994 ENSORLG00000023399 ENSORLG00000028208
High *π* and High *Tajima's D* and High intersexual *FST*	0.015162	3	1071	9.443777511	a	GO:0031226 intrinsic component of plasma membrane	ENSORLG00000021994 ENSORLG00000023399 ENSORLG00000028208
High *π* and High *Tajima's D* and High intersexual *FST*	0.021654	1	15	224.7619048	a	GO:0030497 fatty acid elongation	ENSORLG00000012074
High *π* and High *Tajima's D* and High intersexual *FST*	0.021654	1	16	210.7142857	a	GO:0042761 very long‐chain fatty acid biosynthetic process	ENSORLG00000012074
High *π* and High *Tajima's D* and High intersexual *FST*	0.030284	1	24	140.4761905	a	GO:0000038 very long‐chain fatty acid metabolic process	ENSORLG00000012074
High *π* and High *Tajima's D* and High intersexual *FST*	0.046048	1	39	86.44688645	a	Path:ola00062 Fatty acid elongation	ENSORLG00000012074
High *π* and High *Tajima's D* and High intersexual *FST*	0.046656	1	42	80.27210884	a	Path:ola01040 Biosynthesis of unsaturated fatty acids	ENSORLG00000012074
High *π* and High *Tajima's D* and High intersexual *FST*	0.048236	1	46	73.29192547	a	GO:0030148 sphingolipid biosynthetic process	ENSORLG00000012074
High *π* and High *Tajima's D* and High intersexual *FST*	0.049645	1	50	67.42857143	a	GO:0016836 hydro‐lyase activity	ENSORLG00000012074
High *π* and High *Tajima's D* and High intersexual *FST*	0.052782	1	56	60.20408163	N.S.	GO:0016835 carbon–oxygen lyase activity	ENSORLG00000012074

**TABLE A9:** Results of integrated haplotype score in *Oryzias sakaizumii* calculated by selscan.

Chromosome	Position	*Z*‐transformed iHS	Ensembl gene ID (HNI)	Centromere region^a^	Chromosome	Position	*Z*‐transformed iHS	Ensembl gene ID (HNI)	Centromere region
1	3994631	−2.72592	NA		11	6536744	2.93138	NA	
1	8613654	−2.54229	NA		11	6664530	3.0129	ENSORLG00020005711	
1	8967958	−2.49842	NA		11	7397171	−2.43733	NA	
1	12112642	−2.59189	NA		11	8104081	−2.54622	NA	
1	15078135	3.2367	ENSORLG00020020644		11	9845516	3.06006	NA	
1	16006860	3.20753	ENSORLG00020019104		11	10701595	3.26476	NA	
1	16022393	2.92841	NA		11	10719147	3.20072	NA	
1	16364290	−3.14226	NA		11	12944306	−2.48683	NA	
1	16365897	−3.14226	ENSORLG00020018654		11	12974757	3.30743	NA	
1	16427285	3.51728	NA		11	12998887	3.01825	NA	
1	16457939	2.96118	NA		11	13232761	3.00713	NA	
1	16644163	3.32424	NA		11	13238443	2.93769	NA	
1	16659224	2.97147	NA		11	13282327	3.30047	NA	
1	16708856	2.93759	NA		11	13283373	3.36172	NA	
1	16733449	3.75719	NA		11	13465632	3.36238	NA	
1	16853980	2.9497	NA		11	16907852	3.09013	NA	
1	17091468	3.06475	NA		11	17399155	3.09319	NA	
1	17091901	4.28132	NA		11	17437439	−2.90175	ENSORLG00020004309	
1	17096701	4.33888	NA		11	17437460	−2.90561	NA	
1	17108444	2.93852	NA		11	17437833	−2.88788	NA	
1	17111755	4.33129	NA		11	17437835	−2.88788	NA	
1	17115634	3.34989	NA		11	18155921	3.2855	NA	
1	17119639	3.15034	NA		11	18544126	−2.58951	NA	
1	17719794	3.01661	NA		11	19337846	−2.64104	NA	
1	18054726	3.35904	NA		12	6666439	−2.40279	NA	
1	18054788	3.35904	NA		12	6666535	−2.40279	NA	
1	18075859	3.23119	NA		12	6719900	−2.41035	NA	
1	18078973	2.9825	NA		12	6777268	−2.40513	NA	
1	18410673	3.47476	NA		12	6866413	−2.48788	NA	
1	18420535	3.09702	NA		12	6982001	−2.53806	ENSORLG00020009339	
1	18422987	3.05303	NA		12	6982190	−2.42707	NA	
1	20067643	3.23162	NA		12	6988310	−2.50446	ENSORLG00020009327	
1	20197637	3.00618	NA		12	6998189	−2.60581	NA	
1	20753051	3.06552	NA		12	7100987	−2.46184	NA	
1	21268184	3.40435	NA		12	7596053	−2.49345	NA	
1	21466217	3.28181	NA		12	7596069	−2.49345	NA	
1	21503728	3.5671	ENSORLG00020011155		12	7604803	−2.52117	ENSORLG00020008279	
1	21528461	3.25748	ENSORLG00020011080		12	7605619	−2.52117	NA	
1	21588114	3.26061	NA		12	8221475	−2.69763	NA	
1	23028866	−2.68798	NA		12	9827759	−2.9083	NA	
1	25892144	−2.71288	NA		12	10515534	2.93293	NA	
1	29139238	2.94602	NA		12	12861033	2.91659	NA	
1	29516512	−2.55565	ENSORLG00020021086		12	13896517	3.42137	NA	
1	30176864	−2.51077	NA		12	15455319	−2.84396	NA	
2	1879134	2.93109	NA		12	23831516	−2.56171	NA	
2	2287407	3.01222	NA		12	23831650	−2.82512	NA	
2	2293778	3.49248	NA		12	26786380	−2.57815	NA	
2	10876739	2.98347	NA		13	850603	−2.78275	NA	
2	13701774	3.27489	NA		13	3364238	−2.61248	NA	
2	16138476	−2.48958	NA		13	3364336	−2.61248	NA	
2	16195538	−2.48196	NA		13	3366348	−2.41562	NA	
2	16197909	−2.76036	NA		13	3366349	−2.41562	NA	
2	17020670	3.04741	NA		13	3367510	−2.44622	NA	
2	17033202	−2.7456	NA		13	3369346	−2.44622	NA	
2	17033205	−2.7456	NA		13	3369384	−2.44622	NA	
2	17033599	−2.91971	NA		13	3369519	−2.44622	NA	
2	17039291	2.95117	NA		13	3560743	3.00833	NA	
2	17710318	−2.54503	NA		13	3561368	2.93618	NA	
2	17840873	−2.61152	NA		13	7538965	−2.84368	ENSORLG00020012816	
2	18164728	−2.8077	NA		13	7635005	−2.40959	NA	
3	4533301	−2.51788	NA		13	7715183	−2.48507	NA	
3	7811779	−2.45459	NA		13	7715271	−2.69445	NA	
3	7812047	−2.45459	NA		13	7736159	−2.62702	NA	
3	7813625	−2.45459	NA		13	7777851	−2.67456	NA	
3	7815739	−2.86946	NA		13	7778972	−2.57513	NA	
3	7819008	−2.66484	NA		13	7779019	−2.43867	NA	
3	8508275	3.6285	NA		13	7779062	−2.77308	NA	
3	8553245	3.20643	NA		13	7779113	−2.60476	NA	
3	8727719	−2.61961	NA		13	8625751	2.94085	NA	
3	9153079	−2.44719	NA		13	9445407	2.927	NA	
3	11226872	2.96226	NA		13	9782212	2.99164	NA	
3	11656336	3.16773	NA		13	9838133	3.13292	NA	
3	13375590	2.91654	NA		13	9851260	2.93071	NA	
3	14239925	−2.60155	NA		13	9851599	2.93071	NA	
3	15454070	−2.83773	NA		13	9854392	3.00697	NA	
3	15455609	−2.87042	NA		13	9854475	3.00697	NA	
3	15455870	−2.83576	NA		13	9855726	3.26032	NA	
3	15770080	−2.47978	NA		13	9855758	−3.02757	NA	
3	16209905	3.24953	NA		13	9857604	3.15534	NA	
3	16210776	3.23721	NA		13	9858148	3.01889	NA	
3	16704051	3.50243	NA		13	9858746	3.02763	NA	
3	16845064	−2.54078	NA		13	9860566	2.97904	NA	
3	17145676	−2.71838	NA		13	9860849	2.97904	NA	
3	17147490	−2.54592	NA		13	9860865	2.97904	NA	
3	17958199	3.03581	ENSORLG00020011602		13	9864211	2.97458	NA	
3	18229509	3.22233	NA		13	9868343	3.96499	NA	
3	23103277	−2.54326	NA		13	9891811	3.25025	NA	
3	23103477	−2.64557	NA		13	9891961	3.24108	NA	
3	23143883	−2.41372	NA		13	9891982	3.24108	NA	
3	23226916	3.13263	NA		13	9892080	3.24108	NA	
3	24932449	2.92874	ENSORLG00020001905		13	9892398	3.24108	NA	
3	24936376	2.93511	NA		13	9970526	3.55326	NA	
3	24937385	2.98542	NA		13	10049702	3.26471	NA	
3	25364699	3.06648	NA		13	10061825	3.46255	ENSORLG00020008694	
3	28829994	3.10652	NA		13	10063211	3.3234	NA	
3	29254576	3.06043	NA		13	10205702	3.40817	NA	
3	33462990	−2.60556	NA		13	10212240	3.36721	NA	
4	2337519	3.0925	NA		13	10212713	3.35755	NA	
4	2697079	−2.66678	NA		13	10213311	3.19136	NA	
4	4865851	−2.41668	NA		13	10215042	3.19136	NA	
4	6795010	−2.72296	NA		13	10215306	3.26402	NA	
4	6917376	−2.46269	NA		13	10220609	2.92131	NA	
4	6968444	−2.66504	NA		13	10221517	2.92131	NA	
4	6992089	−2.56193	NA		13	10224419	2.93193	NA	
4	6992095	−2.56193	NA		13	10225780	2.93193	NA	
4	6992135	−2.56193	NA		13	10233398	3.1041	NA	
4	7001206	−2.50167	NA		13	10233557	3.1041	NA	
4	7015078	−2.95858	NA		13	10233587	3.1041	NA	
4	7015116	−2.40183	NA		13	10233800	3.18857	NA	
4	7022462	−2.66009	ENSORLG00020004168		13	10234855	3.18857	NA	
4	7047214	−2.42042	NA		13	10236783	3.01465	NA	
4	7114508	−2.52984	ENSORLG00020004041		13	10328696	3.01907	NA	
4	7189342	−2.4862	NA		13	10488943	3.32411	NA	
4	7190192	−2.40634	NA		13	11707525	2.98842	NA	
4	7192745	−2.40508	ENSORLG00020003989		13	11730851	−2.49189	NA	
4	9120462	−2.46429	NA		13	12429599	−2.46946	NA	
4	9120466	−2.46429	NA		13	13083082	3.42679	NA	
4	9125348	−2.46429	NA		13	13092605	3.76531	NA	
4	9142306	−2.77623	NA		13	13357856	3.31337	NA	
4	9142596	−2.74186	NA		13	14325361	3.14839	NA	
4	9148753	−2.65551	NA		13	14325823	3.16762	NA	
4	9161005	−2.79042	NA		13	14368433	−3.09773	NA	
4	9490666	−2.60684	NA		13	15468242	−2.40307	NA	
4	9544712	−2.52672	NA		13	17602967	−3.01431	NA	
4	9752903	−2.40935	NA		13	21690853	3.10759	NA	
4	10507994	−2.81112	NA		13	23024140	−2.56563	NA	
4	10507996	−2.81112	NA		13	24160724	−2.41827	NA	
4	11213340	3.87122	NA		14	6420376	3.19057	ENSORLG00020019469	
4	12873251	2.98632	NA		14	6437064	3.31893	ENSORLG00020019369	
4	12911487	3.094	NA		14	6438521	3.02841	NA	
4	13204096	3.3512	NA		14	6518285	2.97752	NA	
4	13450432	3.05661	ENSORLG00020015450		14	6704728	3.06395	NA	
4	14476959	4.06278	NA		14	8381601	3.45119	NA	
4	14478801	4.23853	NA		14	9677044	−2.51023	ENSORLG00020011296	
4	14478802	4.23853	NA		14	9680106	−2.44673	NA	
4	14482331	3.27829	NA		14	9697694	−2.51882	NA	
4	14486918	3.9909	NA		14	9717724	−2.45315	NA	
4	14487694	3.73722	NA		14	9735289	−2.60296	NA	
4	14599910	3.16968	ENSORLG00020013576		14	9981473	−2.93554	NA	
4	14600076	3.17459	NA		14	9989883	−2.74631	NA	
4	14658136	2.96688	NA		14	9990446	−2.49123	NA	
4	14658942	2.96715	ENSORLG00020013411		14	9993944	−2.49668	ENSORLG00020011078	
4	14667660	3.0267	NA		14	9995170	−2.81454	NA	
4	14707031	3.00322	NA		14	10011633	−2.53764	NA	
4	14725634	−2.86022	NA		14	10012776	−2.65233	ENSORLG00020010939	
4	14732376	2.94551	ENSORLG00020013176		14	10024107	−2.88484	ENSORLG00020010886	
4	15917074	3.00016	NA		14	12057351	3.02538	NA	
4	15952527	3.94207	NA		14	13843335	−2.42204	NA	
4	20485030	−2.73025	NA		14	13843393	−2.63245	NA	
4	20871265	−2.93989	NA		14	13931613	3.24353	NA	
4	24495552	−2.466	NA		14	15103646	3.03395	ENSORLG00020021977	
4	24495554	−2.466	NA		14	15851628	2.92347	NA	
5	2598524	−2.4422	NA	^a^	14	15859085	3.39252	NA	
5	3153243	3.02337	NA	^a^	14	16264485	3.02753	NA	
5	4746136	−2.78663	NA		14	21116881	−2.52665	NA	
5	4757269	−3.03903	NA		14	21438123	−2.43194	NA	
5	4807522	−2.44451	NA		14	23092182	3.34473	NA	
5	4807637	−2.44451	NA		14	23940320	3.13317	NA	
5	4807638	−2.44451	NA		14	25930500	3.56011	NA	
5	4807875	−2.69563	NA		14	25930510	3.56011	NA	
5	4807877	−2.69563	NA		14	26061922	3.70049	ENSORLG00020008278	
5	4807950	−2.57252	NA		14	26079452	−2.46982	NA	
5	4812976	−2.49441	NA		14	26619593	−2.41742	NA	
5	4820385	−2.53472	NA		14	27316368	−2.40111	NA	
5	4821224	−2.43556	NA		15	4911570	−2.56694	NA	NA
5	7166010	−2.40054	NA		15	6875679	2.95584	NA	NA
5	7263130	−2.6887	NA		15	9928696	2.98084	NA	NA
5	7332365	−3.06507	NA		15	14405618	3.82172	NA	NA
5	7336891	−2.87498	NA		15	14423742	3.06346	ENSORLG00020012841	NA
5	7336968	−2.87498	NA		15	14746351	3.2905	NA	NA
5	7587556	−2.62917	NA		15	14860064	3.16613	NA	NA
5	7588457	−2.50118	NA		15	14899383	3.10133	NA	NA
5	7690003	−2.48908	NA		15	15832773	−2.42583	NA	NA
5	8697058	2.92609	NA		15	17832874	−2.3993	NA	NA
5	9173581	2.93992	NA		15	18056042	−2.41355	NA	NA
5	9885210	−2.76942	NA		15	18437184	−2.42777	NA	NA
5	10380083	−2.47768	NA		15	18437224	−2.42777	NA	NA
5	10499769	−2.9262	NA		15	18437237	−2.42777	NA	NA
5	10499803	−2.58288	NA		15	18916304	3.12068	NA	NA
5	10506824	−3.14543	NA		15	20030237	−2.43152	NA	NA
5	10507867	−3.56263	NA		15	20462181	−2.45345	NA	NA
5	11087884	3.08968	NA		15	20604752	−2.40051	NA	NA
5	14416934	2.93971	NA		15	20605022	−2.47953	NA	NA
5	14881290	3.50577	NA		15	21019777	−2.61002	NA	NA
5	15081362	3.29502	NA		15	21040332	−2.41375	NA	NA
5	15121540	−2.76318	NA		15	21040355	−2.41375	NA	NA
5	18525604	2.92194	NA		15	21156187	−2.42874	NA	NA
5	18525623	2.92194	NA		15	21201249	−2.88019	NA	NA
5	18526657	3.16074	NA		15	21229439	−3.01134	ENSORLG00020003415	NA
5	18530049	3.0905	NA		15	21234464	−2.61001	NA	NA
5	19850805	−2.77557	ENSORLG00020004654		15	21234469	−2.61001	NA	NA
5	30068878	3.23108	NA		15	21234473	−2.61001	NA	NA
6	9335491	3.39924	NA	NA	15	21234529	−2.61001	NA	NA
6	9395525	3.21945	NA	NA	15	21381646	−2.45827	NA	NA
6	9686396	−2.59362	NA	NA	15	21477695	2.91587	NA	NA
6	9707081	−2.48188	NA	NA	15	21552475	3.27129	NA	NA
6	9951008	−2.48377	NA	NA	15	21567330	3.56997	NA	NA
6	9951207	−2.73866	NA	NA	15	21574560	3.47588	NA	NA
6	9955010	−2.4218	NA	NA	15	21613944	3.60682	ENSORLG00020002803	NA
6	9985470	−2.49731	NA	NA	15	23932250	3.15307	NA	NA
6	9986098	−2.60319	NA	NA	15	25726183	−2.57556	NA	NA
6	10876573	−2.51632	NA	NA	15	25898046	3.40371	NA	NA
6	10877050	−2.51632	NA	NA	15	25912884	−2.40364	NA	NA
6	12051178	3.17813	NA	NA	15	26919055	2.95253	NA	NA
6	12067942	2.99572	NA	NA	16	1160672	−2.68585	NA	
6	14889892	3.14467	NA	NA	16	2007210	3.09933	NA	
6	14954960	3.44245	NA	NA	16	4656765	−2.75038	NA	
6	15321067	2.9458	NA	NA	16	5779343	−2.59133	NA	
6	16849513	−2.40799	NA	NA	16	8780893	3.2136	NA	
6	17318725	2.93865	NA	NA	16	10639578	3.44697	NA	
6	17856520	3.09307	NA	NA	16	12860295	3.04516	NA	
6	17856553	3.09307	NA	NA	16	13060262	3.41707	NA	
6	17856555	3.09307	NA	NA	16	13258319	3.08268	ENSORLG00020020675	
6	17960889	3.0259	NA	NA	16	13962510	2.95723	NA	
6	17966606	3.14914	NA	NA	16	15050411	3.06127	NA	
6	17976677	3.29392	NA	NA	16	15052428	3.00992	NA	
6	19404598	3.4252	NA	NA	16	15096416	3.08002	NA	
6	19803977	−2.69807	NA	NA	16	15259862	3.13095	NA	
6	19806439	−2.93216	NA	NA	16	15270756	3.35703	NA	
6	21809983	−3.09694	NA	NA	16	15297397	3.00408	NA	
6	22397064	3.05086	NA	NA	16	15377338	3.06629	NA	
6	23524606	3.06885	NA	NA	16	15778102	3.00026	NA	
6	24935543	3.35007	NA	NA	16	15780555	3.08673	NA	
7	2328615	3.22203	NA	NA	16	15780557	3.08673	NA	
7	2338156	3.03214	NA	NA	16	15805935	3.04196	NA	
7	5446083	−2.64155	NA	NA	16	15805968	3.04196	NA	
7	5446110	−2.64155	NA	NA	16	16254318	2.91925	NA	
7	5451156	−2.47451	NA	NA	16	16452486	3.03852	NA	
7	5838670	−2.46993	NA	NA	16	18241186	3.01425	NA	
7	10216199	2.97324	NA	NA	16	22374041	3.10151	NA	
7	11731231	3.08248	NA	NA	16	24312398	−2.52106	NA	
7	11801302	3.2997	ENSORLG00020008217	NA	16	24314163	−2.64977	NA	
7	11802198	3.28421	NA	NA	16	24314281	−2.63566	NA	
7	11824322	2.95843	NA	NA	17	5206696	−2.56283	NA	NA
7	11831303	2.97024	NA	NA	17	5210445	−2.94141	ENSORLG00020021876	NA
7	11843423	3.08593	NA	NA	17	5213139	−2.88442	NA	NA
7	11843441	3.08593	NA	NA	17	5609876	−2.52339	NA	NA
7	11843458	3.08593	NA	NA	17	6737147	−2.71449	NA	NA
7	11844353	3.08593	NA	NA	17	6742828	−2.66507	NA	NA
7	11848663	−2.864	NA	NA	17	6744134	−2.47047	NA	NA
7	11853067	−2.80516	NA	NA	17	6744441	−2.81787	NA	NA
7	11899543	3.35743	NA	NA	17	6754667	−2.55623	NA	NA
7	11918218	3.8347	NA	NA	17	6754793	−2.40881	NA	NA
7	11936256	3.69563	NA	NA	17	6754870	−2.46353	NA	NA
7	11940188	4.00496	NA	NA	17	6812749	−2.62059	NA	NA
7	12214771	3.34499	NA	NA	17	7309145	−2.51387	NA	NA
7	12615828	2.93042	NA	NA	17	7331692	−2.66115	NA	NA
7	12893999	3.1219	NA	NA	17	7361837	−2.41727	NA	NA
7	13023707	3.28708	NA	NA	17	8149810	−3.52504	NA	NA
7	13616037	3.05464	NA	NA	17	8154520	−3.17885	NA	NA
7	13945603	3.03119	NA	NA	17	8159008	−2.88356	NA	NA
7	14315027	3.04774	ENSORLG00020003852	NA	17	8160188	−2.59267	NA	NA
7	14317959	3.37892	NA	NA	17	8160219	−2.60501	NA	NA
7	14318068	3.37892	NA	NA	17	8160428	−2.75394	NA	NA
7	14318373	3.37892	NA	NA	17	8689397	2.93992	ENSORLG00020019098	NA
7	14320869	3.468	NA	NA	17	8726836	−2.62273	ENSORLG00020018945	NA
7	14321145	3.34991	NA	NA	17	8916291	2.95857	NA	NA
7	14322215	3.17939	NA	NA	17	8924152	−2.59273	NA	NA
7	14349066	3.15884	ENSORLG00020003816	NA	17	13466863	2.95911	NA	NA
7	14349116	3.15884	NA	NA	17	13466904	2.95911	NA	NA
7	14350769	3.15884	NA	NA	17	20491533	2.96651	NA	NA
7	14351368	2.9735	NA	NA	17	20570907	3.06568	NA	NA
7	14371524	2.99781	NA	NA	17	20571271	3.06568	NA	NA
7	14374385	2.98756	ENSORLG00020003684	NA	17	20571578	3.4181	NA	NA
7	14434378	−2.6759	NA	NA	17	20572061	3.4181	NA	NA
7	14441227	−2.48457	ENSORLG00020003401	NA	17	20572215	3.4181	NA	NA
7	14568167	4.00582	NA	NA	17	20572276	3.33562	NA	NA
7	14568282	4.00582	NA	NA	17	20572460	3.33562	NA	NA
7	14607767	3.45271	ENSORLG00020001640	NA	17	20573669	3.13893	NA	NA
7	14626870	3.53941	NA	NA	17	20573672	3.13893	NA	NA
7	14646532	−2.8464	NA	NA	17	20573721	3.13893	NA	NA
7	14646533	−2.8464	NA	NA	17	20574477	3.13893	NA	NA
7	14679402	2.94265	NA	NA	17	20580112	2.93631	NA	NA
7	14888006	−2.4194	NA	NA	17	20583220	2.93631	NA	NA
7	14957855	4.02557	NA	NA	17	20590199	3.02415	NA	NA
7	14995758	3.47792	NA	NA	17	20590411	2.98859	NA	NA
7	14996050	3.47792	NA	NA	17	20592582	3.03368	NA	NA
7	15002070	2.91736	ENSORLG00020001510	NA	17	20593096	3.38808	NA	NA
7	15087442	3.52343	NA	NA	17	20596188	3.15065	NA	NA
7	15563148	3.39306	NA	NA	17	20596759	3.0871	NA	NA
7	15565325	3.28071	NA	NA	17	20896365	3.27944	ENSORLG00020014582	NA
7	15583815	3.12112	NA	NA	17	23187526	2.97904	NA	NA
7	15585004	3.12094	NA	NA	18	2540664	2.98319	ENSORLG00020020880	
7	15589730	3.14844	NA	NA	18	2547100	3.01739	NA	
7	15602643	2.98075	NA	NA	18	6439015	2.95428	NA	
7	16720933	−2.6407	NA	NA	18	10703385	−2.40086	NA	
7	16724143	−3.24661	NA	NA	18	11157077	−2.47058	NA	
7	16725267	−3.24661	NA	NA	18	11805432	−2.41669	NA	
7	16728080	−3.23931	NA	NA	18	11841478	−2.51344	NA	
7	16728253	−3.23931	NA	NA	18	11842298	−2.58889	NA	
7	16728411	−3.23931	NA	NA	18	11846625	−2.66002	NA	
7	16737998	−2.94975	NA	NA	18	11849058	−2.52226	NA	
7	16846152	3.44677	ENSORLG00020020210	NA	18	11857673	−2.43935	NA	
7	17570111	−2.50586	NA	NA	18	11863024	−2.45039	NA	
7	17578094	−2.45055	NA	NA	18	12023155	−2.63623	NA	
7	18140389	3.15175	NA	NA	18	16328790	−2.42167	ENSORLG00020007275	^a^
7	19366223	4.30326	NA	NA	18	21386768	3.90428	NA	
7	19409414	3.62653	NA	NA	18	24286904	3.00145	NA	
7	19575673	3.29008	ENSORLG00020015678	NA	19	1209150	2.94597	ENSORLG00020008605	
7	19866127	3.69013	NA	NA	19	2659414	−2.72229	NA	
7	19980863	3.2333	NA	NA	19	2660754	2.93414	ENSORLG00020006194	
7	20146749	2.92089	NA	NA	19	2688016	−2.53915	NA	
7	20214391	3.46387	NA	NA	19	3673554	−3.12158	NA	
7	20280017	3.42126	NA	NA	19	4694910	3.08771	NA	
7	20330833	4.03386	NA	NA	19	5685428	2.95572	NA	
7	20334814	3.7211	NA	NA	19	6728129	2.95163	NA	
7	20341562	3.32575	NA	NA	19	7554924	3.29507	NA	
7	20343191	3.32575	NA	NA	19	7554927	3.29507	NA	
7	20343558	3.32575	NA	NA	19	8394133	3.15269	ENSORLG00020009890	
7	20343991	3.32575	NA	NA	19	9151754	2.94854	ENSORLG00020008495	
7	20344155	3.32575	NA	NA	19	10831704	2.96827	NA	
7	21929279	2.93269	NA	NA	19	13436299	−2.60198	NA	
7	25265222	2.98405	NA	NA	19	16309715	−2.47119	NA	
7	27048648	−2.40282	NA	NA	19	16586821	3.01519	NA	
7	30164759	−2.55582	NA	NA	19	17686137	2.97777	NA	
7	30164778	−2.56195	NA	NA	20	4080154	3.09035	NA	^a^
7	31394436	−2.93348	NA	NA	20	5969963	−2.46686	NA	^a^
8	2030180	−3.01714	NA		20	8223848	−2.54392	NA	
8	4510871	3.05532	NA		20	8659955	−2.6037	NA	
8	6978044	−2.70269	NA		20	9267466	−2.49312	NA	
8	9361677	3.09165	NA		20	9267482	−2.49312	NA	
8	10122427	−2.84158	NA		20	9269823	−2.76034	NA	
8	10122428	−2.84158	NA		20	9276492	−2.45575	NA	
8	10123473	−2.56979	NA		20	9289199	−2.41705	NA	
8	10123598	−2.56979	NA		20	9291118	−2.45274	NA	
8	10123645	−2.56979	NA		20	9304955	−2.59507	NA	
8	10125176	−2.49396	ENSORLG00020020372		20	9308027	−2.87033	NA	
8	10125816	−2.49396	NA		20	9308054	−2.87033	NA	
8	10129097	−2.4524	NA		20	9329269	−2.97517	NA	
8	10406824	−2.47414	NA		20	9331041	−3.00414	NA	
8	11743632	−2.45122	NA		20	9331100	−3.00414	NA	
8	11847811	−2.93118	NA		20	9331103	−3.00414	NA	
8	14853298	2.92005	ENSORLG00020011786		20	9331195	−3.0646	NA	
8	15060746	−3.00754	ENSORLG00020011649		20	9331201	−3.0646	NA	
8	17790280	3.26965	NA		20	9334469	−2.63928	NA	
8	18591926	−2.6219	NA		20	9336189	−2.7424	NA	
8	18596984	−2.55521	NA		20	9341292	−2.51237	NA	
8	18622568	3.38435	NA		20	9680949	−2.44385	NA	
8	18636990	3.49022	NA		20	9681047	−2.68356	NA	
8	18737934	3.79032	ENSORLG00020006044		20	9681451	−2.56436	NA	
8	19137999	3.44623	NA		20	9681465	−2.56436	NA	
8	19199856	3.00905	NA		20	9694036	−2.51876	NA	
8	19199857	3.00905	NA		20	10574960	−2.42812	NA	
8	19256870	3.76517	NA		20	10620038	−2.54145	NA	
8	19445425	−2.45867	NA		20	10633410	−2.73063	NA	
8	19511287	2.96004	NA		20	10633517	−2.67996	NA	
8	19601854	3.46175	NA		20	10637900	−2.48357	NA	
8	19615698	2.92422	NA		20	10697483	−2.48365	NA	
8	19619920	3.71196	NA		20	10697488	−2.48365	NA	
8	19620218	3.71196	NA		20	10713521	−2.92612	NA	
8	19620220	3.71196	NA		20	10752929	−2.54729	NA	
8	19622520	3.91377	NA		20	10756652	−2.56951	NA	
8	19624063	3.56563	NA		20	10758293	−2.41241	NA	
8	19626531	3.4204	NA		20	10759000	−2.552	NA	
8	19627571	3.55339	NA		20	10760581	−3.20253	NA	
8	19628508	3.55339	NA		20	10760643	−3.20253	NA	
8	19628511	3.55339	NA		20	11028786	−2.56353	NA	
8	19629488	3.22144	NA		20	11030810	−3.6614	NA	
8	19630038	3.61602	NA		20	11794307	−2.74382	NA	
8	19631986	3.61602	ENSORLG00020005017		20	11794835	−2.96518	NA	
8	19762311	3.23439	NA		20	11794928	−3.39706	NA	
8	19768356	3.32025	NA		20	11794980	−3.39706	NA	
8	19768489	3.40023	NA		20	11913129	3.42125	NA	
8	19768698	3.40023	NA		20	14558489	3.0072	NA	
8	19771035	3.39026	NA		20	15613363	−2.55869	ENSORLG00020014726	
8	19773573	3.50026	NA		20	18670751	−2.4642	NA	
8	19773591	3.50026	NA		20	18674373	−2.51285	NA	
8	19773740	3.50026	NA		20	18674691	−2.88179	NA	
8	19773793	3.50026	NA		20	18675919	−2.64127	ENSORLG00020009561	
8	19773808	3.49899	NA		20	18676101	−2.7011	NA	
8	19773942	3.49899	NA		20	18676357	−2.55356	NA	
8	19774597	3.5759	ENSORLG00020004763		20	18701660	−2.43818	NA	
8	19776033	4.01869	NA		20	18731315	−2.4389	NA	
8	19776350	4.11057	NA		20	18731410	−2.53866	NA	
8	19777051	3.78419	NA		20	22592162	−2.54194	NA	
8	19777081	3.78419	NA		20	23027776	−2.65227	NA	
8	19777097	3.78419	NA		21	3767021	−2.43053	ENSORLG00020018929	
8	19777987	3.78419	NA		21	3767068	−2.61856	NA	
8	19778047	3.78419	NA		21	4699383	−2.47865	NA	
8	19778053	3.78419	NA		21	14016398	2.949	NA	^a^
8	19779222	3.78419	ENSORLG00020004759		21	14220603	3.05932	NA	^a^
8	19779235	3.78419	NA		21	14261091	−2.57267	NA	^a^
8	19779257	3.78419	NA		21	14323966	3.3034	NA	^a^
8	19779402	3.78329	NA		21	14493737	3.09693	NA	^a^
8	19779445	3.30077	NA		21	14526415	3.1405	NA	^a^
8	19779802	4.1393	NA		21	18432843	−3.30264	NA	
8	19779807	4.1393	NA		21	18432976	−2.53928	NA	
8	19779815	4.1393	NA		21	19548458	3.42893	ENSORLG00020022334	
8	19779824	4.0987	NA		21	19548577	3.42893	NA	
8	19779825	4.0987	NA		21	19553123	3.05162	ENSORLG00020022335	
8	19779944	3.87832	NA		21	19554800	3.19021	NA	
8	19780254	3.67636	NA		21	19555557	3.31087	NA	
8	19780619	3.12886	NA		21	19556960	3.60272	NA	
8	19782255	3.03512	NA		21	19563198	2.95362	NA	
8	19899157	3.1381	NA		21	19570523	4.1063	NA	
8	19899562	3.02627	NA		21	19583315	3.22377	ENSORLG00020022337	
8	19914577	3.16171	ENSORLG00020004611		21	19590889	3.52202	NA	
9	3481874	−2.52965	ENSORLG00020009592		21	19728761	2.96223	NA	
9	5772468	−2.74444	NA		21	19740632	3.08004	NA	
9	6208324	3.1663	NA		21	19751101	3.67445	NA	
9	7425008	−2.46344	NA		21	19933748	2.95993	NA	
9	8684449	3.1823	NA		21	19933896	2.95993	NA	
9	8815268	3.04572	NA		21	19954535	3.13225	NA	
9	9047329	−2.65205	NA		21	19969093	3.49793	ENSORLG00020022517	
9	10514009	3.10077	NA		21	19973166	3.586	NA	
9	11199250	2.93884	NA		21	20040713	3.57074	ENSORLG00020022537	
9	11851828	2.97084	NA		21	20278215	3.14262	ENSORLG00020022561	
9	15196347	3.26858	NA		21	21257026	−2.42993	NA	
9	15196378	3.26858	NA		21	21757335	−2.68478	NA	
9	15762980	2.96354	NA		21	21757533	−2.80709	NA	
9	19048297	3.01785	NA		21	21919590	−2.60221	NA	
9	19861130	2.99653	NA		21	21919821	−2.60221	NA	
9	23434909	−2.66516	ENSORLG00020021419		21	25651570	2.93537	NA	
9	24675037	3.12466	NA		22	2821345	−2.86884	NA	
10	4421030	−2.60904	NA		22	6311060	2.91569	ENSORLG00020000562	
10	4493397	−2.85116	NA		22	6475623	2.96527	ENSORLG00020018299	
10	4761983	−2.40597	NA	^a^	22	7977161	3.58706	NA	
10	4762756	−2.40597	NA	^a^	22	7980484	3.3464	NA	
10	4796316	−2.53021	NA	^a^	22	8011920	−2.47422	NA	
10	4935420	−2.58191	NA	^a^	22	8011983	−2.47422	NA	
10	4941166	−2.96812	NA	^a^	22	8012029	−2.47422	NA	
10	4941308	−2.96812	NA	^a^	22	8012044	−2.47422	NA	
10	4960928	−2.88136	NA	^a^	22	8012054	−2.47422	NA	
10	4965705	−2.77101	NA	^a^	22	8012130	−2.47422	NA	
10	4966330	−2.51007	NA	^a^	22	8012138	−2.47422	NA	
10	4967594	−2.46938	NA	^a^	22	8012160	−2.47422	NA	
10	4968579	−2.59495	NA	^a^	22	8012317	−2.47422	NA	
10	4980174	−2.76621	NA	^a^	22	8012373	−2.47422	NA	
10	4987872	−2.56639	NA	^a^	22	8012393	−2.47422	NA	
10	4990300	−2.47309	NA	^a^	22	8012476	−2.47422	NA	
10	4998771	−2.8754	NA	^a^	22	8440774	−2.8423	NA	
10	4999970	−2.51726	NA	^a^	22	8449498	−2.76927	NA	
10	5013558	−2.70479	NA	^a^	22	8449504	−2.76927	NA	
10	5028938	−2.57939	NA	^a^	22	9327812	3.28081	NA	
10	5031619	−2.75587	NA	^a^	22	14091333	−2.58909	NA	
10	5098765	−2.66401	NA	^a^	22	14091346	−2.58909	NA	
10	5103944	−2.70101	NA	^a^	22	16405116	−2.4461	NA	
10	5127983	−2.56526	NA	^a^	22	26305367	3.05618	NA	^a^
10	5129665	−3.0268	NA	^a^	22	26305404	3.05618	NA	^a^
10	5132067	−2.83419	NA	^a^	22	26314740	3.06529	NA	^a^
10	5135980	−3.02162	NA	^a^	22	26356147	3.02723	NA	^a^
10	5136666	−2.66214	NA	^a^	23	13723748	−2.49401	NA	
10	5139335	−2.89309	NA	^a^	23	14031524	−2.48149	NA	
10	5139459	−2.89309	NA	^a^	23	15194468	2.99907	NA	
10	5139555	−2.43544	NA	^a^	23	15227385	3.0585	NA	
10	5142448	−2.89587	NA	^a^	23	16374156	3.01299	NA	
10	5142455	−2.89587	NA	^a^	23	16916218	3.44237	NA	
10	5142536	−2.89587	NA	^a^	23	17955674	2.92755	NA	
10	5144039	−2.89587	NA	^a^	24	970756	−2.41728	NA	
10	5146805	−2.50695	NA	^a^	24	1329285	3.01078	NA	
10	5153030	−3.15192	NA	^a^	24	3101994	−2.78305	NA	
10	5155638	−2.45525	NA	^a^	24	3590033	2.94471	NA	
10	5171575	−2.57425	NA	^a^	24	4567814	−3.09954	NA	
10	5172254	−2.43543	NA	^a^	24	4643423	−2.5658	NA	
10	5173591	−2.43543	NA	^a^	24	4663269	−2.59778	NA	
10	5174058	−2.66656	NA	^a^	24	4663482	−2.59778	NA	
10	5174061	−2.66656	NA	^a^	24	8338597	2.99709	ENSORLG00020009609	
10	5174063	−2.66656	NA	^a^	24	9415975	3.41579	NA	
10	5174073	−2.66656	NA	^a^	24	9417257	3.32962	NA	
10	5176119	−2.60008	NA	^a^	24	9973700	−2.49787	NA	
10	5327513	−2.70837	NA	^a^	24	10019144	−2.6664	NA	
10	5327554	−2.70837	NA	^a^	24	10019177	−3.10378	NA	
10	5327661	−2.70837	NA	^a^	24	10019178	−3.10378	NA	
10	5327964	−2.70837	NA	^a^	24	10019863	−2.40062	NA	
10	5328240	−2.70837	NA	^a^	24	10742023	−2.64674	NA	
10	5329204	−2.58134	NA	^a^	24	10742047	−2.60883	NA	
10	5330115	−2.58134	NA	^a^	24	10742104	−2.77057	NA	
10	5330225	−2.58134	NA	^a^	24	10742238	−2.62722	NA	
10	5393892	−2.70933	ENSORLG00020006311	^a^	24	10747643	−2.43761	NA	
10	5394035	−3.01867	NA	^a^	24	10750344	−2.73841	NA	
10	6986836	−3.13971	NA		24	10750372	−3.62097	NA	
10	6987881	−2.46129	NA		24	10750377	−2.7428	NA	
10	6990097	3.25445	NA		24	10760540	−2.70422	NA	
10	7309653	−2.62504	NA		24	10760637	−2.51578	NA	
10	7313675	−2.5032	NA		24	10760791	−2.64038	NA	
10	7314857	−2.65461	NA		24	10760817	−2.64038	NA	
10	7342396	−2.57793	NA		24	10761088	−2.64038	NA	
10	10017939	−3.24068	NA		24	10761090	−2.64038	NA	
10	10018596	−3.24068	NA		24	10761898	−2.64038	NA	
10	13297423	2.92712	NA		24	10762484	−2.64038	NA	
10	13303697	2.93029	NA		24	11253551	2.95595	NA	
10	13306442	4.00382	NA		24	11420462	3.14569	NA	
10	13332163	3.38349	NA		24	11446269	−3.22178	NA	
10	13351584	3.10871	NA		24	14778367	3.05007	NA	
10	13351862	3.10871	NA		24	14805406	3.01608	NA	
10	13354347	3.14752	NA		24	14811477	2.96456	NA	
10	13372450	3.13854	NA		24	14848034	3.22783	NA	
10	13375925	3.18319	NA		24	15070933	3.08913	NA	
10	13376104	3.12315	NA		24	15141063	3.29162	NA	
10	14768408	3.48858	NA		24	15230658	3.0034	NA	
10	18173014	−2.54917	NA		24	19091521	−2.66626	NA	^a^
10	18173088	−2.54917	NA						
10	19457673	−2.75476	NA						
10	19458039	−2.75476	NA						
10	24917779	2.94111	NA						

^a^ Centromere region were inferred based on the synteny block positions of HdrR and HNI references using ntSynt (Table A3), and overlap determined within 1 Mb of the centromere regions.

**TABLE A10:** Annotated genes located in regions with selection signatures and phenotypic associations identified in previous studies.

Chromosome	Range (Mb)	Representative gene	Ensembl gene ID (ASM223467)	Representative GO term	Evolutionary scenario	Phenotype association	References
6	18.15–18.18	*1‐sf, choriogenin h, cpt1b*	ENSORLG00000010134, ENSORLG00000010160, ENSORLG00000010086	Egg coat, sperm‐egg recognition, regulation of reproductive process	Introgression (EJ→Osak)	Egg envelope	Murata and Kinoshita (2022)
7	11.73–15.60	NA	NA	NA	Selective sweep (iHS)	Fin morphology	Kon et al. (2026)
10	13.75–13.90	*pcdh2aa1, si:ch73‐379j16.2*	ENSORLG00000021994, ENSORLG00000028208	Cell–cell adhesion, calcium ion binding	High genetic diversity & sex differentiation	Sexual dimorphism (anal fin length)	Kawajiri et al. (2015)
11	17.43–17.44	*hoxa13a*	ENSORLG00000005043	NA	Selective sweep (iHS)	Fin morphology	Corcoran et al. (2026)
14	15.10–15.13	*ar*	ENSORLG00000009520	NA	Selective sweep (iHS)	Male‐specific fin morphology and courtship behavior	Ogino et al. (2023)
15	8.05–8.10	*rasgrp3*	ENSORLG00000023586	Calcium ion binding	High genetic diversity	Sexual dimorphism (Anal fin length)	Kawajiri et al. (2015)
18	4.25–4.30, 6.85–6.95	*dysf*, *si:dkey‐183c6.8*, *si:dkey‐183c6.7*, *syt4*	ENSORLG00000003427, ENSORLG00000004084, ENSORLG00000004069, ENSORLG00000004098	Calcium ion binding	High genetic diversity	Female mate choice (spawning latency)	Fujimoto et al. (2024)
19	6.75–6.80	*Integrin alpha‐M‐like*, *aldoa*	ENSORLG00000006367, ENSORLG00000006425, ENSORLG00000022047	Cell–cell adhesion	High genetic diversity	Sexual dimorphism (anal fin length and papillary processes)	Kawajiri et al. (2014, 2015)
20	21.30–21.35	*rdh12l, si:dkey‐61p9.11, olpks*	ENSORLG00000012778, ENSORLG00000012785, ENSORLG00000012793	Fatty acid biosynthetic process	High genetic diversity	Male courtship frequency (approaching)	Fujimoto et al. (2024)
21	12.78–12.93	NA	ENSORLG00000013916, ENSORLG00000024077, ENSORLG00000023929, ENSORLG00000025608, ENSORLG00000028977, ENSORLG00000025011, ENSORLG00000013895	Trace‐amine receptor activity	Introgression (EJ→Osak)	NA	NA
24	9.50–9.55	*Adhesion G protein‐coupled receptor F7*	ENSORLG00000015595	NA	High genetic diversity	Fin morphology (anal fin length, dorsal fin length, caudal fin length)	Kawajiri et al. (2014)
